# Recognition of *O*^6^-benzyl-2′-deoxyguanosine by a perimidinone-derived synthetic nucleoside: a DNA interstrand stacking interaction

**DOI:** 10.1093/nar/gkt488

**Published:** 2013-07-08

**Authors:** Ewa A. Kowal, Rahul R. Lad, Pradeep S. Pallan, Elizabeth Dhummakupt, Zdzislaw Wawrzak, Martin Egli, Shana J. Sturla, Michael P. Stone

**Affiliations:** ^1^Department of Chemistry, Center in Molecular Toxicology, Vanderbilt-Ingram Cancer Center, Center in Structural Biology, Vanderbilt University, Nashville, TN 37235, USA, ^2^Department of Health Sciences and Technology, Institute of Food, Nutrition and Health, ETH Zürich, CH-8092 Zürich, Switzerland, ^3^Department of Biochemistry, Vanderbilt University, Nashville, TN 37232, USA and ^4^Department of Health Sciences and Technology, Synchrotron Research Center, Northwestern University, 9700 S Cass Ave, Argonne, IL 60439, USA

## Abstract

The 2′-deoxynucleoside containing the synthetic base 1-[(2*R*,4*S*,5*R*)-4-hydroxy-5-(hydroxymethyl)-tetrahydrofuran-2-yl)-1*H*-perimidin-2(3*H*)-one] (dPer) recognizes in DNA the *O*^6^-benzyl-2′-deoxyguanosine nucleoside (*O*^6^-Bn-dG), formed by exposure to *N*-benzylmethylnitrosamine. Herein, we show how dPer distinguishes between *O*^6^-Bn-dG and dG in DNA. The structure of the modified Dickerson–Drew dodecamer (DDD) in which guanine at position G^4^ has been replaced by *O*^6^-Bn-dG and cytosine C^9^ has been replaced with dPer to form the modified *O*^6^-Bn-dG:dPer (DDD-XY) duplex [5′-d(C^1^G^2^C^3^X^4^A^5^A^6^T^7^T^8^Y^9^G^10^C^11^G^12^)-3′]_2_ (X = *O*^6^-Bn-dG, Y = dPer) reveals that dPer intercalates into the duplex and adopts the *syn* conformation about the glycosyl bond. This provides a binding pocket that allows the benzyl group of *O*^6^-Bn-dG to intercalate between Per and thymine of the 3′-neighbor A:T base pair. Nuclear magnetic resonance data suggest that a similar intercalative recognition mechanism applies in this sequence in solution. However, in solution, the benzyl ring of *O*^6^-Bn-dG undergoes rotation on the nuclear magnetic resonance time scale. In contrast, the structure of the modified DDD in which cytosine at position C^9^ is replaced with dPer to form the dG:dPer (DDD-GY) [5′-d(C^1^G^2^C^3^G^4^A^5^A^6^T^7^T^8^Y^9^G^10^C^11^G^12^)-3′]_2_ duplex (Y = dPer) reveals that dPer adopts the *anti* conformation about the glycosyl bond and forms a less stable wobble pairing interaction with guanine.

## INTRODUCTION

**Chart 1.**
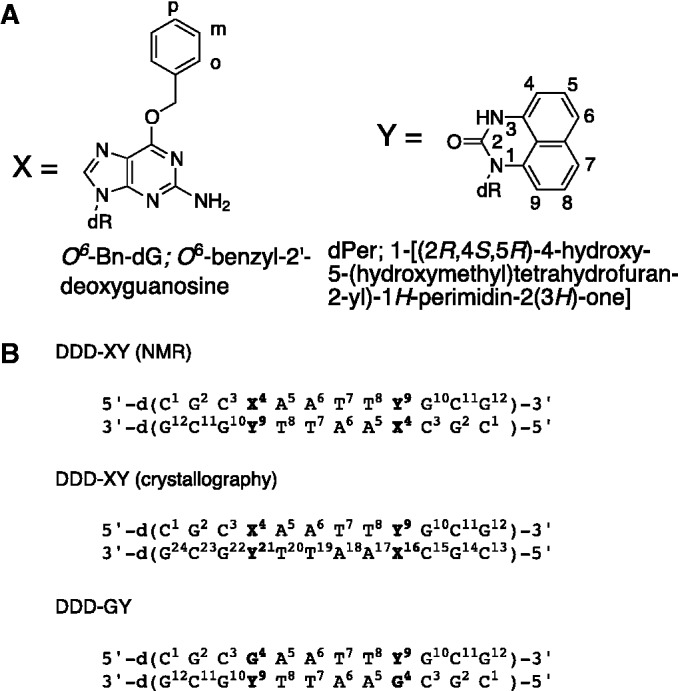
(**A**) Structures of *O*^6^-Bn-dG and dPer. (**B**) Sequences and numbering of the DDD-XY, DDD-XY and DDD-GY DDDs. The dodecamer exhibits pseudo-dyad symmetry, and in NMR spectra, both strands are numbered identically from nucleotides C^1^ to G^12^. The two strands are not symmetry related in the crystalline lattice and in crystallographic structures, the nucleotides are numbered from C^1^ to G^12^ in the first and from C^13^ to G^24^ in the second strand.

The alkylation of deoxyguanosine in DNA at the *O*^6^ position, exemplified by exposure to *N*-benzylmethylnitrosamine and the formation of *O*^6^-benzyl-2′-deoxyguanosine nucleoside (*O*^6^-Bn-dG), is cytotoxic (1) and mutagenic (2,3). The *O*^6^-Bn-dG lesion is representative of bulky DNA adducts involved in the initiation of gene mutations (4,5). It predominantly causes G→A transitions (6,7) and is observed in human cells (8). The development of synthetic nucleotides as chemical probes enabling site-specific reporting of such DNA damage is of interest (9–22). The 2′-deoxynucleoside containing the synthetic base 1-[(2*R*,4*S*,5*R*)-4-hydroxy-5-(hydroxymethyl)-tetrahydrofuran-2-yl)-1*H*-perimidin-2(3*H*)-one] (dPer; Chart 1) recognizes the *O*^6^-Bn-dG in DNA (23). Analyses of DNA containing *O*^6^-Bn-dG or dPer paired opposite each other or natural bases have demonstrated that the *O*^6^-Bn-dG:dPer pair is more stable than any pairing of the damaged base opposite any natural base, or of dPer opposite a natural base (23). Developing an understanding of the structural basis for *O*^6^-Bn-dG recognition is critical to further developing nucleosides such as dPer to recognize these mutagenic lesions.

**Figure 1.**
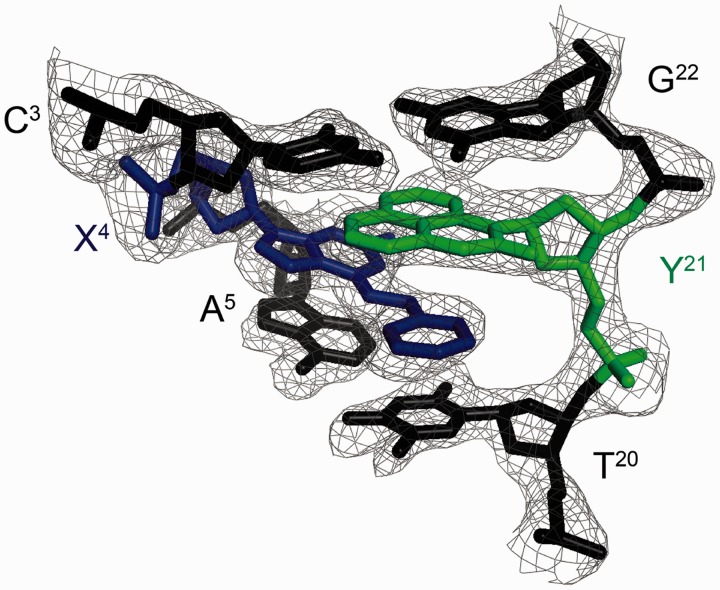
The DDD-XY duplex in the region of the C^3^:G^22^, X^4^:Y^21^ and A^5^:T^20^ base pairs, showing the electron density map. The dPer nucleotide recognizes the benzyl group of *O*^6^-Bn-dG (X^4^) via a stacking interaction such that the benzyl ring intercalates between the T^20^ and Y^21^ bases.

Herein, we explain the molecular basis by which dPer distinguishes between *O*^6^-Bn-dG and dG. When deoxyguanosine at position G^4^ of the Dickerson–Drew dodecamer (DDD) (24) was replaced by *O*^6^-Bn-dG, and deoxycytosine C^9^ was replaced with dPer to form the modified *O*^6^-Bn-dG:dPer (DDD-XY) duplex [5′-d(C^1^G^2^C^3^X^4^A^5^A^6^T^7^T^8^Y^9^G^10^C^11^G^12^)-3′]_2_ (X = *O*^6^-Bn-dG, Y = dPer) (Chart 1), dPer intercalated into the duplex and adopted the *syn* conformation about the glycosyl bond. This provides a binding pocket that allows the benzyl group of *O*^6^-Bn-dG to intercalate between Per and thymine of the 3′-neighbor A:T base pair. In contrast, when deoxycytosine at position C^9^ is replaced with dPer to form the duplex containing the dG:dPer base pair (DDD-GY) [5′-d(C^1^G^2^C^3^G^4^A^5^A^6^T^7^T^8^Y^9^G^10^C^11^G^12^)-3′]_2_ (Y = dPer), dPer adopts the *anti* conformation about the glycosyl bond and forms a less stable wobble pair with deoxyguanosine.

## MATERIALS AND METHODS

### Materials

The unmodified 5′-dCGCGAATTCGCG-3′ (DDD) was synthesized by the Midland Reagent Company (Midland, TX) and purified by anion-exchange high-performance liquid chromatography. The modified oligodeoxynucleotides were synthesized using an ABI 394 DNA synthesizer (Applied Biosystems, Foster City, CA) or a Mermade 9 DNA synthesizer (Bioautomation, Irving, TX) using β-cyanoethyl phosphoramidite chemistry. The dPer (23) and *O*^6^-Bn-dG phosphoramidites were prepared as described previously (25). The yields of the stepwise coupling reactions were monitored by trityl cation response. The oligodeoxynucleotides were removed from the resin by treating with 18 M (saturated) ammonium hydroxide for 1.5 h at 25°C. After filtration, the resulting solutions were heated at 55°C for 6 h to deprotect the oligodeoxynucleotides. All oligodeoxynucleotides were purified by semi-preparative reverse-phase high-performance liquid chromatography (Phenomenex, Phenyl-Hexyl, 5 µm, 250 mm × 10.0 mm) equilibrated with 0.1 M triethylammonium acetate (pH 7.0). The oligodeoxynucleotides were desalted by passing over Sephadex G-25 and characterized by matrix-assisted laser-desorption-ionization time-of-flight mass spectrometry. The concentrations of single-stranded oligodeoxynucleotides were estimated by ultraviolet (UV) absorbance at 260 nm on the basis of an extinction coefficient of 1.11 × 10^5 ^M^−1 ^cm^−1^, which was not adjusted for the presence of the modified bases (26). The oligodeoxynucleotides were annealed by heating to 80°C for 15 min and then cooled to room temperature.

### Thermal denaturation studies

Melting temperatures were measured with a Varian Cary 100 Bio spectrophotometer operated at 260 nm. The buffer used for measurements contained 10 mM sodium phosphate, 50 μM Na_2_EDTA and 0.1 M NaCl (pH 7). The temperature was increased from 10 to 80°C at a rate of 0.5°C/min. Melting temperatures were calculated from first-order derivatives of the absorbance versus temperature profiles. The concentration of DNA was 1.5 µM.

### Crystallizations and data collection of the DDD-XY duplex

Crystallization trials were performed with the Nucleic Acid Mini screen (27) (Hampton Research, Aliso Viejo, CA). The hanging drop vapor diffusion technique was used. DNA was desalted and prepared in water at 1.2 mM concentration. Droplets with volume 2 µl of a 1:1 mixture of sample and mini-screen buffer were equilibrated against 0.75 ml of 35% 2-methyl-2,4-pentanediol (MPD) at 18°C. Two crystals were obtained and found to be suitable for data collection. The first was crystalized from 10% MPD, 40 mM sodium cacodylate, 12 mM spermine tetra-HCl and 80 mM KCl, 20 mM BaCl_2_ (pH 7.0). The second was crystallized from 10% MPD, 40 mM sodium cacodylate, 12 mM spermine tetra-HCl, 40 mM LiCl and 80 mM SrCl_2_ (pH 7.0). Crystals were mounted in nylon loops and frozen in liquid nitrogen. Diffraction data were collected in a cold nitrogen stream on beamline 21-ID-F at LS-CAT, APS (Argonne National Laboratory, Argonne, IL) for both crystals. Single-wavelength anomalous dispersion (SAD) data were collected on the 21-ID-D beamline for the first crystal at the energy corresponding to absorption peak for the Ba atom. All data were processed with the program HKL2000 (28) and XDS (29).

### Crystal structure determination and refinement of the DDD-XY duplex

The PHENIX (30) software was used to calculate phases and initial placing of the model into the electron density map from the SAD data for the first crystal, which was crystallized with BaCl_2_. Then, initial refinement of the model was performed with the Computer and Network Systems (CNS) (31) program (National Science Foundation), setting aside 5% randomly selected reflections for calculating the R_free_. Rigid body refinement and simulated annealing were performed. After several cycles of refinement, the emergent model was used as the starting model for phasing by molecular replacement methods for a data set obtained from the second crystal. Multiple rounds of coordinate refinements and simulated annealing led to an improved model for which sum (2*F*_o_-*F*_c_) and difference (*F*_o_-*F*_c_) Fourier electron density maps were generated. At a later stage solvent, water molecules were added on the basis of Fourier 2*F*_o_-*F*_c_ sum and *F*_o_-*F*_c_ difference electron density maps. Water molecules were accepted based on the standard distances and B-factor criteria. Further, structure refinement was performed using the program REFMAC in the Collaborative Computational Project Number 4 software suite (CCP4) (32). Geometry and topology files were generated for the *O*^6^-Bn-dG and dPer modified bases, and anisotropic temperature factor refinement was performed afterward. The programs TURBO-FRODO (33) and COOT (34) were used to display electron density maps. Helicoidal analysis was performed using the CURVES+ web server (35).

### Nuclear magnetic resonance

The DDD-XY and DDD-GY modified duplexes were prepared at concentrations of 0.56 mM and 0.53 mM, respectively. The samples were prepared in 10 mM NaH_2_PO_4_, 0.1 M NaCl and 50 µM Na_2_EDTA (pH 7.0). To observe non-exchangeable protons, the samples were exchanged with D_2_O. The DDD-GY duplex was dissolved in D_2_O. The DDD-XY duplex was dissolved in 9:1 D_2_O:CD_3_CN. For the observation of exchangeable protons, the samples were dissolved in 9:1 H_2_O:D_2_O. ^1^H NMR spectra for DDD-XY duplex were recorded at 900 MHz at 10°C and 500 MHz at 7°C. ^1^H nuclear magnetic resonance (NMR) spectra for DDD-GY duplex were recorded at 800 MHz in D_2_O at 10°C and 600 MHz in 9:1 H_2_O:D_2_O at 5°C. Chemical shifts were referenced to water. Data were processed using TOPSPIN software (Bruker Biospin Inc., Billerica, MA). The nuclear Overhauser effect spectroscopy (NOESY) (36,37) and double quantum filtered correlation spectroscopy (DQF-COSY) (38) spectra in D_2_O were collected at 10°C; NOESY experiments were conducted at mixing times of 150, 200 and 250 ms with a relaxation delay of 2.0 s. The NOESY spectra of the modified samples in H_2_O were collected with a 250 ms mixing time, with a relaxation delay of 1.5 s. Water suppression was performed using the WATERGATE pulse sequence (39).

### NMR experimental restraints

The NOESY spectra were processed using the TOPSPIN software (Bruker Biospin Inc., Billerica, MA), and the spectral data were evaluated using the program SPARKY (40) to obtain the cross-peak assignments. The intensities of cross-peaks were measured by volume integrations. Experimental intensities were combined with intensities obtained from complete relaxation matrix analysis (CORMA) of starting model to generate a hybrid intensity matrix (41,42). The intensities were converted to distances with the program MARDIGRAS, which refined the hybrid intensity matrix (43). Calculations were performed using 150, 200 and 250 ms mixing time data and 2, 3 and 4 ns isotropic correlation times. Evaluation of the resulting distance data allowed creation of upper and lower bound distance restraints that were used in restrained molecular dynamics (rMD) calculations. Additional empirical base pair, backbone and deoxyribose pseudorotation restraints for base pairs not proximal to the sites of modification were obtained from canonical values derived from B-DNA (44).

### rMD calculations

An unmodified B type DNA model was used as a starting structure. The cytosine at position C^9^ in each strand was replaced by dPer with INSIGHT II (Accelrys Inc., San Diego, CA). Partial charges for Per were calculated with the B3LYP/6-31 G* basis set in GAUSSIAN (45). The starting structure was energy minimized for 1000 cycles. A simulated annealing protocol (46) was used for the rMD calculations, which were conducted with the parm99 force field, using the program AMBER (47). Force constants of 32 kcal mol^−^^1 ^Å^−^^2^ were applied for distance restraints. The generalized Born model (48) was used for solvation. The salt concentration in all calculations was 0.1 M. Coupling of the molecule to the bath temperature was used to control the temperature during simulated annealing. First, calculations were performed for 20 ps (20 000 steps) by the following protocol: During steps 0–1000, the system was heated from 0 to 600 K with a coupling of 0.5 ps. During steps 1001–2000, the system was kept at 600 K. The system was then cooled from 600 to 100 K during steps 2001–18 000 with a coupling of 4 ps. Further cooling from 100 to 0 K occurred during steps 18 001–20 000 with a coupling of 1 ps. After initial cycles of refinement a longer 100 ps (100 000 steps), calculation was performed by the following protocol: During steps 0–5000, the system was heated from 0 to 600 K with a coupling of 0.5 ps. During steps 5001–10 000, the system was kept at 600 K. The system was cooled from 600 to 100 K during steps 10 001–90 000 with a coupling of 4 ps. Additional cooling from 100 to 0 K occurred during steps 90 001–100 000 with a coupling of 1 ps. Structure coordinates were saved after each cycle and were subjected to potential energy minimization. Nine refined structures calculated from the different starting structures were chosen based on the lowest deviations from the experimental distance and dihedral restraints and energy minimized to obtain an average structure. CORMA (41,42) was used to compare intensities calculated from these emergent structures with the distance restraints. Helicoidal analysis was performed using the CURVES+ web server (35).

### Data deposition

The complete structure factor and final coordinates were deposited in the Protein Data Bank (www.rcsb.org): the PDB ID code for the DDD-XY duplex is 4HQI and for the DDD-GY duplex the PDB ID code is 2M11. Supplementary Table S1 contains the CIF file.

## RESULTS

### Thermodynamic studies

The unfolding of the DDD, DDD-XY and DDD-GY duplexes was examined by temperature-dependent UV spectroscopy. The *T*_M_ values were determined by taking the first derivatives of the resulting UV melting curves. The melting temperature for the DDD was 45°C, for DDD-GY duplex was 28°C and for the DDD-XY duplex was 33°C. Notably, for the DDD-XY duplex, the presence of the dPer base complementary to *O*^6^-Bn-dG increased the *T*_M_ of by 5°C as compared with the DDD-GY duplex, in agreement with prior observations that dPer thermodynamically discerns the presence of *O*^6^-Bn-dG (23).

### Structure of the DDD-XY duplex

Two crystals suitable for data collection were obtained. The first was crystalized from the buffer containing 20 mM BaCl_2_. This crystal diffracted to 1.95 Å. The diffraction data were processed in space group *P*2_1_2_1_2_1_ (orthorhombic). The processing and refinement parameters are shown in Supplementary Table S2. It was not possible to complete the phasing utilizing molecular replacement approaches. Instead, the experimental phases were obtained from SAD data collected at the energy corresponding to absorption peak for Ba. From these data, phasing was accomplished, as was the initial placement of the model into the electron density map. Then, initial refinement of the model was performed, setting aside 5% randomly selected reflections for calculating the R_free_. Rigid body refinement and simulated annealing produced a structure that was in good agreement with the experimental electron density. A second crystal, crystallized from the buffer containing 80 mM SrCl_2_, diffracted at the greater resolution of 1.7 Å, also in space group *P*2_1_2_1_2_1_ (orthorhombic). The data from the second crystal were phased using molecular replacement methods in which the structure from the first crystal was used as a starting model. Multiple rounds of coordinate refinements and simulated annealing led to an improved structure for which sum (2*F*_o_-*F*_c_) and difference (*F*_o_-*F*_c_) Fourier electron density maps were generated.

A total of 49 water molecules were added on the basis of Fourier 2*F*_o_–*F*_c_ sum and *F*_o_–*F*_c_ difference electron density maps. These were accepted based on the standard distances and B-factor criteria. One Sr^2+^ ion was identified in the electron density map based on its low B-factor and the characteristic geometry, as well as one spermine. The cell parameters (a = 26.38, b = 36.77, c = 77.65, α = 90.0, β = 90.0, γ = 90.0, Supplementary Table S3) were atypical for the DDD duplex. The volume of the unit cell was greater. The overall structure of the DDD-XY duplex is shown with waters, Sr^2 + ^, and a spermine molecule in Supplementary Figure S1. Although no electron density was observed for the 5′-terminal bases C^1^ and C^13^, and thus their positions could not be determined with certainty, the 3′-terminal bases G^12^ and G^24^ rotated out of the duplex toward the major groove of adjacent molecules. The crystal data collection and refinement statistics are compiled in Supplementary Table S3.

**Figure 2.**
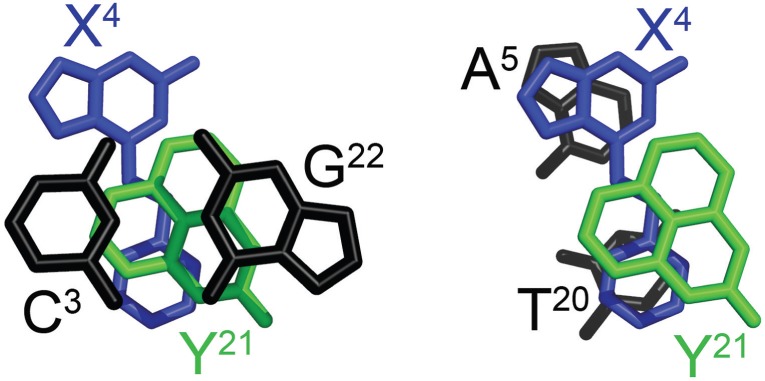
Stacking interactions for the *O*^6^-Bn-dG:dPer (DDD-XY) duplex. (Left panel) Stacking of the C^3^:G^22^ base pair (black) above X^4^ (blue) and Y^21^ (green). (Right panel) Stacking of the X^4^:Y^21^ pair (in blue and green, respectively) above base pair A^5^:T^20^ (black). The benzyl ring of *O*^6^-Bn-dG (X^4^) is stabilized by intercalation between T^20^ and Y^21^ (dPer) bases.

Figure 1 shows the DDD-XY duplex in the region of the C^3^:G^22^, X^4^:Y^21^ and A^5^:T^20^ base pairs. Both the *O*^6^-Bn-G and Per bases fit well into the electron density map. *O*^6^-Bn-dG remained in the *anti* conformation about the glycosyl bond. In contrast, the dPer nucleoside adopted the *syn* conformation. The intercalation of the Per base created a binding pocket into which the benzyl ring of the *O*^6^-Bn-dG base was inserted. The benzyl ring of the *O*^6^-Bn-G base also formed a stacking interaction with T^20^ of the 5′-neighbor A^5^:T^20^ base pair. The simultaneous insertion of both dPer and the benzyl ring of the *O*^6^-Bn-dG base increased the helical rise between neighboring base pairs C^3^:G^22^ and A^5^:T^20^ to 9.5 Å, as compared with the anticipated rise of ∼6.8 Å in B-DNA (Supplementary Figure S2). It also unwound the duplex. For the modified duplex, the twist at base pairs C^3^:G^22^ and X^4^:Y^21^ was −15°, whereas for the unmodified duplex it was 25°, a change of 40° (Supplementary Figure S2). For the dPer phosphodiester backbone angles α and γ changes of ∼210° were observed compared with the unmodified duplex (Supplementary Figures S3 and S4). The χ angle for dPer was in the range of 60–80°, which was consistent with the *syn* conformation (Supplementary Figure S5). The intercalation of the Per base, which was located between *O*^6^-Bn-G and the 5′ neighbor cytosine in both strands, increased the ξ angles of C^3^ and C^15^ by 90° (Supplementary Figure S5). Watson–Crick base pairing at the neighbor base pairs C^3^:G^22^ and A^5^:T^20^ was not disturbed. Figure 2 illustrates the stacking between the benzyl ring of *O*^6^-Bn-dG and dPer.

### NMR spectroscopy of the DDD-XY duplex

**Figure 3.**
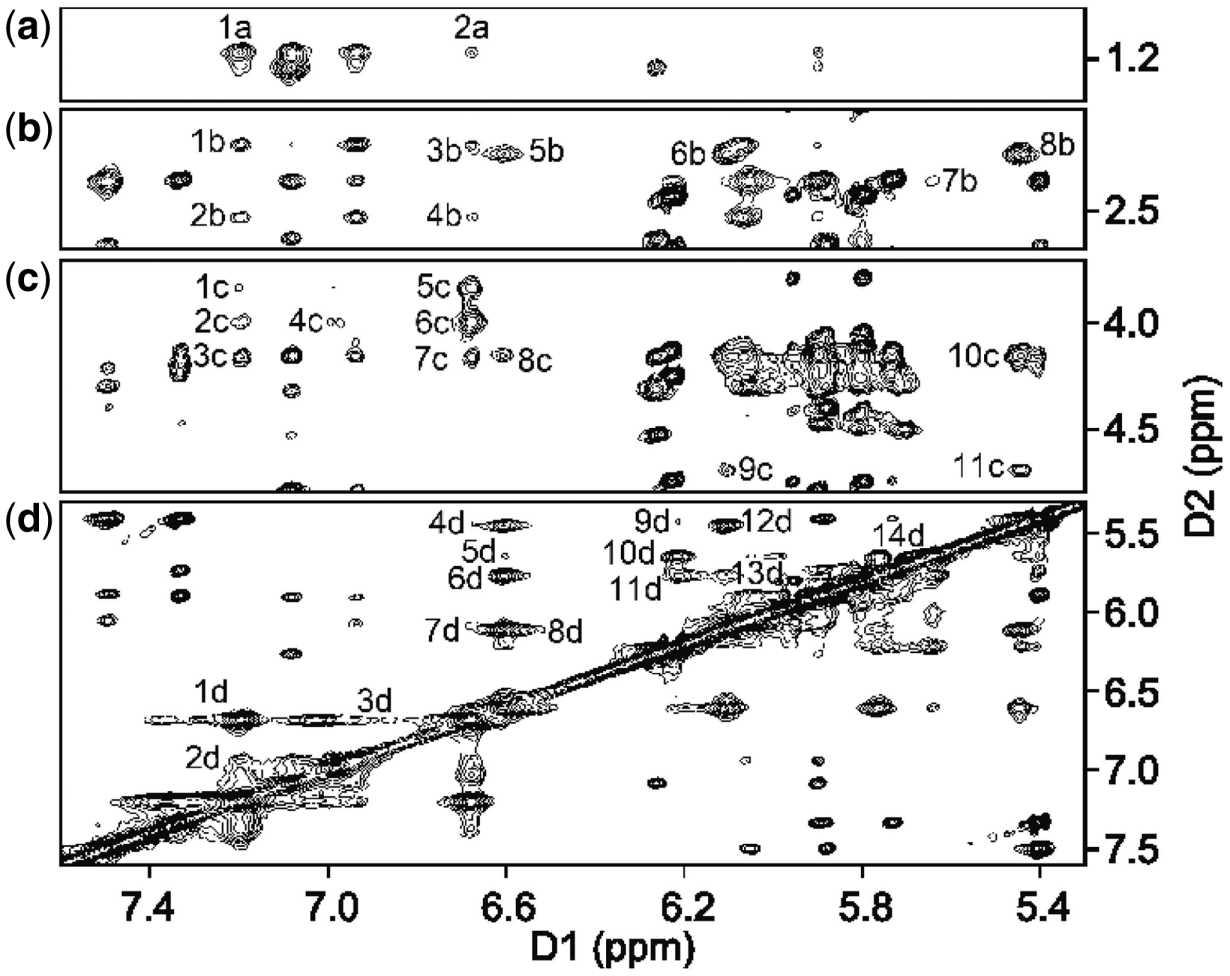
The NOESY spectrum for the DDD-XY duplex, showing the assignments of the *O*^6^-Bn-dG and dPer protons. The cross-peaks in (**a**) are assigned as follows: 1a, T^8^ CH_3_→X^4^ H_meta_; 2a T^8^ CH_3_→X^4^ H_ortho_. The cross-peaks in (**b**) are assigned as follows:1b, T^8^ H2′→X^4^ H_meta_; 2b, T^8^ H2′′→X^4^ H_meta_; 3b, T^8^ H2′→X^4^ H_ortho_; 4b, T^8^ H2''→X^4^ H_ortho_; 5b, Y^9^ H2′/H2''→Y^9^ H8; 6b, Y^9^ H2′/H2′′→Y^9^ H9; 7b, C^3^ H2′→Y^9^ H6; 8b, Y^9^ H2′/H2′′→Y^9^ H1′. The cross-peaks in (**c**) are assigned as follows: 1c, X^4^ Hm_1_→X^4^ H_meta_; 2c, X^4^ Hm_2_→X^4^ H_meta_; 3c, Y^9^ H4′→X^4^ H_meta_; 4c, X^4^ Hm_2_→X^4^ H_para_; 5c, X^4^ Hm_1_→X^4^ H_ortho_; 6c, X^4^ Hm_2_→X^4^ H_ortho_; 7c, Y^9^ H4′→X^4^ H_ortho_; 8c, Y^9^ H4′ →Y^9^ H8; 9c, Y^9^ H3′→Y^9^ H9; 10c, Y^9^ H4′→Y^9^ H1′; 11c, Y^9^ H3′→Y^9^ H1′. The cross-peaks in (**d**) are assigned as follows: 1d, X^4^ H_ortho_→X^4^ H_meta_; 2d, X^4^ H_para_→X^4^ H_meta_; 3d, X^4^ H_ortho_→X^4^ H_para_; 4d, Y^9^ H1′→Y^9^ H8; 5d, Y^9^ H6→Y^9^ H8; 6d, Y^9^ H7→Y^9^ H8; 7d, Y^9^ H9→X^4^ H_ortho_; 8d, Y^9^ H9→Y^9^ H8; 9d, Y^9^ H4→Y^9^ H5; 10d, Y^9^ H6→Y^9^ H5; 11d, Y^9^ H7→Y^9^ H5; 12d, Y^9^ H1′→Y^9^ H9; 13d, Y^9^ H7→Y^9^ H9. The spectrum was collected at 10°C at 900 MHz, using a 250 ms mixing time.

To ascertain whether the crystallographic structure for the DDD-XY duplex was representative of the solution structure, a series NMR spectroscopy experiments were conducted. These proved to be challenging, as the resonances associated with the *O*^6^-Bn-dG:dPer pairing interaction exhibited severe line broadening. The source of the line broadening was not identified but may be attributed to rotation of the benzyl ring of *O*^6^-Bn-dG at a rate intermediate on the NMR time scale. However, it was observed that the addition of 10% CD_3_CN to the solvent as an organic modifier resulted in significant line narrowing to the resonances associated with the *O*^6^-Bn-dG:dPer interaction. The addition of the organic modifier did not otherwise affect the NMR spectrum of the duplex, and the duplex structure of the DNA was maintained (‘vide infra’). The *O*^6^-Bn-dG benzyl protons were observed as three signals between 6.6 and 7.4 ppm (Figure 3). All gave cross-peaks with the X^4^ methylene protons Hm_1_, Hm_2_ (1 c, 2 c, 4 c, 5 c, 6 c, Figure 3c). This indicated that in the presence of the organic modifier, rotation of the benzyl ring was rapid on the NMR time scale. The resonance located farthest downfield at 7.3 ppm was assigned as X^4^ H_meta_, whereas the resonance located farthest upfield at 6.7 ppm was assigned as X^4^ H_ortho_. The X^4^ H_para_ proton was assigned at 7 ppm. Cross-peaks were observed between H_ortho_→H_meta_ (1d, Figure 3d), H_meta_→H_para_ (2d, Figure 3d), H_ortho_→H_para_ (3d, Figure 3d). Interstrand cross-peaks between the X^4^ benzyl ring and T^8^ CH_3_, H2′, H2′′ were observed (1a, 2a, 1b, 2b, 3b, 4b, Figure 3a and b). A weak cross-peak between Y^9^ H9 and X^4^ H_ortho_ was observed (7d, Figure 3d). The dPer (Y^9^) resonances were observed upfield from the benzyl ring protons of *O*^6^-Bn-dG (X^4^). Cross-peaks between dPer hydrogens are shown in Figure 3d (4d-6d, 8d-14d). Additional cross-peaks between the dPer base and its deoxyribose were identified (5b-8b, 8c-11c, Figure 3b and c). One weak interstrand cross-peak was identified between C^3^ H2′ and Y^9^ H6 (7b, Figure 3b).

In the sequential NOE connectivity between base aromatic and deoxyribose H1′ protons (49,50), a number of the anticipated NOEs were weak (Supplementary Figure S6). These included the C^3^ H6→C^3^ H1′, the C^3^ H1′→X^4^ H8, the X^4^ H8→X^4^ H1′ and the X^4^ H1′→A^5^ H8. Also, the T^7^ H1′→T^8^ H6, T^8^ H6→T^8^ H1′, T^8^ H1′→Y^9^ H8, Y^9^ H8→Y^9^ H1′ and Y^9^ H1′→G^10^ H8 NOEs were weak. The magnitude of the Y^9^ H^8^→Y^9^ H1′ NOE was consistent with the *syn* conformation of the dPer nucleotide about the glycosyl bond. In the imino and amino proton regions of the spectrum, the Y^9^ imino proton could not be identified (Supplementary Figure S7). This was attributed to rapid exchange with solvent. Thus, in the sequential connectivity of the base imino protons (51), no T^8^ N3H→Y^9^ imino or Y^9^ imino→G^10^ N1H NOE was observed. The A^5^ H2→T^8^ N3H NOE was weak as compared with the A^6^ H2→T^7^ N3H NOE.

### Structure of the DDD-GY duplex

**Figure 4.**
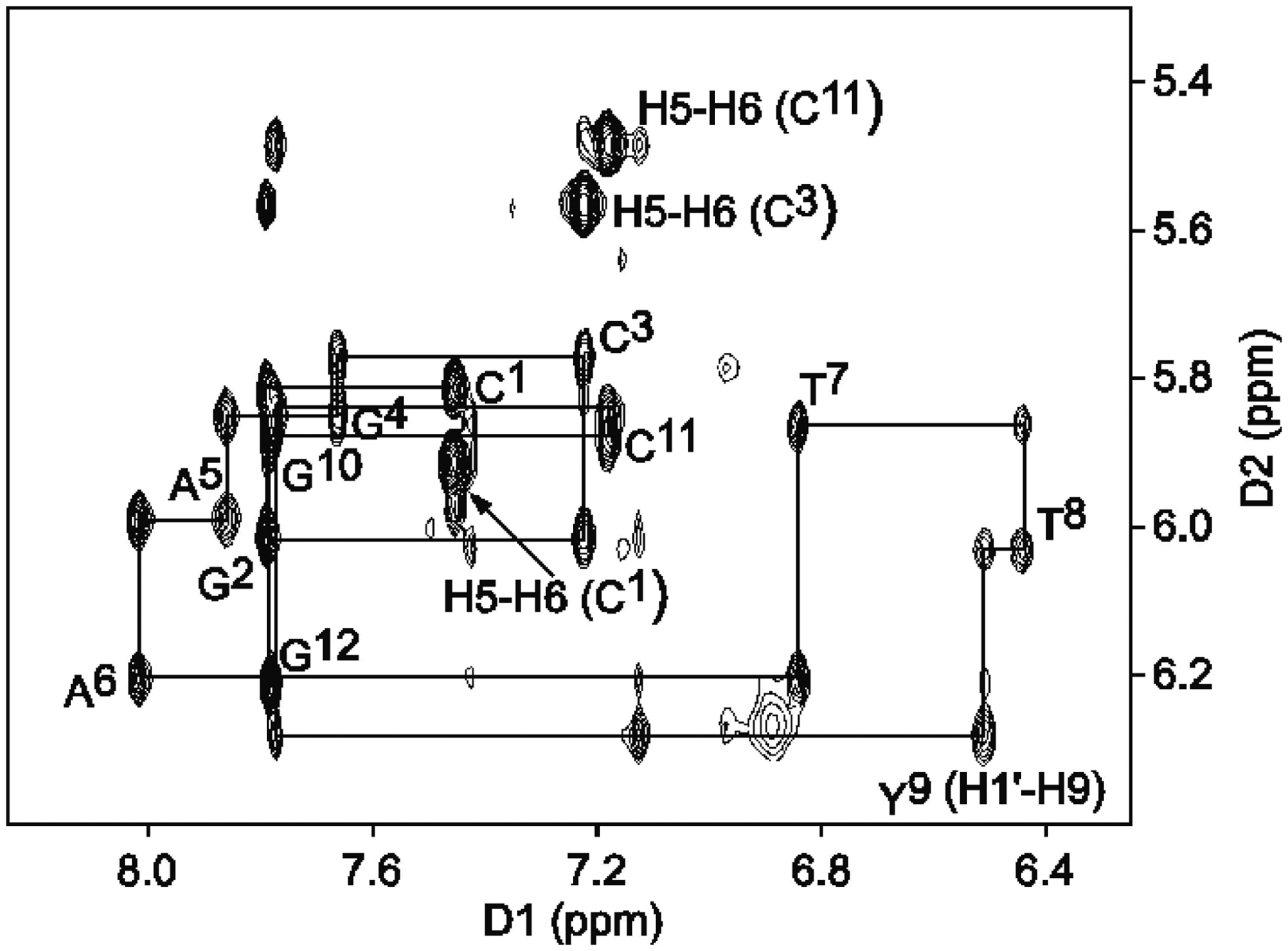
The NOESY spectrum of the DDD-GY duplex, showing sequential NOEs between the aromatic and anomeric protons from C^1^ to G^12^. The spectrum was collected at 10°C at 800 MHz, using a 250 ms mixing time.

To determine the basis by which dPer differentially recognized the *O*^6^-Bn-dG adduct versus dG, the structure of dPer placed opposite dG (DDD-GY) was determined. This duplex was not amenable to crystallographic analysis. However, it was possible to complete a structural determination by NMR. Assignments between aromatic protons of the base to deoxyribose H1′ protons are shown in Figure 4. At the G^4^:Y^9^ base pair, the C^3^ H6→C^3^ H1′, C^3^ H1′→G^4^ H8, G^4^ H8→G^4^ H1′, G^4^ H1′→A^5^ H8 and A^5^ H8→A^5^ H1′ cross-peaks were observed and were of normal intensities. Also, the T^8^ H6→T^8^ H1′, T^8^ H1′→Y^9^ H9, Y^9^ H9→Y^9^ H1′, Y^9^ H1′→G^10^ H8 and G^10^ H8→G^10^ H1′ NOEs were observed and were of normal intensities. There was a small chemical shift difference compared with unmodified duplex for the T^8^ and Y^9^ bases.

**Figure 5.**
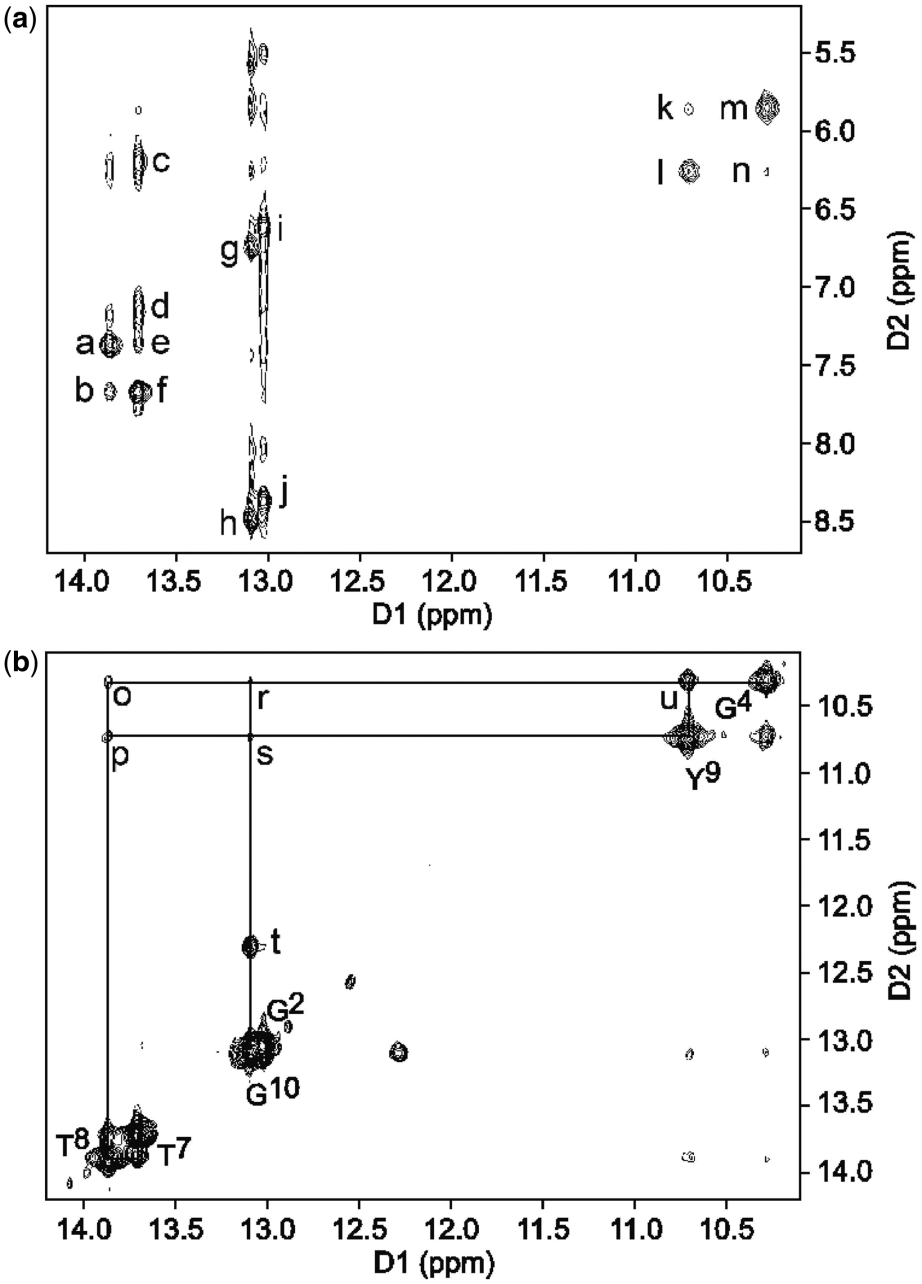
The NOESY spectrum for the DDD-GY duplex. (**a**) Interstrand NOEs between complementary bases. The cross-peaks are assigned as a, A^5^ H2→T^8^ N3H; b, A^6^ H2→T8 N3H; c, A^6^ H1′→T^7^ N3H; d, A^6^ *N^6^*H2→T^7^ N3H; e, A^5^ H2→T^7^ N3H; f, A^6^ H2→T^7^ N3H; g, C^3^ *N^2^*H1→G^10^ N1H; h, C^3^ *N^2^*H2→G^10^ N1H; i, C^11^ *N^2^*H1→G^2^ N1H; j, C^11^ *N^2^*H2→G^2^ N1H; k, G^4^ H1′→Y^9^ HN; l, Y^9^ H1′→Y^9^ HN; m, G^4^ H1′→G^4^ N1H; n, Y^9^ H1′→G^4^ N1H. (**b**) NOE connectivity for the imino protons for the base pairs G^2^:C^11^, C^3^:G^10^, G^4^:Y^9^, A^5^:T^8^, A^5^:T^7^. The cross-peaks are assigned as T^8^ N3H→T^7^ N3H, T^8^ N3H→Y^9^ HN (p), T^8^ N3H→G^4^ N1H (o), Y^9^ HN→G^10^ N1H (s), G^4^ N1H→G^10^ N1H (r), G^2^ N1H→G^10^ N1H and Y^9^ HN→G^4^ N1H (u). Cross-peak (t) could not be assigned. The same cross-peak was observed for the DDD-XY duplex (peak k, Supplementary Figure S7). A NOESY experiment at a shorter mixing time of 70 ms showed no change in intensity and suggested that this may be an exchange cross-peak of unknown origin. The experiment was carried out at 5°C and with a mixing time of 250 ms at 600 MHz.

The sequential NOEs between the base imino protons showed a strong cross-peak between the G^4^ N1H imino proton and the Y^9^ dPer imino proton (u, Figure 5b). The sequential connectivity of the base imino protons was thus obtained from base pairs G^2^:C^11^→C^3^:G^10^→G^4^:Y^9^→A^5^:T^8^→A^6^:T^7^ (Figure 5b). The region of the spectrum showing NOEs between the base imino and amino protons and adenine H2 protons showed cross-peaks for base pairs A^5^:T^8^, A^6^:T^7^, G^2^:C^11^, G^10^:C^3^, and it showed that G^4^ and Y^9^ formed a base pair (k, l, m, n, Figure 5a). The G^10^ cross-peak had a similar chemical shift as compared with G^2^. The G^4^ N1H and Y^9^ HN resonances were shifted upfield to 10.2 and 10.7 ppm.

**Figure 6.**
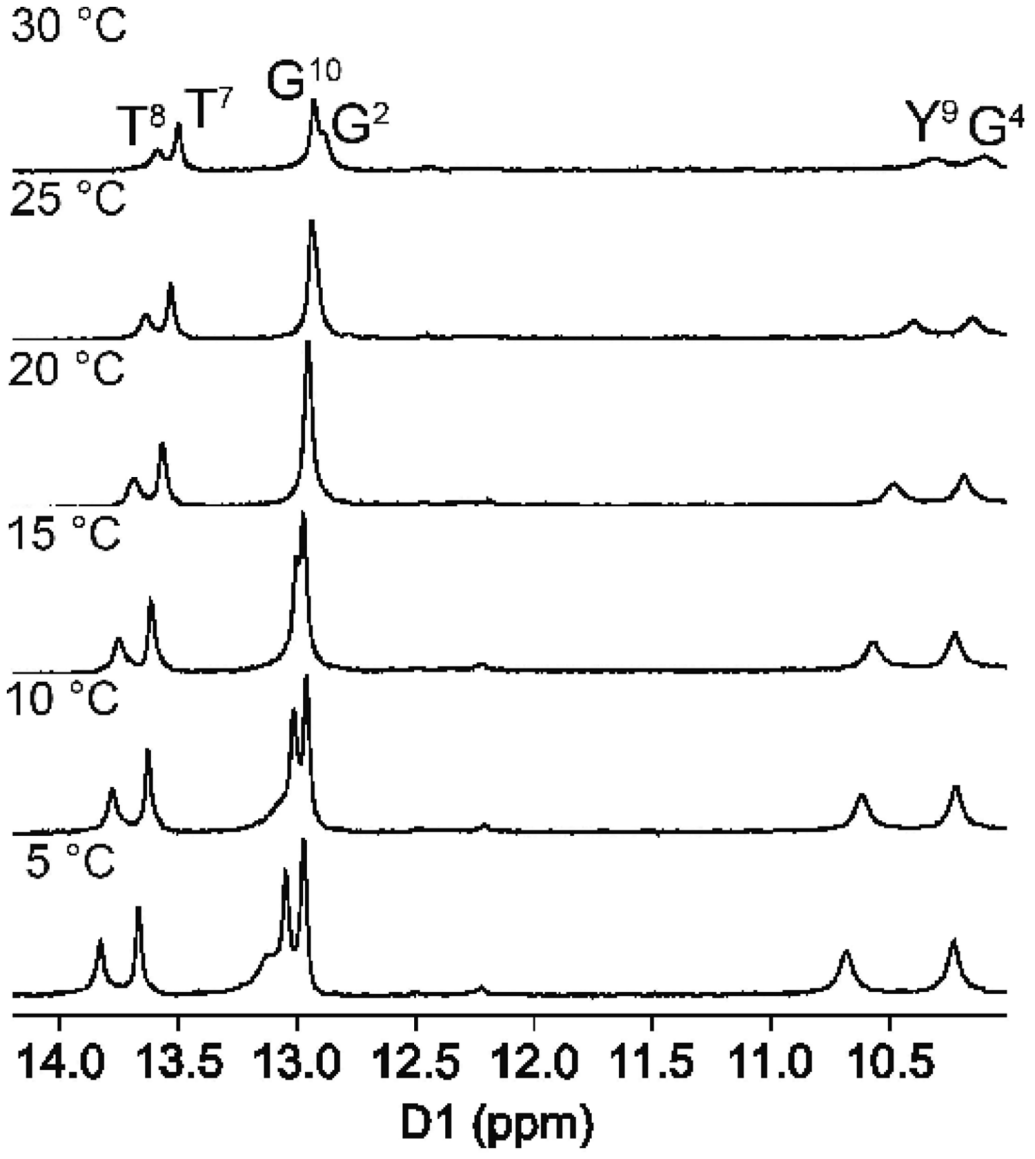
The 1D NMR spectra showing the imino proton resonances for the DDD-GY duplex as a function of temperature. The individual nucleotides are identified as superscripts. Spectra were collected at 600 MHz.

In the NMR spectrum for the DDD-GY duplex (Figure 6), two additional resonances were observed at 10.3 and 10.7 ppm, which were assigned to the G^4^ and Y^9^ bases. These were broad as compared with the other imino resonances. The chemical shift for the G^10^ N1H imino proton was similar to that of the G^2^ N1H imino proton; these two resonances remained sharp even at higher temperatures. The thymine T^8^ N3H imino resonance remained sharp at higher temperatures as compared with the T^8^ N3H imino resonance in the DDD-XY duplex (Supplementary Figure S8).

**Figure 7.**
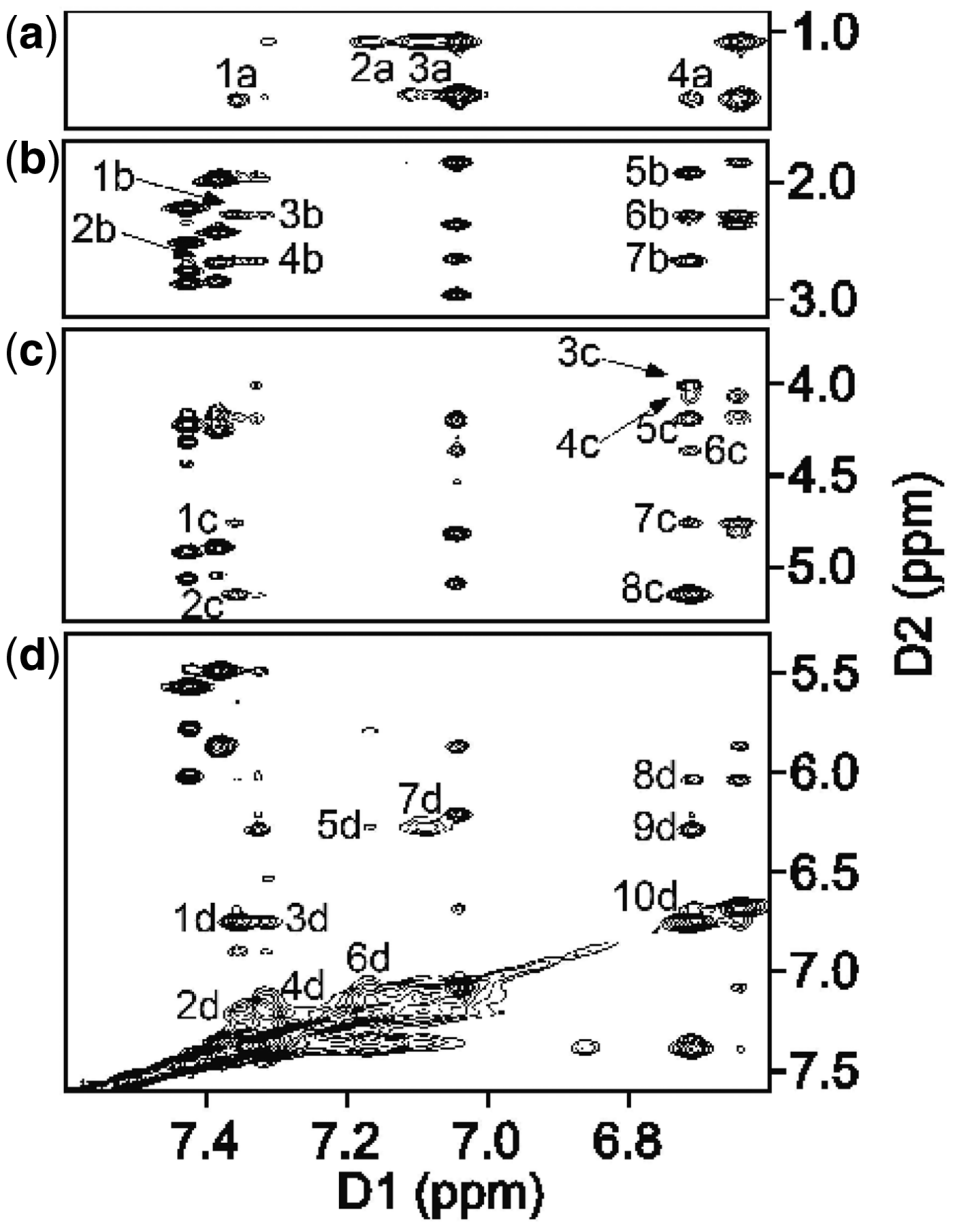
The NOESY spectrum showing dPer resonance assignments for the DDD-GY duplex. The cross-peaks in (**a**) are assigned as follows: 1a, T^8^ H2′→Y^9^ H8; 2a, T^8^ Me→Y^9^ H6; 3a, T^8^ Me→Y^9^ H5; 4a, T^8^ H2′→Y^9^ H9. The cross-peaks in (**b**) are assigned as follows: 1b, T^8^ H2′′→Y^9^ H8; 2b Y^9^ H2′′→Y^9^ H8; 3b, T^8^ H2′′→Y^9^ H7; 4b, Y^9^ H2′′→Y^9^ H7; 5b, Y^9^ H2′→Y^9^ H9; 6b, T^8^ H2′′→Y^9^ H9; 7b, Y^9^ H2′′→Y^9^ H9. The cross-peaks in (**c**) are assigned as follows: 1c, T^8^ H3′→Y^9^ H8; 2c, Y^9^ H3′→Y^9^ H8; 3c, Y^9^ H5′′→Y^9^ H9; 4c, T^8^ H5′′→Y^9^ H9; 5c, Y^9^ H5′→Y^9^ H9; 6c, Y^9^ H4′→Y^9^ H9; 7c, T^8^ H3′→Y^9^ H9; 8c, Y^9^ H3′→Y^9^ H9. The cross-peaks in (**d**) are assigned as follows: 1d, Y^9^ H9→Y^9^ H8; 3d, Y^9^ H9→Y^9^ H7; 2d, Y^9^ H6→Y^9^ H8; 4d, Y^9^ H6→Y^9^ H7; 6d, Y^9^ H5→Y^9^ H6; 5d, Y^9^ H4→Y^9^ H6; 7d, Y^9^ H4→Y^9^ H5; 10d, T^8^ H6→Y^9^ H9; 9d, Y^9^ H1′→Y^9^ H9; 8d, T^8^ H1′→Y^9^ H9. The spectrum was collected at 10°C, with 250 ms mixing time, at 800 MHz.

The assignment of the dPer aromatic protons H4, H5, H6, H7, H8 and H9 is shown in Figure 7. These were observed between 6.2 and 7.4 ppm. The cross-peak between T^8^ H1′→Y^9^ H9 was identified (10 d, Figure 7d). Based on the intensities of cross-peaks, H8 and H7 were identified. They both showed cross-peaks to H9 (1d, 3d, Figure 7d). The H6 cross-peak was identified based on its proximity to H8 and H7 and H5 (2d, 4d, 6d, Figure 7d). The H5 proton showed a cross-peak to H4 (7d, Figure 7d). This peak was broad and shifted upfield to 6.2 ppm. H9 and H8 showed cross-peaks to T^8^ H2′ (1a, 4a, Figure 7a). H6 and H5 showed cross-peaks to the T^8^ CH_3_ group (2a, 3a, Figure 7a). Additional cross-peaks between H9, H8, H7 and its deoxyribose and to T^8^ deoxyribose protons were assigned (Figure 7c).

**Figure 8.**
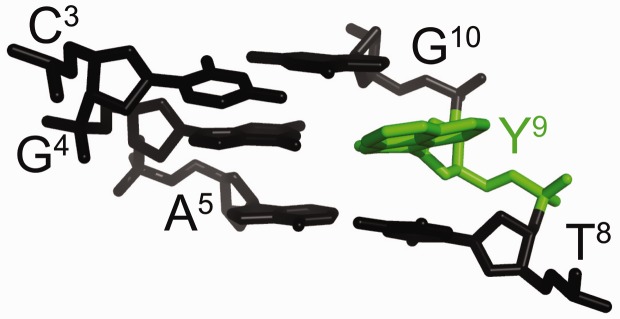
The average structure of the DDD-GY duplex, in the region of the C^3^:G^10^, G^4^:Y^9^ and A^5^:T^8^ base pairs. Base Y^9^ is shown in green. The dPer ring is oriented in the major groove. It does not disrupt the neighbor base pairs. Hydrogens are omitted for clarity.

**Figure 9.**
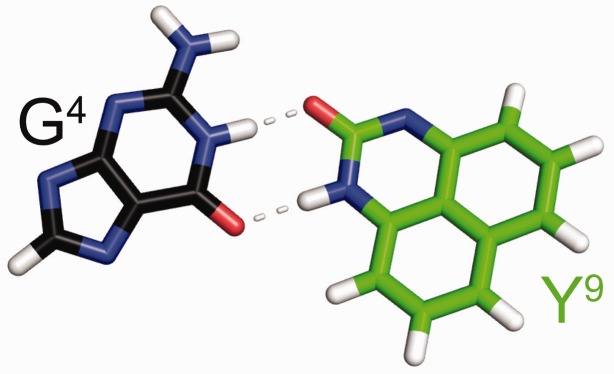
The average structure of the G^4^:Y^9^ base pair, in the DDD-GY duplex. G^4^ forms a wobble pair with the complementary dPer (Y^9^) base. The anticipated hydrogen bonds are indicated as gray dashed lines.

**Figure 10.**
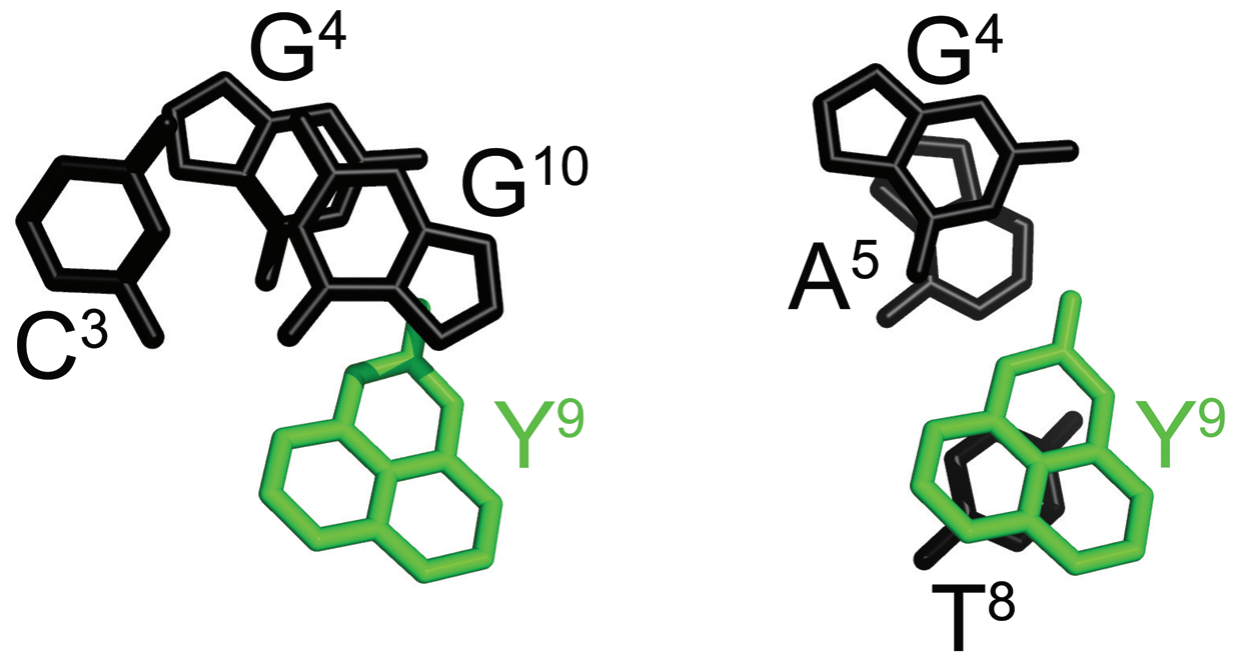
Stacking interactions for the DDD-GY duplex. (**a**) Stacking of the C^3^:G^10^ base pair (black) above the G^4^:Y^9^ base pair (green). (**b**) Stacking of G^4^ and Y^9^ (black and green, respectively) above the A^5^:T^8^ base pair (black). The dPer ring is in the major groove. The dPer (Y^9^) base stacks with T^8^.

The structure of the dG:dPer (DDD-GY) duplex was determined using a simulated annealing rMD protocol, restrained by experimental distance restraints determined from NOEs. Supplementary Table S4 shows the restraints used for rMD calculations. Nine structures were energy minimized and superimposed to obtain the average structure (Supplementary Figure S9). Supplementary Figure S10 shows these superimposed structures and the average structure. The latter was in good agreement with the experimental restraints confirmed by CORMA (52) analysis. Supplementary Table S5 shows the structural statistics. Figure 8 shows the DDD-GY duplex in the region of the C^3^:G^10^, G^4^:Y^9^ and A^5^:T^8^ base pairs. The dPer Y^9^ base formed a wobble base pair with the complementary guanine G^4^, involving two hydrogen bonds (Figure 9), which was supported by a strong cross-peak between imino protons of G^4^ and Y^9^ of opposite strands (cross-peak u, Figure 5b). The dPer ring was oriented in the major groove and adopted the *anti* conformation about the glycosyl bond. It did not disrupt neighbor base pairs. The dPer base stacked with its 5′ neighbor T^8^, but it did not stack well with its 3′ neighbor G^10^ (Figure 10). The complementary guanine, G^4^ stacked well with its 3′ neighbor A^5^, but not with C^3^. Helicoidal analysis (Supplementary Figures S11, S12, S13 and S14) revealed that the ζ angle of the dPer nucleotide increased by ∼50° compared with the unmodified duplex, which corroborated the reduced stacking between dPer (Y^9^) and the 3′ neighbor guanine (G^10^) (Supplementary Figure S14).

## DISCUSSION

The dPer synthetic nucleoside (Chart 1) recognizes *O*^6^-Bn-dG, as indicated by thermodynamic stabilization of the *O*^6^-Bn-dG:dPer interaction (23). The present studies, in the DDD sequence context, reveal a 5°C increase in the *T*_M_ of the DDD-XY as compared with the DDD-GY duplex, which corroborates the previous results. It was originally hypothesized that the ability of dPer to recognize *O*^6^-Bn-dG was due to stacking and hydrophobic interactions with the benzyl ring of the DNA adduct, combined with potential hydrogen bonding between the *anti* conformation of dPer about the glycosyl bond and the N1 and *N*^2^ nitrogen atoms of the alkylated deoxyguanosine (23). The present data provide experimental evidence for a different mode of recognition.

### dPer recognizes *O*^6^-Bn-dG via a stacking interaction

The data suggest that in this DNA sequence the presence of dPer ‘traps’ the benzyl ring of *O*^6^-Bn-dG between the dPer nucleotide and T^20^, providing a mechanism whereby dPer recognizes the *O*^6^-Bn-dG DNA damage (Figures 1 and 2). Crystallographic electron density maps show the insertion of the dPer base into the DNA provides a binding pocket for the benzyl group of *O*^6^-Bn-dG to intercalate between Per and thymine of the 3′-neighbor A:T base pair. The simultaneous insertion of Per and the benzyl group of *O*^6^-Bn-dG unwinds the duplex at the recognition site (Figure 1 and Supplementary Figure S1), as suggested by the weak sequential NOE connectivity cross-peak observed between C^3^ H1′ and X^4^ H8. Additionally, the weak cross-peak T^8^ H1′→Y^9^ H8 is consistent with an increased distance between these bases. The chemical shifts of the dPer base resonances, observed in the 5.5–6.4 ppm range (Figure 3), are consistent with the insertion of dPer into the duplex and π–π stacking with the benzyl group of *O*^6^-Bn-dG. Furthermore, the absence of the dPer imino resonance in NMR spectra indicates that the Y^9^ imino proton is in enhanced exchange with the solvent, consistent with a lack of base pairing (Supplementary Figure S7).

The orientation of the dPer base about the glycosyl bond in this DNA sequence was of interest. The *syn*-glycosyl conformation of the dPer nucleoside was observed when it was not incorporated into DNA (23). On the other hand, Gong and Sturla (23) had suggested hydrogen bonding between the N1 and *N*^2^ nitrogen atoms of the alkylated deoxyguanosine and dPer, requiring the *anti* conformation of dPer about the glycosyl bond. In the crystallographic data obtained at a resolution of 1.7 Å, when inserted into the electron density map in the *syn* conformation, the resulting crystallographic R factor was minimized. If dPer was instead inserted into the electron density map in the *anti* conformation, it did not fit well, and residual difference (*F*_o_-*F*_c_) Fourier electron density was observed. The NMR data also show a weak NOE between the dPer base H9 proton and the deoxyribose H1′ proton, consistent with dPer adopting the *syn* glycosyl torsion angle (Supplementary Figure S6). Overall, we conclude that in this DNA sequence, the insertion of dPer into the duplex is stabilized by a combination of base stacking and steric factors. Gong and Sturla (23) have observed similar thermodynamic profiles in other sequences, which suggests that the mechanisms of recognition could be similar. On the other hand, the possibility that the combination of stacking and steric factors observed in the present structures could be modulated by DNA sequence must be considered. For example, one might predict a more stable stacking interaction involving *O*^6^-Bn-dG if the present 3′-neighbor base A:T base pair were to be exchanged for a 3′-neighbor C:G base pair (Figures 1 and 2). It would thus be of interest to complete a structural analysis(es) of the recognition of *O*^6^-Bn-dG by dPer in other sequences.

It seems that the simultaneous insertion of the Per base and the *O*^6^-Bn-dG lesion explains the greater volume of the crystallographic unit cell (Supplementary Table S2) as compared with the canonical DDD, and that changes in the crystal packing of the *O*^6^-Bn-dG:dPer duplex explain why attempts to phase crystallographic data by the molecular replacement method failed. The electron density for the two 5′-terminal nucleotides C^1^ and C^13^ is not visible, suggesting that these bases are disordered in the crystal. The terminal bases may be unable to fit into the lattice owing to the intercalated structure of the modified duplex.

The observation that dPer recognizes *O*^6^-Bn-dG via a stacking interaction rather than via hydrogen bonding interactions is consistent with the notion that base stacking interactions are of importance in stabilizing nucleic acid duplexes and contribute to the sequence dependence of DNA duplex stability in unmodified DNA (53–57). Inter-strand stacking interactions have been found to underlie the stability of some chemically modified DNA duplexes. For example, Gallego and Loakes (58) reported on the solution structure and dynamics of oligodeoxynucleotide duplexes containing the universal base analogs 5-nitroindole and 5-nitroindole-3-carboxamide, concluding that these base analogs exist as a mixture of two different stacking configurations. Matsuda *et al.* (59) reported that for the 2′-deoxynucleotide containing the propynylisocarbostyril base analog (dPICS), the large aromatic rings of propynylisocarbostyril (dPICS) pair in an intercalative manner within an oligodeoxynucleotide duplex. Likewise, Malyshev *et al.* (60) determined the structure of an oligodeoxynucleotide duplex containing the unnatural dMMO2-d5SICS pair and concluded that this unnatural base pair adopted a well-defined structure, with only small helical distortions. Their structure revealed that the unnatural dMMO2-d5SICS paired via partial interstrand intercalation. The intercalation of nucleoside analogs may influence behavior in polymerase-mediated DNA synthesis reactions (59,60). Biphenyl groups placed as a pair in a DNA duplex intercalate side by side as a pair between the natural base pairs and also undergo dynamic motion (61). As well, oligonucleotides composed of achiral non-nucleosidic building blocks, such as pyrene and phenanthrene, embedded in DNA lead to duplex stabilization on the basis of inter-strand stacking interactions (62–64).

The NMR analysis leads to the conclusion that the intercalative recognition mechanism for the *O*^6^-Bn-dG:dPer pair applies in solution and provides information regarding solution dynamics of the interaction. The observation that the benzyl protons of the *O*^6^-Bn-dG appear as three resonances (Figure 3) is consistent with rotation of the benzyl ring in solution on the timescale of the NMR experiment. It is possible that the ring flipping is associated with DNA breathing motions, i.e. occurs when the DNA duplex is transiently open. This dynamic behavior probably accounts for the line broadening at base pairs C^3^:G^10^ and X^4^:Y^9^ in the NMR spectrum (Figures 3 and Supplementary Figure S6). The flipping benzyl ring between Per and T^8^ is consistent with line broadening observed both for T^8^ and dPer protons. Similar flipping of the styrenyl moiety has been observed in the NMR spectrum for the *S*(61,2)-*R*-(*N*^6^-adenyl)styrene oxide adduct, when placed in DNA (65). DNA containing the *O*^6^-Bn-G:C pairing is destabilized relative to an unmodified G:C base pair (23); however, attempts to characterize the structure of the *O*^6^-Bn-G lesion in DNA were unsuccessful, as the NMR spectra showed spectral broadening, which suggested that the lesion induced conformational disorder into the duplex. However, a structural analysis of an *O*^6^-Bn-dG modified template:primer complexed with the Y-family polymerase Dpo4 revealed that *O*^6^-Bn-dG formed a wobble base pair when placed opposite dC and pseudo Watson–Crick hydrogen bonding when placed opposite dT (66).

### dPer pairs with guanine via a wobble base pairing interaction

The present results reveal formation of a wobble pair between dPer and dG, with dPer oriented in the *anti* conformation with respect to the glycosyl bond, with hydrogen bonds involving dPer and the N1 and *N*^2^ nitrogen atoms of the guanine (Figure 9). The presence of these hydrogen bonds is consistent with the NMR data, which shows that the sequential connectivity of the base imino protons from base pairs C^3^:G^10^→G^4^:Y^9^→A^5^:T^8^ is observed (Figure 5b). Moreover, the region of the spectrum showing NOEs between the base imino and amino protons (Figure 5) is consistent with the notion that G^4^ and Y^9^ form a wobble-like base pair, as there was no break in the NOE connectivity between bases, and the T^8^→Y^9^ and Y^9^→G^10^ cross-peaks were weak. Notably, the chemical shifts for the dPer protons are observed 6.6–7.4 ppm, i.e. further downfield than for the DDD-XY duplex, suggesting reduced stacking interactions (Figure 7). These downfield shifts are consistent with the positioning of the dPer ring into the major groove, as seen in Figures 8 and 10. The observation that the *T*_M_ for the DDD-GY duplex is 5°C lower than that for the DDD-XY duplex suggests that the stability imparted by this wobble interaction is lower than that from the dPer:*O*^6^-Bn-dG intercalative interaction, perhaps also due to poorer stacking interactions between dPer and the flanking bases (Figure 10), thus providing a basis for specificity. The presence of the wobble-pair interaction, however, perhaps limits the selectivity of dPer for *O*^6^-Bn-dG over dG.

## SUMMARY

The synthetic nucleoside dPer distinguishes between *O*^6^-Bn-dG and dG in this DNA sequence by an intercalative binding mode. It enables the benzyl group of *O*^6^-Bn-dG to intercalate between dPer and thymine of the 3′-neighbor A:T base pair. The binding of the benzyl group is captured in the face-to-face stack in the crystal structure but is dynamic on the NMR timescale. In contrast, dPer forms a less stable pair with dG, which is characterized by a wobble-type H-bonding interaction. The structural insight gained in this study provides information that may be applied to chemical modifications that could further stabilize dPer:*O*^6^-Bn-dG stacking interactions and/or destabilize the dPer:*O*^6^-Bn-dG wobble interaction.

## ACCESSION NUMBERS

PDB ID code for the DDD-XY duplex is 4HQI, and for the DDD-GY duplex, the PDB ID code is 2M11.

## SUPPLEMENTARY DATA

Supplementary Data are available at NAR Online: Supplementary Tables 1–5, Supplementary Figures 1–6, Supplementary References [30–31,45,59].

## FUNDING

NIH [R01 CA-108604 to S.J.S.], [R01 GM-055237 to M.E.], [R01 ES-05509 to M.P.S.]; ERC grant [260341 to S.J.S.]. Funding for NMR was supplied by NIH grants [S10 RR-05805, S10 RR-025677 and NSF Grant DBI 0922862], the latter funded by the American Recovery and Reinvestment Act of 2009 (Public Law 111-5). Vanderbilt University assisted with the purchase of in-house crystallographic and NMR instrumentation. Use of the Advanced Photon Source was supported by the U.S. Department of Energy, Office of Science, Office of Basic Energy Sciences, under Contract No. DE-AC02-06CH11357. The LS-CAT Sector 21 beamline is supported by the Michigan Economic Development Corporation and the Michigan Technology Tri-Corridor [085P1000817]. Funding for open access charge: National Institutes of Health and ERC.

*Conflict of interest statement*. None declared.

## Supplementary Material

Supplementary Data
