# Supplementary material for: Recognition of O6-benzyl-2′-deoxyguanosine by a perimidinone-derived synthetic nucleoside: a DNA interstrand stacking interaction
Source: Nucleic Acids Res. 2013 Jul 8;41(15):7566–76. doi: 10.1093/nar/gkt488 (PMC3753623; doi:10.1093/nar/gkt488)
Supplement: Supplementary Data [file supp_41_15_7566__index.html]

Recognition of O6-benzyl-2′-deoxyguanosine by a perimidinone-derived synthetic nucleoside: a DNA interstrand stacking interaction — Recognition of O6-benzyl-2′-deoxyguanosine by a perimidinone-derived synthetic nucleoside: a DNA interstrand stacking interaction — Recognition of O6-benzyl-2′-deoxyguanosine by a perimidinone-derived synthetic nucleoside: a DNA interstrand stacking interaction — Supplementary Data 

# Recognition of *O*6-benzyl-2′-deoxyguanosine by a perimidinone-derived synthetic nucleoside: a DNA interstrand stacking interaction

## Supplementary Data

files

**Files in this Data Supplement:**

- Supplementary Data - pdf file
