# Supplementary material for: Recognition of O6-benzyl-2′-deoxyguanosine by a perimidinone-derived synthetic nucleoside: a DNA interstrand stacking interaction
Source: Nucleic Acids Res. 2013 Jul 8;41(15):7566–76. doi: 10.1093/nar/gkt488 (PMC3753623; doi:10.1093/nar/gkt488)
Supplement: Supplementary Data [file supp_gkt488_nar-00771-f-2013-File002.pdf]

Recognition of *O*^{6}-Benzyl-2'-deoxyguanosine by a Perimidinone-Derived Synthetic Nucleoside: A DNA Interstrand Stacking Interaction

Supplementary Data

Ewa A. Kowal,[1] Rahul R. Lad,[2] Pradeep S. Pallan,[3] Elizabeth Dhummakupt,[1] Zdzislaw Wawrzak,[4] Martin Egli,[3] Shana Sturla[2] and Michael P. Stone[1,3*]

[1]Department of Chemistry, Center in Molecular Toxicology, Vanderbilt-Ingram Cancer Center, Center in Structural Biology, Vanderbilt University, Nashville, TN 37235

[2]ETH Zürich, Institute of Food, Nutrition and Health, CH-8092 Zürich, Switzerland

[3]Department of Biochemistry, Vanderbilt University, Nashville, TN 37232

[4]Northwestern University, Synchrotron Research Center, 9700 S Cass Ave, Argonne, IL 60439

[*]To whom correspondence should be addressed

Telephone 615-322-2589

FAX 615-322-7591

e-mail michael.p.stone@vanderbilt.edu

Table of Contents

Complete listings of references (30), (31), (45), (59) 4

Table S1, CIF File for Diffraction Data of the Second Crystal of the DDD-XY Duplex 6

Table S2, Crystal and Data Collection Statistics for the First Crystal Used to Obtain Phases for the DDD-XY Duplex 48

Table S3, Crystal Data, Data Collection, and Refinement Statistics for the Second Crystal Used to Obtain the Final Structure of the DDD-XY Duplex 49

Table S4, NMR Restraints Used for the DDD-GY Structure Calculations and Refinement Statistics 50

Table S5, Structural Statistics for the DDD-GY Duplex 51

Figure S1, Structure of the DDD-XY duplex with water molecules and Sr^{2+} ion and spermine molecule 52

Figure S2, Interbase pair parameters, helical rise, roll, and twist for the DDD-XY, DDD (PDB entry 355D) duplexes 53

Figure S3, Comparison of backbone torsion angles α and β in the crystal structures of the DDD-XY, DDD (PDB entry 355D) duplexes 54

Figure S4, Comparison of γ, δ, and ε angles in the crystal structures of the DDD-XY, DDD (PDB entry 355D) duplexes 55

Figure S5, Comparison of χ and ζ angles in the crystal structures of the DDD-XY, DDD (PDB entry 355D) duplexes 56

Figure S6, NOESY spectrum of the DDD-XY duplex showing sequential NOEs between the aromatic and anomeric protons from C^1 to G^{12} 57

Figure S7, Interstrand NOE cross peaks between complementary bases for the DDD-XY

duplex and NOE connectivity of DDD-XY duplex, for the imino protons 58

Figure S8, NMR spectra, showing the imino proton resonances for the DDD-XY duplex as a function of temperature 59

Figure S9, The average structure obtained from a series of rMD calculations for the DDD-GY duplex 60

Figure S10, The superimposed nine structures obtained from a series of rMD calculations for the DDD-GY duplex 61

Figure S11, Interbase pair parameters helical rise, roll, and twist for the DDD-GY, DDD (PDB entry 355D) duplexes 62

Figure S12, Comparison of backbone torsion angles α and β in the structures of the DDD-GY, DDD (PDB entry 355D) duplexes 63

Figure S13, Comparison of γ, δ, and ε angles in the structures of the DDD-GY, DDD (PDB entry 355D) duplexes 64

Figure S14, Comparison of χ and ζ angles in the crystal structures of the DDD-GY, DDD (PDB entry 355D) duplexes. 65

Complete Listing of Reference (30).

30. Adams, P. D.; Afonine, P. V.; Bunkoczi, G.; Chen, V. B.; Davis, I. W.; Echols, N.; Headd, J. J.; Hung, L. W.; Kapral, G. J.; Grosse-Kunstleve, R. W.; McCoy, A. J.; Moriarty, N. W.; Oeffner, R.; Read, R. J.; Richardson, D. C.; Richardson, J. S.; Terwilliger, T. C.; Zwart, P. H. *Acta Crystallogr. D* **2010**, *66*, 213-221.

Complete Listing of Reference (31).

31. Brunger, A. T.; et al. Adams, P. D.; Clore, G. M.; DeLano, W. L.; Gros, P.; Grosse-46, R. W.; Jiang, J. S.; Kuszewski, J.; Nilges, M.; Pannu, N. S.; Read, R. J.; Rice, L. M.; Simonson, T.; Warren, G. L. *Acta Crystallogr. D* **1998**, *54*, 905-921.

Complete Listing of Reference (45).

45. Frisch, M. J.; Trucks, G. W.; Schlegel, H. B.; Scuseria, G. E.; Robb, M. A.; Cheeseman, J. R.; Montgomery, J. A.; Vreven, T.; Kudin, K. N.; Burant, J. C.; Millam, J. M.; Iyengar, S. S.; Tomasi, J.; Barone, V.; Mennucci, B.; Cossi, M.; Scalmani, G.; Rega, N.; Petersson, G. A.; Nakatsuji, H.; Hada, M.; Ehara, M.; Toyota, K.; Fukuda, R.; Hasegawa, J.; Ishida, M.; Nakajima, T.; Honda, Y.; Kitao, O.; Nakai, H.; Klene, M.; Li, X.; Knox, J. E.; Hratchian, H. P.; Cross, J. B.; Adamo, C.; Jaramillo, J.; Gomperts, R.; Stratmann, R. E.; Yazyev, O.; Austin, A. J.; Cammi, R.; Pomelli, C.; Pomelli, J.; Ochterski, W.; Ayala, P. Y.; Morokuma, K.; Voth, G. A.; Salvador, P.; Dannenberg, J. J.; Zakrzewska, V. G.; Daniels, A. D.; Farkas, O.; Rabuck, A. D.; Raghavachari, K.; Ortiz, J. V. *Gaussian, Inc., Wallingford, CT* **2004**.

Complete Listing of Reference (59).

59. Matsuda, S.; Fillo, J. D.; Henry, A. A.; Rai, P.; Wilkens, S. J.; Dwyer, T. J.; Geierstanger, B. H.; Wemmer, D. E.; Schultz, P. G.; Spraggon, G.; Romesberg, F. E. *J. Am. Chem. Soc.* **2007**, *129*, 10466-10473.

Table S1. CIF File for Diffraction Data of the Second Crystal of the DDD-XY duplex.

```
data_4HQI
#
_audit_conform.dict_name       mmcif_pdbx.dic
_audit_conform.dict_version    1.0695
_audit_conform.dict_location   http://mmcif.pdb.org/dictionaries/ascii/mmcif_pdbx.dic
#
loop_
_audit_author.name
_audit_author.pdbx_ordinal
'Kowal, E.A.'  1
'Lad, R.'      2
'Pallan, P.S.' 3
'Muffly, E.'   4
'Wawrzak, Z.'  5
'Egli, M.'     6
'Sturla, S.J.' 7
'Stone, M.P.'  8
#
_pdbx_database_status.status_code      AUTH
_pdbx_database_status.entry_id         4HQI
_pdbx_database_status.deposit_site     RCSB
_pdbx_database_status.process_site     RCSB
_pdbx_database_status.status_code_sf   HPUB
_pdbx_database_status.status_code_mr   ?
_pdbx_database_status.SG_entry         ?
_pdbx_database_status.status_code_cs   ?
#
_database_PDB_rev.num              ?
_database_PDB_rev.date             ?
_database_PDB_rev.date_original    2012-10-25
_database_PDB_rev.status           ?
_database_PDB_rev.replaces         ?
_database_PDB_rev.mod_type         ?
#
_struct_ref.id                         1
```

```
_struct_ref.db_name                    PDB
_struct_ref.db_code                    4HQI
_struct_ref.pdbx_db_accession          4HQI
_struct_ref.entity_id                  1
_struct_ref.biol_id                    .
_struct_ref.pdbx_align_begin           ?
_struct_ref.pdbx_seq_one_letter_code   ?
#
loop_
_struct_ref_seq.align_id
_struct_ref_seq.ref_id
_struct_ref_seq.pdbx_PDB_id_code
_struct_ref_seq.pdbx_strand_id
_struct_ref_seq.seq_align_beg
_struct_ref_seq.pdbx_seq_align_beg_ins_code
_struct_ref_seq.seq_align_end
_struct_ref_seq.pdbx_seq_align_end_ins_code
_struct_ref_seq.pdbx_db_accession
_struct_ref_seq.db_align_beg
_struct_ref_seq.db_align_end
_struct_ref_seq.pdbx_auth_seq_align_beg
_struct_ref_seq.pdbx_auth_seq_align_end
1 1 4HQI A 1 ? 12 ? 4HQI 1  12 1  12
2 1 4HQI B 1 ? 12 ? 4HQI 13 24 13 24
#
_struct_keywords.entry_id        4HQI
_struct_keywords.pdbx_keywords   DNA
_struct_keywords.text
;B-form DNA, O6-Benzyl-2'-deoxyguanosine, dPer, Perimidinone-Derived Nucleoside, Dickerson-Drew
Dodecamer, DNA
;
#
_pdbx_struct_assembly.id                   1
_pdbx_struct_assembly.details              author_and_software_defined_assembly
_pdbx_struct_assembly.method_details       PISA
_pdbx_struct_assembly.oligomeric_details   dimeric
_pdbx_struct_assembly.oligomeric_count     2
```

```
#
_pdbx_struct_assembly_gen.assembly_id       1
_pdbx_struct_assembly_gen.oper_expression   1
_pdbx_struct_assembly_gen.asym_id_list      A,C,D,E,B,F
#
loop_
_pdbx_struct_assembly_prop.biol_id
_pdbx_struct_assembly_prop.type
_pdbx_struct_assembly_prop.value
_pdbx_struct_assembly_prop.details
1 'ABSA (A^2)' 1890 ?
1 'SSA (A^2)'  4860 ?
1 MORE         -24  ?
#
_pdbx_struct_oper_list.id                   1
_pdbx_struct_oper_list.type                 'identity operation'
_pdbx_struct_oper_list.name                 1_555
_pdbx_struct_oper_list.symmetry_operation   x,y,z
_pdbx_struct_oper_list.matrix[1][1]         1.0000000000
_pdbx_struct_oper_list.matrix[1][2]         0.0000000000
_pdbx_struct_oper_list.matrix[1][3]         0.0000000000
_pdbx_struct_oper_list.vector[1]            0.0000000000
_pdbx_struct_oper_list.matrix[2][1]         0.0000000000
_pdbx_struct_oper_list.matrix[2][2]         1.0000000000
_pdbx_struct_oper_list.matrix[2][3]         0.0000000000
_pdbx_struct_oper_list.vector[2]            0.0000000000
_pdbx_struct_oper_list.matrix[3][1]         0.0000000000
_pdbx_struct_oper_list.matrix[3][2]         0.0000000000
_pdbx_struct_oper_list.matrix[3][3]         1.0000000000
_pdbx_struct_oper_list.vector[3]            0.0000000000
#
_struct_biol.id        1
_struct_biol.details   ?
#
loop_
_exptl_crystal_grow.crystal_id
_exptl_crystal_grow.method
```

```
_exptl_crystal_grow.temp
_exptl_crystal_grow.temp_details
_exptl_crystal_grow.pH
_exptl_crystal_grow.pdbx_details
_exptl_crystal_grow.pdbx_pH_range
1 'VAPOR DIFFUSION, HANGING DROP' 291 ? 7.0
;20 mM sodium cacodylate (pH 7.0), 6 mM spermine tetra-HCl, 20 mM LiCl, 40 mM SrCl2 and  5% v/v 2-
Methyl-2,4-pentanediol (MPD), VAPOR DIFFUSION, HANGING DROP, temperature 291K
;
?
2 'VAPOR DIFFUSION, HANGING DROP' 291 ? 7.0
;20 mM sodium cacodylate (pH 7.0), 6 mM spermine tetra-HCl, 40 mM KCl, 10 mM BaCl2 and  5% v/v 2-
Methyl-2,4-pentanediol (MPD), VAPOR DIFFUSION, HANGING DROP, temperature 291K
;
?
#
loop_
_exptl_crystal.id
_exptl_crystal.density_meas
_exptl_crystal.density_Matthews
_exptl_crystal.density_percent_sol
_exptl_crystal.description
_exptl_crystal.F_000
_exptl_crystal.preparation
1 ? 2.46 50.02 ? ? ?
2 ? ?    ?     ? ? ?
#
_cell.entry_id           4HQI
_cell.length_a           26.384
_cell.length_b           36.774
_cell.length_c           77.653
_cell.angle_alpha        90.00
_cell.angle_beta         90.00
_cell.angle_gamma        90.00
_cell.Z_PDB              8
_cell.pdbx_unique_axis   ?
_cell.length_a_esd       ?
```

```
_cell.length_b_esd       ?
_cell.length_c_esd       ?
_cell.angle_alpha_esd    ?
_cell.angle_beta_esd     ?
_cell.angle_gamma_esd    ?
#
_symmetry.entry_id                          4HQI
_symmetry.space_group_name_H-M              'P 21 21 21'
_symmetry.pdbx_full_space_group_name_H-M    ?
_symmetry.cell_setting                      ?
_symmetry.Int_Tables_number                 ?
_symmetry.space_group_name_Hall             ?
#
loop_
_diffrn_source.diffrn_id
_diffrn_source.source
_diffrn_source.type
_diffrn_source.pdbx_synchrotron_site
_diffrn_source.pdbx_synchrotron_beamline
_diffrn_source.pdbx_wavelength
_diffrn_source.pdbx_wavelength_list
1 SYNCHROTRON 'APS BEAMLINE 21-ID-D' APS 21-ID-D ? 1.60499
2 SYNCHROTRON 'APS BEAMLINE 21-ID-G' APS 21-ID-G ? 0.97857
#
loop_
_diffrn_detector.diffrn_id
_diffrn_detector.detector
_diffrn_detector.type
_diffrn_detector.pdbx_collection_date
_diffrn_detector.details
1 CCD 'MARMOSAIC 300 mm CCD' 2009-07-17 Si(111)
2 CCD 'MARMOSAIC 300 mm CCD' 2008-07-18 C(111)
#
loop_
_diffrn.id
_diffrn.ambient_temp
_diffrn.ambient_temp_details
```

```
_diffrn.crystal_id
1 100 ? 1
2 100 ? 2
#
loop_
_diffrn_radiation.diffrn_id
_diffrn_radiation.wavelength_id
_diffrn_radiation.pdbx_monochromatic_or_laue_m_l
_diffrn_radiation.monochromator
_diffrn_radiation.pdbx_diffrn_protocol
1 1 M Si(111) 'SINGLE WAVELENGTH'
2 1 M C(111)  'SINGLE WAVELENGTH'
#
_reflns.entry_id                        4HQI
_reflns.observed_criterion_sigma_I      5
_reflns.observed_criterion_sigma_F      ?
_reflns.d_resolution_low                30.0
_reflns.d_resolution_high               1.70
_reflns.number_obs                      8236
_reflns.number_all                      8811
_reflns.percent_possible_obs            93.4
_reflns.pdbx_Rmerge_I_obs               0.044
_reflns.pdbx_Rsym_value                 ?
_reflns.pdbx_netI_over_sigmaI           52.33
_reflns.B_iso_Wilson_estimate           ?
_reflns.pdbx_redundancy                 6.4
_reflns.R_free_details                  ?
_reflns.limit_h_max                     ?
_reflns.limit_h_min                     ?
_reflns.limit_k_max                     ?
_reflns.limit_k_min                     ?
_reflns.limit_l_max                     ?
_reflns.limit_l_min                     ?
_reflns.observed_criterion_F_max        ?
_reflns.observed_criterion_F_min        ?
_reflns.pdbx_chi_squared                ?
_reflns.pdbx_scaling_rejects            ?
```

```
#
_reflns_shell.d_res_high              1.70
_reflns_shell.d_res_low               1.76
_reflns_shell.percent_possible_all    ?
_reflns_shell.Rmerge_I_obs            0.228
_reflns_shell.pdbx_Rsym_value         ?
_reflns_shell.meanI_over_sigI_obs     5.65
_reflns_shell.pdbx_redundancy         4
_reflns_shell.percent_possible_obs    ?
_reflns_shell.number_unique_all       524
_reflns_shell.number_measured_all     ?
_reflns_shell.number_measured_obs     ?
_reflns_shell.number_unique_obs       ?
_reflns_shell.pdbx_chi_squared        ?
#
_refine.entry_id                                4HQI
_refine.ls_number_reflns_obs                    7486
_refine.ls_number_reflns_all                    ?
_refine.pdbx_ls_sigma_I                         ?
_refine.pdbx_ls_sigma_F                         ?
_refine.pdbx_data_cutoff_high_absF              ?
_refine.pdbx_data_cutoff_low_absF               ?
_refine.pdbx_data_cutoff_high_rms_absF          ?
_refine.ls_d_res_low                            20.67
_refine.ls_d_res_high                           1.70
_refine.ls_percent_reflns_obs                   93.39
_refine.ls_R_factor_obs                         0.26227
_refine.ls_R_factor_all                         ?
_refine.ls_R_factor_R_work                      0.25885
_refine.ls_R_factor_R_free                      0.29823
_refine.ls_R_factor_R_free_error                ?
_refine.ls_R_factor_R_free_error_details        ?
_refine.ls_percent_reflns_R_free                8.9
_refine.ls_number_reflns_R_free                 734
_refine.ls_number_parameters                    ?
_refine.ls_number_restraints                    ?
_refine.occupancy_min                           ?
```

```
_refine.occupancy_max                          ?
_refine.correlation_coeff_Fo_to_Fc             0.954
_refine.correlation_coeff_Fo_to_Fc_free        0.940
_refine.B_iso_mean                             44.140
_refine.aniso_B[1][1]                          3.33
_refine.aniso_B[2][2]                          1.38
_refine.aniso_B[3][3]                          -4.71
_refine.aniso_B[1][2]                          -0.00
_refine.aniso_B[1][3]                          -0.00
_refine.aniso_B[2][3]                          0.00
_refine.solvent_model_details                  MASK
_refine.solvent_model_param_ksol               ?
_refine.solvent_model_param_bsol               ?
_refine.pdbx_solvent_vdw_probe_radii           1.20
_refine.pdbx_solvent_ion_probe_radii           0.80
_refine.pdbx_solvent_shrinkage_radii           0.80
_refine.pdbx_ls_cross_valid_method             THROUGHOUT
_refine.details                                ?
_refine.pdbx_starting_model                    ?
_refine.pdbx_method_to_determine_struct        SAD
_refine.pdbx_isotropic_thermal_model           ?
_refine.pdbx_stereochemistry_target_values     'MAXIMUM LIKELIHOOD'
_refine.pdbx_stereochem_target_val_spec_case   ?
_refine.pdbx_R_Free_selection_details          RANDOM
_refine.pdbx_overall_ESU_R                     0.149
_refine.pdbx_overall_ESU_R_Free                0.144
_refine.overall_SU_ML                          0.130
_refine.pdbx_overall_phase_error               ?
_refine.overall_SU_B                           4.224
_refine.overall_SU_R_Cruickshank_DPI           ?
_refine.ls_redundancy_reflns_obs               ?
_refine.B_iso_min                              ?
_refine.B_iso_max                              ?
_refine.overall_SU_R_free                      ?
_refine.ls_wR_factor_R_free                    ?
_refine.ls_wR_factor_R_work                    ?
_refine.overall_FOM_free_R_set                 ?
```

```
_refine.overall_FOM_work_R_set                   ?
_refine.pdbx_refine_id                           'X-RAY DIFFRACTION'
#
loop_
_refine_ls_restr.type
_refine_ls_restr.dev_ideal
_refine_ls_restr.dev_ideal_target
_refine_ls_restr.weight
_refine_ls_restr.number
_refine_ls_restr.pdbx_restraint_function
_refine_ls_restr.pdbx_refine_id
r_bond_refined_d     0.011 0.013 ? 554 ? 'X-RAY DIFFRACTION'
r_angle_refined_deg  1.652 1.578 ? 833 ? 'X-RAY DIFFRACTION'
r_chiral_restr       0.125 0.200 ? 66  ? 'X-RAY DIFFRACTION'
r_gen_planes_refined 0.023 0.020 ? 260 ? 'X-RAY DIFFRACTION'
#
_refine_ls_shell.pdbx_total_number_of_bins_used   20
_refine_ls_shell.d_res_high                       1.70
_refine_ls_shell.d_res_low                        1.745
_refine_ls_shell.number_reflns_R_work             338
_refine_ls_shell.R_factor_R_work                  0.336
_refine_ls_shell.percent_reflns_obs               57.01
_refine_ls_shell.R_factor_R_free                  0.336
_refine_ls_shell.R_factor_R_free_error            ?
_refine_ls_shell.percent_reflns_R_free            ?
_refine_ls_shell.number_reflns_R_free             36
_refine_ls_shell.number_reflns_all                ?
_refine_ls_shell.R_factor_all                     ?
_refine_ls_shell.number_reflns_obs                ?
_refine_ls_shell.redundancy_reflns_obs            ?
_refine_ls_shell.pdbx_refine_id                   'X-RAY DIFFRACTION'
#
_database_PDB_matrix.entry_id          4HQI
_database_PDB_matrix.origx[1][1]       1.000000
_database_PDB_matrix.origx[1][2]       0.000000
_database_PDB_matrix.origx[1][3]       0.000000
_database_PDB_matrix.origx[2][1]       0.000000
```

```
_database_PDB_matrix.origx[2][2]       1.000000
_database_PDB_matrix.origx[2][3]       0.000000
_database_PDB_matrix.origx[3][1]       0.000000
_database_PDB_matrix.origx[3][2]       0.000000
_database_PDB_matrix.origx[3][3]       1.000000
_database_PDB_matrix.origx_vector[1]   0.00000
_database_PDB_matrix.origx_vector[2]   0.00000
_database_PDB_matrix.origx_vector[3]   0.00000
#
_exptl.entry_id          4HQI
_exptl.method            'X-RAY DIFFRACTION'
_exptl.crystals_number   2
#
loop_
_ndb_struct_conf_na.entry_id
_ndb_struct_conf_na.feature
4HQI 'b-form double helix'
4HQI 'mismatched base pair'
#
loop_
_ndb_struct_na_base_pair.model_number
_ndb_struct_na_base_pair.i_label_asym_id
_ndb_struct_na_base_pair.i_label_comp_id
_ndb_struct_na_base_pair.i_label_seq_id
_ndb_struct_na_base_pair.i_symmetry
_ndb_struct_na_base_pair.j_label_asym_id
_ndb_struct_na_base_pair.j_label_comp_id
_ndb_struct_na_base_pair.j_label_seq_id
_ndb_struct_na_base_pair.j_symmetry
_ndb_struct_na_base_pair.shear
_ndb_struct_na_base_pair.stretch
_ndb_struct_na_base_pair.stagger
_ndb_struct_na_base_pair.buckle
_ndb_struct_na_base_pair.opening
_ndb_struct_na_base_pair.pair_number
_ndb_struct_na_base_pair.pair_name
_ndb_struct_na_base_pair.i_auth_asym_id
```

```
_ndb_struct_na_base_pair.i_auth_seq_id
_ndb_struct_na_base_pair.i_PDB_ins_code
_ndb_struct_na_base_pair.j_auth_asym_id
_ndb_struct_na_base_pair.j_auth_seq_id
_ndb_struct_na_base_pair.j_PDB_ins_code
_ndb_struct_na_base_pair.hbond_type_28
_ndb_struct_na_base_pair.hbond_type_12
1 A DG 2  1_555 B DC 11 1_555 -0.125 -0.253 -0.220 -11.324 -1.868 1 A_DG2:DC23_B  A 2  ? B 23 ? 19 1
1 A DC 3  1_555 B DG 10 1_555 0.199  -0.225 -0.317 2.151   -1.099 2 A_DC3:DG22_B  A 3  ? B 22 ? 19 1
1 A DA 5  1_555 B DT 8  1_555 -0.236 -0.008 -0.460 -12.406 1.251  3 A_DA5:DT20_B  A 5  ? B 20 ? 20 1
1 A DA 6  1_555 B DT 7  1_555 -0.009 -0.142 -0.125 -5.111  0.172  4 A_DA6:DT19_B  A 6  ? B 19 ? 20 1
1 A DT 7  1_555 B DA 6  1_555 0.132  -0.121 -0.126 2.516   1.351  5 A_DT7:DA18_B  A 7  ? B 18 ? 20 1
1 A DT 8  1_555 B DA 5  1_555 0.024  -0.181 -0.510 8.065   4.832  6 A_DT8:DA17_B  A 8  ? B 17 ? 20 1
1 A DG 10 1_555 B DC 3  1_555 -0.259 -0.158 -0.143 -0.234  0.472  7 A_DG10:DC15_B A 10 ? B 15 ? 19 1
1 A DC 11 1_555 B DG 2  1_555 0.194  -0.224 -0.063 10.713  -1.590 8 A_DC11:DG14_B A 11 ? B 14 ? 19 1
#
loop_
_ndb_struct_na_base_pair_step.model_number
_ndb_struct_na_base_pair_step.i_label_asym_id_1
_ndb_struct_na_base_pair_step.i_label_comp_id_1
_ndb_struct_na_base_pair_step.i_label_seq_id_1
_ndb_struct_na_base_pair_step.i_symmetry_1
_ndb_struct_na_base_pair_step.j_label_asym_id_1
_ndb_struct_na_base_pair_step.j_label_comp_id_1
_ndb_struct_na_base_pair_step.j_label_seq_id_1
_ndb_struct_na_base_pair_step.j_symmetry_1
_ndb_struct_na_base_pair_step.i_label_asym_id_2
_ndb_struct_na_base_pair_step.i_label_comp_id_2
_ndb_struct_na_base_pair_step.i_label_seq_id_2
_ndb_struct_na_base_pair_step.i_symmetry_2
_ndb_struct_na_base_pair_step.j_label_asym_id_2
_ndb_struct_na_base_pair_step.j_label_comp_id_2
_ndb_struct_na_base_pair_step.j_label_seq_id_2
_ndb_struct_na_base_pair_step.j_symmetry_2
_ndb_struct_na_base_pair_step.shift
_ndb_struct_na_base_pair_step.slide
_ndb_struct_na_base_pair_step.rise
```

```
_ndb_struct_na_base_pair_step.tilt
_ndb_struct_na_base_pair_step.roll
_ndb_struct_na_base_pair_step.twist
_ndb_struct_na_base_pair_step.x_displacement
_ndb_struct_na_base_pair_step.y_displacement
_ndb_struct_na_base_pair_step.helical_rise
_ndb_struct_na_base_pair_step.inclination
_ndb_struct_na_base_pair_step.tip
_ndb_struct_na_base_pair_step.helical_twist
_ndb_struct_na_base_pair_step.step_number
_ndb_struct_na_base_pair_step.step_name
_ndb_struct_na_base_pair_step.i_auth_asym_id_1
_ndb_struct_na_base_pair_step.i_auth_seq_id_1
_ndb_struct_na_base_pair_step.i_PDB_ins_code_1
_ndb_struct_na_base_pair_step.j_auth_asym_id_1
_ndb_struct_na_base_pair_step.j_auth_seq_id_1
_ndb_struct_na_base_pair_step.j_PDB_ins_code_1
_ndb_struct_na_base_pair_step.i_auth_asym_id_2
_ndb_struct_na_base_pair_step.i_auth_seq_id_2
_ndb_struct_na_base_pair_step.i_PDB_ins_code_2
_ndb_struct_na_base_pair_step.j_auth_asym_id_2
_ndb_struct_na_base_pair_step.j_auth_seq_id_2
_ndb_struct_na_base_pair_step.j_PDB_ins_code_2
1 A DG 2  1_555 B DC 11 1_555 A DC 3  1_555 B DG 10 1_555 0.825  -0.036 3.016 -1.267 -2.600 30.408
0.405  -1.796 2.973 -4.943 2.408   30.542 1
AA_DG2DC3:DG22DC23_BB   A 2  ? B 23 ? A 3  ? B 22 ?
1 A DA 5  1_555 B DT 8  1_555 A DA 6  1_555 B DT 7  1_555 -0.057 -0.095 3.259 -2.570 8.650  26.842 -
2.189 -0.476 3.072 18.000 5.348   28.292 2
AA_DA5DA6:DT19DT20_BB   A 5  ? B 20 ? A 6  ? B 19 ?
1 A DA 6  1_555 B DT 7  1_555 A DT 7  1_555 B DA 6  1_555 -0.148 -0.601 3.135 0.082  6.206  27.711 -
2.567 0.320  2.933 12.755 -0.169  28.384 3
AA_DA6DT7:DA18DT19_BB   A 6  ? B 19 ? A 7  ? B 18 ?
1 A DT 7  1_555 B DA 6  1_555 A DT 8  1_555 B DA 5  1_555 1.209  0.289  3.235 6.047  7.993  32.969 -
0.800 -1.070 3.374 13.696 -10.360 34.418 4
AA_DT7DT8:DA17DA18_BB   A 7  ? B 18 ? A 8  ? B 17 ?
1 A DG 10 1_555 B DC 3  1_555 A DC 11 1_555 B DG 2  1_555 -0.894 0.014  3.141 0.568  -2.918 30.844
0.565  1.778  3.110 -5.470 -1.064  30.984 5
```

```
AA_DG10DC11:DG14DC15_BB A 10 ? B 15 ? A 11 ? B 14 ?
#
loop_
_software.name
_software.classification
_software.version
_software.citation_id
_software.pdbx_ordinal
MD-2   'data collection' 'diffractometer software from EMBL (with LS-CAT developed extensions)' ? 1
PHENIX 'model building'  .                                                                      ? 2
REFMAC refinement        5.7.0029                                                               ? 3
#
_entry.id   4HQI
#
_pdbx_unobs_or_zero_occ_residues.id               1
_pdbx_unobs_or_zero_occ_residues.polymer_flag     Y
_pdbx_unobs_or_zero_occ_residues.occupancy_flag   1
_pdbx_unobs_or_zero_occ_residues.PDB_model_num    1
_pdbx_unobs_or_zero_occ_residues.auth_asym_id     A
_pdbx_unobs_or_zero_occ_residues.auth_comp_id     DC
_pdbx_unobs_or_zero_occ_residues.auth_seq_id      1
_pdbx_unobs_or_zero_occ_residues.PDB_ins_code     ?
#
loop_
_pdbx_unobs_or_zero_occ_atoms.id
_pdbx_unobs_or_zero_occ_atoms.polymer_flag
_pdbx_unobs_or_zero_occ_atoms.occupancy_flag
_pdbx_unobs_or_zero_occ_atoms.PDB_model_num
_pdbx_unobs_or_zero_occ_atoms.auth_asym_id
_pdbx_unobs_or_zero_occ_atoms.auth_comp_id
_pdbx_unobs_or_zero_occ_atoms.auth_seq_id
_pdbx_unobs_or_zero_occ_atoms.PDB_ins_code
_pdbx_unobs_or_zero_occ_atoms.auth_atom_id
_pdbx_unobs_or_zero_occ_atoms.label_alt_id
1  Y 1 1 B DC 13 ? 'O5'' ?
2  Y 1 1 B DC 13 ? 'C5'' ?
3  Y 1 1 B DC 13 ? 'C4'' ?
```

```
4  Y 1 1 B DC 13 ? 'O4'' ?
5  Y 1 1 B DC 13 ? 'C3'' ?
6  Y 1 1 B DC 13 ? 'C2'' ?
7  Y 1 1 B DC 13 ? 'C1'' ?
8  Y 1 1 B DC 13 ? N1    ?
9  Y 1 1 B DC 13 ? C2    ?
10 Y 1 1 B DC 13 ? O2    ?
11 Y 1 1 B DC 13 ? N3    ?
12 Y 1 1 B DC 13 ? C4    ?
13 Y 1 1 B DC 13 ? N4    ?
14 Y 1 1 B DC 13 ? C5    ?
15 Y 1 1 B DC 13 ? C6    ?
#
loop_
_struct_site.id
_struct_site.details
_struct_site.pdbx_evidence_code
AC1 'BINDING SITE FOR RESIDUE SPM A 101' SOFTWARE
AC2 'BINDING SITE FOR RESIDUE SR A 102'  SOFTWARE
#
_atom_sites.entry_id                    4HQI
_atom_sites.Cartn_transform_axes        ?
_atom_sites.fract_transf_matrix[1][1]   0.037902
_atom_sites.fract_transf_matrix[1][2]   0.000000
_atom_sites.fract_transf_matrix[1][3]   0.000000
_atom_sites.fract_transf_matrix[2][1]   -0.000000
_atom_sites.fract_transf_matrix[2][2]   0.027193
_atom_sites.fract_transf_matrix[2][3]   0.000000
_atom_sites.fract_transf_matrix[3][1]   0.000000
_atom_sites.fract_transf_matrix[3][2]   -0.000000
_atom_sites.fract_transf_matrix[3][3]   0.012878
_atom_sites.fract_transf_vector[1]      0.00000
_atom_sites.fract_transf_vector[2]      0.00000
_atom_sites.fract_transf_vector[3]      0.00000
#
_citation.id                        primary
_citation.title
```

```
;Recognition of O6-Benzyl-2'-deoxyguanosine by a Perimidinone-Derived Synthetic Nucleoside: A Unique
Interstrand Stacking Interaction
;
_citation.journal_abbrev            'To be Published'
_citation.journal_volume            ?
_citation.page_first                ?
_citation.page_last                 ?
_citation.year                      ?
_citation.journal_id_ASTM           ?
_citation.country                   ?
_citation.journal_id_ISSN           ?
_citation.journal_id_CSD            0353
_citation.book_publisher            ?
_citation.pdbx_database_id_PubMed   ?
_citation.pdbx_database_id_DOI      ?
#
loop_
_citation_author.citation_id
_citation_author.name
_citation_author.ordinal
primary 'Kowal, E.A.'  1
primary 'Lad, R.'      2
primary 'Pallan, P.S.' 3
primary 'Muffly, E.'   4
primary 'Wawrzak, Z.'  5
primary 'Egli, M.'     6
primary 'Sturla, S.J.' 7
primary 'Stone, M.P.'  8
#
_computing.entry_id                           4HQI
_computing.pdbx_data_reduction_ii             HKL2000
_computing.pdbx_data_reduction_ds             HKL2000
_computing.data_collection                    'MD-2 diffractometer software from EMBL (with LS-CAT
developed extensions)'
_computing.structure_solution                 PHENIX
_computing.structure_refinement               'REFMAC 5.7.0029'
_computing.pdbx_structure_refinement_method   ?
```

```
#
_database.entry_id   4HQI
_database.code_CSD   ?
#
loop_
_database_2.database_id
_database_2.database_code
PDB  4HQI
NDB  NA2104
RCSB RCSB075793
#
loop_
_entity.id
_entity.type
_entity.src_method
_entity.pdbx_description
_entity.formula_weight
_entity.pdbx_number_of_molecules
_entity.details
1 polymer     syn 'Short modified nucleic acids' 3826.640 2  ?
2 non-polymer syn SPERMINE                       202.342  1  ?
3 non-polymer syn 'STRONTIUM ION'                87.620   1  ?
4 water       nat water                          18.015   49 ?
#
loop_
_entity_poly_seq.entity_id
_entity_poly_seq.num
_entity_poly_seq.mon_id
_entity_poly_seq.hetero
1 1  DC  n
1 2  DG  n
1 3  DC  n
1 4  BZG n
1 5  DA  n
1 6  DA  n
1 7  DT  n
1 8  DT  n
```

```
1 9  D3N n
1 10 DG  n
1 11 DC  n
1 12 DG  n
#
_entity_poly.entity_id                      1
_entity_poly.type                           polydeoxyribonucleotide
_entity_poly.nstd_linkage                   no
_entity_poly.nstd_monomer                   yes
_entity_poly.pdbx_seq_one_letter_code       (DC)(DG)(DC)(BZG)(DA)(DA)(DT)(DT)(D3N)(DG)(DC)(DG)
_entity_poly.pdbx_seq_one_letter_code_can   CGCNAATTXGCG
#
loop_
_pdbx_poly_seq_scheme.asym_id
_pdbx_poly_seq_scheme.entity_id
_pdbx_poly_seq_scheme.seq_id
_pdbx_poly_seq_scheme.mon_id
_pdbx_poly_seq_scheme.ndb_seq_num
_pdbx_poly_seq_scheme.pdb_seq_num
_pdbx_poly_seq_scheme.auth_seq_num
_pdbx_poly_seq_scheme.pdb_mon_id
_pdbx_poly_seq_scheme.auth_mon_id
_pdbx_poly_seq_scheme.pdb_strand_id
_pdbx_poly_seq_scheme.pdb_ins_code
_pdbx_poly_seq_scheme.hetero
A 1 1  DC  1  1  ?  ?   ?   A . n
A 1 2  DG  2  2  2  DG  DG  A . n
A 1 3  DC  3  3  3  DC  DC  A . n
A 1 4  BZG 4  4  4  BZG BZG A . n
A 1 5  DA  5  5  5  DA  DA  A . n
A 1 6  DA  6  6  6  DA  DA  A . n
A 1 7  DT  7  7  7  DT  DT  A . n
A 1 8  DT  8  8  8  DT  DT  A . n
A 1 9  D3N 9  9  9  D3N D3N A . n
A 1 10 DG  10 10 10 DG  DG  A . n
A 1 11 DC  11 11 11 DC  DC  A . n
A 1 12 DG  12 12 12 DG  DG  A . n
```

```
B 1 1  DC  1  13 13 DC  DC  B . n
B 1 2  DG  2  14 14 DG  DG  B . n
B 1 3  DC  3  15 15 DC  DC  B . n
B 1 4  BZG 4  16 16 BZG BZG B . n
B 1 5  DA  5  17 17 DA  DA  B . n
B 1 6  DA  6  18 18 DA  DA  B . n
B 1 7  DT  7  19 19 DT  DT  B . n
B 1 8  DT  8  20 20 DT  DT  B . n
B 1 9  D3N 9  21 21 D3N D3N B . n
B 1 10 DG  10 22 22 DG  DG  B . n
B 1 11 DC  11 23 23 DC  DC  B . n
B 1 12 DG  12 24 24 DG  DG  B . n
#
loop_
_pdbx_nonpoly_scheme.asym_id
_pdbx_nonpoly_scheme.entity_id
_pdbx_nonpoly_scheme.mon_id
_pdbx_nonpoly_scheme.ndb_seq_num
_pdbx_nonpoly_scheme.pdb_seq_num
_pdbx_nonpoly_scheme.auth_seq_num
_pdbx_nonpoly_scheme.pdb_mon_id
_pdbx_nonpoly_scheme.auth_mon_id
_pdbx_nonpoly_scheme.pdb_strand_id
_pdbx_nonpoly_scheme.pdb_ins_code
C 2 SPM 1  101 330 SPM SPM A .
D 3 SR  1  102 1   SR  SR  A .
E 4 HOH 1  201 4   HOH HOH A .
E 4 HOH 2  202 7   HOH HOH A .
E 4 HOH 3  203 11  HOH HOH A .
E 4 HOH 4  204 12  HOH HOH A .
E 4 HOH 5  205 13  HOH HOH A .
E 4 HOH 6  206 19  HOH HOH A .
E 4 HOH 7  207 20  HOH HOH A .
E 4 HOH 8  208 21  HOH HOH A .
E 4 HOH 9  209 22  HOH HOH A .
E 4 HOH 10 210 23  HOH HOH A .
E 4 HOH 11 211 24  HOH HOH A .
```

```
E 4 HOH 12 212 25  HOH HOH A .
E 4 HOH 13 213 26  HOH HOH A .
E 4 HOH 14 214 28  HOH HOH A .
E 4 HOH 15 215 30  HOH HOH A .
E 4 HOH 16 216 31  HOH HOH A .
E 4 HOH 17 217 32  HOH HOH A .
E 4 HOH 18 218 33  HOH HOH A .
E 4 HOH 19 219 34  HOH HOH A .
E 4 HOH 20 220 35  HOH HOH A .
E 4 HOH 21 221 37  HOH HOH A .
E 4 HOH 22 222 38  HOH HOH A .
E 4 HOH 23 223 39  HOH HOH A .
E 4 HOH 24 224 41  HOH HOH A .
E 4 HOH 25 225 44  HOH HOH A .
E 4 HOH 26 226 48  HOH HOH A .
E 4 HOH 27 227 49  HOH HOH A .
E 4 HOH 28 228 50  HOH HOH A .
F 4 HOH 1  101 2   HOH HOH B .
F 4 HOH 2  102 3   HOH HOH B .
F 4 HOH 3  103 5   HOH HOH B .
F 4 HOH 4  104 6   HOH HOH B .
F 4 HOH 5  105 8   HOH HOH B .
F 4 HOH 6  106 9   HOH HOH B .
F 4 HOH 7  107 10  HOH HOH B .
F 4 HOH 8  108 14  HOH HOH B .
F 4 HOH 9  109 15  HOH HOH B .
F 4 HOH 10 110 16  HOH HOH B .
F 4 HOH 11 111 17  HOH HOH B .
F 4 HOH 12 112 18  HOH HOH B .
F 4 HOH 13 113 27  HOH HOH B .
F 4 HOH 14 114 29  HOH HOH B .
F 4 HOH 15 115 36  HOH HOH B .
F 4 HOH 16 116 40  HOH HOH B .
F 4 HOH 17 117 42  HOH HOH B .
F 4 HOH 18 118 43  HOH HOH B .
F 4 HOH 19 119 45  HOH HOH B .
F 4 HOH 20 120 46  HOH HOH B .
```

```
F 4 HOH 21 121 47  HOH HOH B .
#
_pdbx_entity_src_syn.entity_id              1
_pdbx_entity_src_syn.organism_scientific    'SYNTHETIC CONSTRUCT'
_pdbx_entity_src_syn.organism_common_name   ?
_pdbx_entity_src_syn.ncbi_taxonomy_id       32630
_pdbx_entity_src_syn.details                'Chemically synthesized modified oligonucleotides'
#
_refine_hist.pdbx_refine_id                  'X-RAY DIFFRACTION'
_refine_hist.cycle_id                        LAST
_refine_hist.pdbx_number_atoms_protein       0
_refine_hist.pdbx_number_atoms_nucleic_acid  481
_refine_hist.pdbx_number_atoms_ligand        15
_refine_hist.number_atoms_solvent            49
_refine_hist.number_atoms_total              545
_refine_hist.d_res_high                      1.70
_refine_hist.d_res_low                       20.67
#
_struct.entry_id                  4HQI
_struct.title
;Structure of O6-Benzyl-2'-deoxyguanosine opposite perimidinone-derived synthetic nucleoside in DNA
duplex
;
_struct.pdbx_descriptor           '5'-D(*CP*GP*CP*(BZG)P*AP*AP*TP*TP*(D3N)P*GP*CP*G)-3''
_struct.pdbx_model_details        ?
_struct.pdbx_CASP_flag            ?
_struct.pdbx_model_type_details   ?
#
loop_
_struct_asym.id
_struct_asym.pdbx_blank_PDB_chainid_flag
_struct_asym.pdbx_modified
_struct_asym.entity_id
_struct_asym.details
A N N 1 ?
B N N 1 ?
C N N 2 ?
```

```
D N N 3 ?
E N N 4 ?
F N N 4 ?
#
loop_
_struct_conn.id
_struct_conn.conn_type_id
_struct_conn.pdbx_PDB_id
_struct_conn.ptnr1_label_asym_id
_struct_conn.ptnr1_label_comp_id
_struct_conn.ptnr1_label_seq_id
_struct_conn.ptnr1_label_atom_id
_struct_conn.pdbx_ptnr1_label_alt_id
_struct_conn.pdbx_ptnr1_PDB_ins_code
_struct_conn.pdbx_ptnr1_standard_comp_id
_struct_conn.ptnr1_symmetry
_struct_conn.ptnr2_label_asym_id
_struct_conn.ptnr2_label_comp_id
_struct_conn.ptnr2_label_seq_id
_struct_conn.ptnr2_label_atom_id
_struct_conn.pdbx_ptnr2_label_alt_id
_struct_conn.pdbx_ptnr2_PDB_ins_code
_struct_conn.ptnr1_auth_asym_id
_struct_conn.ptnr1_auth_comp_id
_struct_conn.ptnr1_auth_seq_id
_struct_conn.ptnr2_auth_asym_id
_struct_conn.ptnr2_auth_comp_id
_struct_conn.ptnr2_auth_seq_id
_struct_conn.ptnr2_symmetry
_struct_conn.pdbx_ptnr3_label_atom_id
_struct_conn.pdbx_ptnr3_label_seq_id
_struct_conn.pdbx_ptnr3_label_comp_id
_struct_conn.pdbx_ptnr3_label_asym_id
_struct_conn.pdbx_ptnr3_label_alt_id
_struct_conn.pdbx_ptnr3_PDB_ins_code
_struct_conn.details
_struct_conn.pdbx_dist_value
```

```
_struct_conn.pdbx_value_order
metalc1  metalc ? A DG 12 O6    ? ? ? 1_555 D SR  .  SR ? ? A DG 12 A SR  102 1_555 ? ? ? ? ? ? ?
2.690 ?
covale1  covale ? A DT 8  'O3'' ? ? ? 1_555 A D3N 9  P  ? ? A DT 8  A D3N 9   1_555 ? ? ? ? ? ? ?
1.578 ?
covale2  covale ? B DT 8  'O3'' ? ? ? 1_555 B D3N 9  P  ? ? B DT 20 B D3N 21  1_555 ? ? ? ? ? ? ?
1.591 ?
covale3  covale ? A DC 3  'O3'' ? ? ? 1_555 A BZG 4  P  ? ? A DC 3  A BZG 4   1_555 ? ? ? ? ? ? ?
1.586 ?
covale4  covale ? B DC 3  'O3'' ? ? ? 1_555 B BZG 4  P  ? ? B DC 15 B BZG 16  1_555 ? ? ? ? ? ? ?
1.595 ?
hydrog1  hydrog ? A DG 2  N1    ? ? ? 1_555 B DC  11 N3 ? ? A DG 2  B DC  23  1_555 ? ? ? ? ? ?
WATSON-CRICK ?     ?
hydrog2  hydrog ? A DG 2  N2    ? ? ? 1_555 B DC  11 O2 ? ? A DG 2  B DC  23  1_555 ? ? ? ? ? ?
WATSON-CRICK ?     ?
hydrog3  hydrog ? A DG 2  O6    ? ? ? 1_555 B DC  11 N4 ? ? A DG 2  B DC  23  1_555 ? ? ? ? ? ?
WATSON-CRICK ?     ?
hydrog4  hydrog ? A DC 3  N3    ? ? ? 1_555 B DG  10 N1 ? ? A DC 3  B DG  22  1_555 ? ? ? ? ? ?
WATSON-CRICK ?     ?
hydrog5  hydrog ? A DC 3  N4    ? ? ? 1_555 B DG  10 O6 ? ? A DC 3  B DG  22  1_555 ? ? ? ? ? ?
WATSON-CRICK ?     ?
hydrog6  hydrog ? A DC 3  O2    ? ? ? 1_555 B DG  10 N2 ? ? A DC 3  B DG  22  1_555 ? ? ? ? ? ?
WATSON-CRICK ?     ?
hydrog7  hydrog ? A DA 5  N1    ? ? ? 1_555 B DT  8  N3 ? ? A DA 5  B DT  20  1_555 ? ? ? ? ? ?
WATSON-CRICK ?     ?
hydrog8  hydrog ? A DA 5  N6    ? ? ? 1_555 B DT  8  O4 ? ? A DA 5  B DT  20  1_555 ? ? ? ? ? ?
WATSON-CRICK ?     ?
hydrog9  hydrog ? A DA 6  N1    ? ? ? 1_555 B DT  7  N3 ? ? A DA 6  B DT  19  1_555 ? ? ? ? ? ?
WATSON-CRICK ?     ?
hydrog10 hydrog ? A DA 6  N6    ? ? ? 1_555 B DT  7  O4 ? ? A DA 6  B DT  19  1_555 ? ? ? ? ? ?
WATSON-CRICK ?     ?
hydrog11 hydrog ? A DT 7  N3    ? ? ? 1_555 B DA  6  N1 ? ? A DT 7  B DA  18  1_555 ? ? ? ? ? ?
WATSON-CRICK ?     ?
hydrog12 hydrog ? A DT 7  O4    ? ? ? 1_555 B DA  6  N6 ? ? A DT 7  B DA  18  1_555 ? ? ? ? ? ?
WATSON-CRICK ?     ?
hydrog13 hydrog ? A DT 8  N3    ? ? ? 1_555 B DA  5  N1 ? ? A DT 8  B DA  17  1_555 ? ? ? ? ? ?
WATSON-CRICK ?     ?
```

```
hydrog14 hydrog ? A DT 8  O4    ? ? ? 1_555 B DA  5  N6 ? ? A DT 8  B DA  17  1_555 ? ? ? ? ? ?
WATSON-CRICK ?     ?
hydrog15 hydrog ? A DG 10 N1    ? ? ? 1_555 B DC  3  N3 ? ? A DG 10 B DC  15  1_555 ? ? ? ? ? ?
WATSON-CRICK ?     ?
hydrog16 hydrog ? A DG 10 N2    ? ? ? 1_555 B DC  3  O2 ? ? A DG 10 B DC  15  1_555 ? ? ? ? ? ?
WATSON-CRICK ?     ?
hydrog17 hydrog ? A DG 10 O6    ? ? ? 1_555 B DC  3  N4 ? ? A DG 10 B DC  15  1_555 ? ? ? ? ? ?
WATSON-CRICK ?     ?
hydrog18 hydrog ? A DC 11 N3    ? ? ? 1_555 B DG  2  N1 ? ? A DC 11 B DG  14  1_555 ? ? ? ? ? ?
WATSON-CRICK ?     ?
hydrog19 hydrog ? A DC 11 N4    ? ? ? 1_555 B DG  2  O6 ? ? A DC 11 B DG  14  1_555 ? ? ? ? ? ?
WATSON-CRICK ?     ?
hydrog20 hydrog ? A DC 11 O2    ? ? ? 1_555 B DG  2  N2 ? ? A DC 11 B DG  14  1_555 ? ? ? ? ? ?
WATSON-CRICK ?     ?
#
loop_
_struct_conn_type.id
_struct_conn_type.criteria
_struct_conn_type.reference
covale ?
?
metalc ?
?
hydrog 'For hydrogen bonding between nucleic acid bases, donor to acceptor distance of 2.2 -3.5
Angstroms was used.' ?
#
loop_
_struct_site_gen.id
_struct_site_gen.site_id
_struct_site_gen.pdbx_num_res
_struct_site_gen.label_comp_id
_struct_site_gen.label_asym_id
_struct_site_gen.label_seq_id
_struct_site_gen.pdbx_auth_ins_code
_struct_site_gen.auth_comp_id
_struct_site_gen.auth_asym_id
_struct_site_gen.auth_seq_id
```

```
_struct_site_gen.label_atom_id
_struct_site_gen.label_alt_id
_struct_site_gen.symmetry
_struct_site_gen.details
1 AC1 6 DG  A 2  ? DG  A 2  . . 1_555 ?
2 AC1 6 D3N A 9  ? D3N A 9  . . 2_555 ?
3 AC1 6 D3N A 9  ? D3N A 9  . . 3_645 ?
4 AC1 6 DG  A 10 ? DG  A 10 . . 2_555 ?
5 AC1 6 DG  B 2  ? DG  B 14 . . 2_555 ?
6 AC1 6 DG  B 10 ? DG  B 22 . . 1_555 ?
7 AC2 1 DG  A 12 ? DG  A 12 . . 1_555 ?
#
loop_
_diffrn_radiation_wavelength.id
_diffrn_radiation_wavelength.wavelength
_diffrn_radiation_wavelength.wt
1 1.60499 1.0
2 0.97857 1.0
#
loop_
_atom_type.symbol
P
O
C
N
SR
#
loop_
_chem_comp.id
_chem_comp.type
_chem_comp.mon_nstd_flag
_chem_comp.name
_chem_comp.pdbx_synonyms
_chem_comp.formula
_chem_comp.formula_weight
DG  'DNA linking' y '2'-DEOXYGUANOSINE-5'-MONOPHOSPHATE'
?
```

```
'C10 H14 N5 O7 P' 347.224
DC  'DNA linking' y '2'-DEOXYCYTIDINE-5'-MONOPHOSPHATE'
?
'C9 H14 N3 O7 P'  307.199
BZG 'DNA linking' . 6-(BENZYLOXY)-9-(2-DEOXY-5-O-PHOSPHONO-BETA-D-ERYTHRO-PENTOFURANOSYL)-9H-PURIN-2-
AMINE 'O6-BENZYL-2'-DEOXYGUANOSINE-5'-MONOPHOSPHATE'
'C17 H20 N5 O7 P' 437.348
DA  'DNA linking' y '2'-DEOXYADENOSINE-5'-MONOPHOSPHATE'
?
'C10 H14 N5 O6 P' 331.224
DT  'DNA linking' y 'THYMIDINE-5'-MONOPHOSPHATE'
?
'C10 H15 N2 O8 P' 322.211
D3N 'DNA linking' . 1-(2-DEOXY-5-O-PHOSPHONO-BETA-D-ERYTHRO-PENTOFURANOSYL)-1H-PERIMIDIN-2(3H)-ONE
?
'C16 H17 N2 O7 P' 380.293
SPM NON-POLYMER   . SPERMINE
?
'C10 H26 N4'      202.342
SR  NON-POLYMER   . 'STRONTIUM ION'
?
'SR 2'            87.620
HOH NON-POLYMER   . WATER
?
'H2 O'            18.015
#
loop_
_pdbx_validate_close_contact.id
_pdbx_validate_close_contact.PDB_model_num
_pdbx_validate_close_contact.auth_atom_id_1
_pdbx_validate_close_contact.auth_asym_id_1
_pdbx_validate_close_contact.auth_comp_id_1
_pdbx_validate_close_contact.auth_seq_id_1
_pdbx_validate_close_contact.PDB_ins_code_1
_pdbx_validate_close_contact.label_alt_id_1
_pdbx_validate_close_contact.auth_atom_id_2
_pdbx_validate_close_contact.auth_asym_id_2
```

```
_pdbx_validate_close_contact.auth_comp_id_2
_pdbx_validate_close_contact.auth_seq_id_2
_pdbx_validate_close_contact.PDB_ins_code_2
_pdbx_validate_close_contact.label_alt_id_2
_pdbx_validate_close_contact.dist
1 1 O A HOH 225 ? ? O A HOH 226 ? ? 2.09
2 1 O B HOH 103 ? ? O B HOH 104 ? ? 2.19
#
_pdbx_validate_rmsd_angle.id                 1
_pdbx_validate_rmsd_angle.PDB_model_num      1
_pdbx_validate_rmsd_angle.auth_atom_id_1     'C1''
_pdbx_validate_rmsd_angle.auth_asym_id_1     A
_pdbx_validate_rmsd_angle.auth_comp_id_1     DC
_pdbx_validate_rmsd_angle.auth_seq_id_1      3
_pdbx_validate_rmsd_angle.PDB_ins_code_1     ?
_pdbx_validate_rmsd_angle.label_alt_id_1     ?
_pdbx_validate_rmsd_angle.auth_atom_id_2     'O4''
_pdbx_validate_rmsd_angle.auth_asym_id_2     A
_pdbx_validate_rmsd_angle.auth_comp_id_2     DC
_pdbx_validate_rmsd_angle.auth_seq_id_2      3
_pdbx_validate_rmsd_angle.PDB_ins_code_2     ?
_pdbx_validate_rmsd_angle.label_alt_id_2     ?
_pdbx_validate_rmsd_angle.auth_atom_id_3     'C4''
_pdbx_validate_rmsd_angle.auth_asym_id_3     A
_pdbx_validate_rmsd_angle.auth_comp_id_3     DC
_pdbx_validate_rmsd_angle.auth_seq_id_3      3
_pdbx_validate_rmsd_angle.PDB_ins_code_3     ?
_pdbx_validate_rmsd_angle.label_alt_id_3     ?
_pdbx_validate_rmsd_angle.angle_deviation    -6.2
#
loop_
_pdbx_entity_nonpoly.entity_id
_pdbx_entity_nonpoly.name
_pdbx_entity_nonpoly.comp_id
2 SPERMINE        SPM
3 'STRONTIUM ION' SR
4 water           HOH
```

```
#
loop_
_atom_site.group_PDB
_atom_site.id
_atom_site.type_symbol
_atom_site.label_atom_id
_atom_site.label_alt_id
_atom_site.label_comp_id
_atom_site.label_asym_id
_atom_site.label_entity_id
_atom_site.label_seq_id
_atom_site.pdbx_PDB_ins_code
_atom_site.Cartn_x
_atom_site.Cartn_y
_atom_site.Cartn_z
_atom_site.occupancy
_atom_site.B_iso_or_equiv
_atom_site.Cartn_x_esd
_atom_site.Cartn_y_esd
_atom_site.Cartn_z_esd
_atom_site.occupancy_esd
_atom_site.B_iso_or_equiv_esd
_atom_site.pdbx_formal_charge
_atom_site.auth_seq_id
_atom_site.auth_comp_id
_atom_site.auth_asym_id
_atom_site.auth_atom_id
_atom_site.pdbx_PDB_model_num
ATOM   1   P  P     . DG  A 1 2  ? 9.239  -10.993 30.734  0.60 64.21  ? ? ? ? ? ? 2   DG  A P     1
ATOM   2   O  OP1   . DG  A 1 2  ? 9.072  -10.756 32.190  0.60 60.29  ? ? ? ? ? ? 2   DG  A OP1   1
ATOM   3   O  OP2   . DG  A 1 2  ? 9.385  -12.395 30.228  0.60 53.30  ? ? ? ? ? ? 2   DG  A OP2   1
ATOM   4   O  'O5'' . DG  A 1 2  ? 8.014  -10.291 29.981  1.00 66.68  ? ? ? ? ? ? 2   DG  A 'O5'' 1
ATOM   5   C  'C5'' . DG  A 1 2  ? 6.825  -9.899  30.702  1.00 55.81  ? ? ? ? ? ? 2   DG  A 'C5'' 1
ATOM   6   C  'C4'' . DG  A 1 2  ? 5.949  -8.961  29.894  1.00 52.59  ? ? ? ? ? ? 2   DG  A 'C4'' 1
ATOM   7   O  'O4'' . DG  A 1 2  ? 6.194  -7.574  30.229  1.00 47.03  ? ? ? ? ? ? 2   DG  A 'O4'' 1
ATOM   8   C  'C3'' . DG  A 1 2  ? 6.021  -9.044  28.371  1.00 47.01  ? ? ? ? ? ? 2   DG  A 'C3'' 1
ATOM   9   O  'O3'' . DG  A 1 2  ? 4.664  -8.805  27.971  1.00 43.95  ? ? ? ? ? ? 2   DG  A 'O3'' 1
```

```
ATOM   10  C  'C2'' . DG  A 1 2  ? 6.953  -7.905  28.004  1.00 43.36  ? ? ? ? ? ? 2   DG  A 'C2'' 1
ATOM   11  C  'C1'' . DG  A 1 2  ? 6.723  -6.873  29.101  1.00 38.71  ? ? ? ? ? ? 2   DG  A 'C1'' 1
ATOM   12  N  N9    . DG  A 1 2  ? 7.941  -6.215  29.539  1.00 38.13  ? ? ? ? ? ? 2   DG  A N9    1
ATOM   13  C  C8    . DG  A 1 2  ? 9.159  -6.806  29.783  1.00 40.71  ? ? ? ? ? ? 2   DG  A C8    1
ATOM   14  N  N7    . DG  A 1 2  ? 10.037 -5.980  30.285  1.00 37.92  ? ? ? ? ? ? 2   DG  A N7    1
ATOM   15  C  C5    . DG  A 1 2  ? 9.326  -4.799  30.471  1.00 34.44  ? ? ? ? ? ? 2   DG  A C5    1
ATOM   16  C  C6    . DG  A 1 2  ? 9.751  -3.550  30.963  1.00 33.16  ? ? ? ? ? ? 2   DG  A C6    1
ATOM   17  O  O6    . DG  A 1 2  ? 10.873 -3.233  31.386  1.00 39.27  ? ? ? ? ? ? 2   DG  A O6    1
ATOM   18  N  N1    . DG  A 1 2  ? 8.717  -2.611  30.947  1.00 31.66  ? ? ? ? ? ? 2   DG  A N1    1
ATOM   19  C  C2    . DG  A 1 2  ? 7.432  -2.858  30.522  1.00 26.82  ? ? ? ? ? ? 2   DG  A C2    1
ATOM   20  N  N2    . DG  A 1 2  ? 6.580  -1.803  30.529  1.00 29.37  ? ? ? ? ? ? 2   DG  A N2    1
ATOM   21  N  N3    . DG  A 1 2  ? 7.035  -4.016  30.020  1.00 32.27  ? ? ? ? ? ? 2   DG  A N3    1
ATOM   22  C  C4    . DG  A 1 2  ? 8.029  -4.933  30.021  1.00 32.15  ? ? ? ? ? ? 2   DG  A C4    1
ATOM   23  P  P     . DC  A 1 3  ? 4.239  -8.657  26.447  1.00 46.97  ? ? ? ? ? ? 3   DC  A P     1
ATOM   24  O  OP1   . DC  A 1 3  ? 2.751  -8.762  26.393  1.00 49.89  ? ? ? ? ? ? 3   DC  A OP1   1
ATOM   25  O  OP2   . DC  A 1 3  ? 5.133  -9.515  25.605  1.00 53.26  ? ? ? ? ? ? 3   DC  A OP2   1
ATOM   26  O  'O5'' . DC  A 1 3  ? 4.520  -7.119  26.124  1.00 43.44  ? ? ? ? ? ? 3   DC  A 'O5'' 1
ATOM   27  C  'C5'' . DC  A 1 3  ? 3.632  -6.106  26.576  1.00 38.31  ? ? ? ? ? ? 3   DC  A 'C5'' 1
ATOM   28  C  'C4'' . DC  A 1 3  ? 4.287  -4.774  26.306  1.00 36.86  ? ? ? ? ? ? 3   DC  A 'C4'' 1
ATOM   29  O  'O4'' . DC  A 1 3  ? 5.592  -4.779  26.930  1.00 42.11  ? ? ? ? ? ? 3   DC  A 'O4'' 1
ATOM   30  C  'C3'' . DC  A 1 3  ? 4.561  -4.493  24.826  1.00 34.95  ? ? ? ? ? ? 3   DC  A 'C3'' 1
ATOM   31  O  'O3'' . DC  A 1 3  ? 3.753  -3.403  24.382  1.00 46.76  ? ? ? ? ? ? 3   DC  A 'O3'' 1
ATOM   32  C  'C2'' . DC  A 1 3  ? 6.019  -4.016  24.799  1.00 40.64  ? ? ? ? ? ? 3   DC  A 'C2'' 1
ATOM   33  C  'C1'' . DC  A 1 3  ? 6.290  -3.756  26.261  1.00 38.02  ? ? ? ? ? ? 3   DC  A 'C1'' 1
ATOM   34  N  N1    . DC  A 1 3  ? 7.686  -3.832  26.664  1.00 41.53  ? ? ? ? ? ? 3   DC  A N1    1
ATOM   35  C  C2    . DC  A 1 3  ? 8.245  -2.738  27.324  1.00 33.63  ? ? ? ? ? ? 3   DC  A C2    1
ATOM   36  O  O2    . DC  A 1 3  ? 7.552  -1.720  27.493  1.00 33.20  ? ? ? ? ? ? 3   DC  A O2    1
ATOM   37  N  N3    . DC  A 1 3  ? 9.537  -2.790  27.708  1.00 34.36  ? ? ? ? ? ? 3   DC  A N3    1
ATOM   38  C  C4    . DC  A 1 3  ? 10.247 -3.901  27.511  1.00 35.83  ? ? ? ? ? ? 3   DC  A C4    1
ATOM   39  N  N4    . DC  A 1 3  ? 11.529 -3.895  27.888  1.00 38.69  ? ? ? ? ? ? 3   DC  A N4    1
ATOM   40  C  C5    . DC  A 1 3  ? 9.691  -5.046  26.867  1.00 36.91  ? ? ? ? ? ? 3   DC  A C5    1
ATOM   41  C  C6    . DC  A 1 3  ? 8.397  -4.990  26.524  1.00 35.23  ? ? ? ? ? ? 3   DC  A C6    1
ATOM   42  P  P     . BZG A 1 4  ? 2.649  -3.637  23.267  1.00 46.63  ? ? ? ? ? ? 4   BZG A P     1
ATOM   43  O  O1P   . BZG A 1 4  ? 3.306  -3.955  21.993  1.00 44.93  ? ? ? ? ? ? 4   BZG A O1P   1
ATOM   44  O  O2P   . BZG A 1 4  ? 1.836  -4.638  23.969  1.00 55.23  ? ? ? ? ? ? 4   BZG A O2P   1
ATOM   45  O  'O5'' . BZG A 1 4  ? 1.933  -2.246  23.191  1.00 39.01  ? ? ? ? ? ? 4   BZG A 'O5'' 1
ATOM   46  C  CZ1   . BZG A 1 4  ? 11.175 0.290   21.777  1.00 37.59  ? ? ? ? ? ? 4   BZG A CZ1   1
```

```
ATOM   47  C   CT1    . BZG A 1 4  ? 12.294 -0.517  21.686  1.00 39.06  ? ? ? ? ? ? 4   BZG A CT1    1
ATOM   48  C   CI     . BZG A 1 4  ? 13.507 0.035   22.067  1.00 33.85  ? ? ? ? ? ? 4   BZG A CI     1
ATOM   49  C   CT2    . BZG A 1 4  ? 13.624 1.374   22.446  1.00 36.75  ? ? ? ? ? ? 4   BZG A CT2    1
ATOM   50  C   CZ2    . BZG A 1 4  ? 12.497 2.161   22.581  1.00 36.31  ? ? ? ? ? ? 4   BZG A CZ2    1
ATOM   51  C   CE     . BZG A 1 4  ? 11.293 1.609   22.203  1.00 36.33  ? ? ? ? ? ? 4   BZG A CE     1
ATOM   52  C   CW     . BZG A 1 4  ? 10.069 2.471   22.307  1.00 34.89  ? ? ? ? ? ? 4   BZG A CW     1
ATOM   53  O   OL     . BZG A 1 4  ? 8.937  1.590   22.071  1.00 37.78  ? ? ? ? ? ? 4   BZG A OL     1
ATOM   54  C   CK     . BZG A 1 4  ? 7.707  2.091   22.327  1.00 35.01  ? ? ? ? ? ? 4   BZG A CK     1
ATOM   55  N   NJ     . BZG A 1 4  ? 7.570  3.379   22.668  1.00 36.53  ? ? ? ? ? ? 4   BZG A NJ     1
ATOM   56  C   CH     . BZG A 1 4  ? 6.358  3.899   22.966  1.00 39.60  ? ? ? ? ? ? 4   BZG A CH     1
ATOM   57  N   NI     . BZG A 1 4  ? 6.258  5.215   23.214  1.00 42.29  ? ? ? ? ? ? 4   BZG A NI     1
ATOM   58  N   NG     . BZG A 1 4  ? 5.249  3.116   22.978  1.00 37.80  ? ? ? ? ? ? 4   BZG A NG     1
ATOM   59  C   CF     . BZG A 1 4  ? 5.361  1.799   22.674  1.00 35.38  ? ? ? ? ? ? 4   BZG A CF     1
ATOM   60  C   CM     . BZG A 1 4  ? 6.607  1.239   22.361  1.00 37.85  ? ? ? ? ? ? 4   BZG A CM     1
ATOM   61  N   NN     . BZG A 1 4  ? 6.455  -0.083  22.118  1.00 39.19  ? ? ? ? ? ? 4   BZG A NN     1
ATOM   62  C   CO     . BZG A 1 4  ? 5.137  -0.349  22.296  1.00 39.06  ? ? ? ? ? ? 4   BZG A CO     1
ATOM   63  N   NE     . BZG A 1 4  ? 4.484  0.814   22.580  1.00 38.14  ? ? ? ? ? ? 4   BZG A NE     1
ATOM   64  C   'CT''  . BZG A 1 4  ? 3.049  1.004   22.890  1.00 40.97  ? ? ? ? ? ? 4   BZG A 'CT'' 1
ATOM   65  O   'OS''  . BZG A 1 4  ? 2.752  0.367   24.126  1.00 41.96  ? ? ? ? ? ? 4   BZG A 'OS'' 1
ATOM   66  C   'CP''  . BZG A 1 4  ? 2.139  0.420   21.818  1.00 42.75  ? ? ? ? ? ? 4   BZG A 'CP'' 1
ATOM   67  C   'C5''  . BZG A 1 4  ? 1.432  -1.656  24.357  1.00 40.60  ? ? ? ? ? ? 4   BZG A 'C5'' 1
ATOM   68  C   'C4''  . BZG A 1 4  ? 1.409  -0.173  24.040  1.00 46.89  ? ? ? ? ? ? 4   BZG A 'C4'' 1
ATOM   69  C   'C3''  . BZG A 1 4  ? 0.883  0.134   22.623  1.00 46.88  ? ? ? ? ? ? 4   BZG A 'C3'' 1
ATOM   70  O   'O3''  . BZG A 1 4  ? 0.164  1.363   22.838  1.00 55.09  ? ? ? ? ? ? 4   BZG A 'O3'' 1
ATOM   71  P   P      . DA  A 1 5  ? -0.648 2.070   21.637  1.00 58.27  ? ? ? ? ? ? 5   DA  A P      1
ATOM   72  O   OP1    . DA  A 1 5  ? -1.834 2.738   22.239  1.00 59.14  ? ? ? ? ? ? 5   DA  A OP1    1
ATOM   73  O   OP2    . DA  A 1 5  ? -0.808 1.074   20.536  1.00 59.17  ? ? ? ? ? ? 5   DA  A OP2    1
ATOM   74  O   'O5''  . DA  A 1 5  ? 0.307  3.265   21.217  1.00 49.03  ? ? ? ? ? ? 5   DA  A 'O5'' 1
ATOM   75  C   'C5''  . DA  A 1 5  ? 0.681  4.240   22.198  1.00 48.58  ? ? ? ? ? ? 5   DA  A 'C5'' 1
ATOM   76  C   'C4''  . DA  A 1 5  ? 1.593  5.267   21.581  1.00 56.65  ? ? ? ? ? ? 5   DA  A 'C4'' 1
ATOM   77  O   'O4''  . DA  A 1 5  ? 2.908  4.711   21.358  1.00 53.36  ? ? ? ? ? ? 5   DA  A 'O4'' 1
ATOM   78  C   'C3''  . DA  A 1 5  ? 1.123  5.797   20.227  1.00 59.46  ? ? ? ? ? ? 5   DA  A 'C3'' 1
ATOM   79  O   'O3''  . DA  A 1 5  ? 1.300  7.208   20.242  1.00 60.34  ? ? ? ? ? ? 5   DA  A 'O3'' 1
ATOM   80  C   'C2''  . DA  A 1 5  ? 2.038  5.114   19.223  1.00 55.75  ? ? ? ? ? ? 5   DA  A 'C2'' 1
ATOM   81  C   'C1''  . DA  A 1 5  ? 3.316  4.936   20.017  1.00 51.87  ? ? ? ? ? ? 5   DA  A 'C1'' 1
ATOM   82  N   N9     . DA  A 1 5  ? 4.126  3.793   19.610  1.00 46.83  ? ? ? ? ? ? 5   DA  A N9     1
ATOM   83  C   C8     . DA  A 1 5  ? 3.701  2.511   19.368  1.00 43.90  ? ? ? ? ? ? 5   DA  A C8     1
```

```
ATOM   84  N N7    . DA  A 1 5  ? 4.669  1.681  19.062 1.00 44.68 ? ? ? ? ? ? 5  DA  A N7    1
ATOM   85  C C5    . DA  A 1 5  ? 5.813  2.463  19.135 1.00 42.49 ? ? ? ? ? ? 5  DA  A C5    1
ATOM   86  C C6    . DA  A 1 5  ? 7.172  2.158  18.979 1.00 36.19 ? ? ? ? ? ? 5  DA  A C6    1
ATOM   87  N N6    . DA  A 1 5  ? 7.619  0.949  18.637 1.00 36.49 ? ? ? ? ? ? 5  DA  A N6    1
ATOM   88  N N1    . DA  A 1 5  ? 8.060  3.168  19.099 1.00 36.14 ? ? ? ? ? ? 5  DA  A N1    1
ATOM   89  C C2    . DA  A 1 5  ? 7.610  4.377  19.460 1.00 36.19 ? ? ? ? ? ? 5  DA  A C2    1
ATOM   90  N N3    . DA  A 1 5  ? 6.358  4.781  19.670 1.00 42.40 ? ? ? ? ? ? 5  DA  A N3    1
ATOM   91  C C4    . DA  A 1 5  ? 5.498  3.760  19.499 1.00 35.08 ? ? ? ? ? ? 5  DA  A C4    1
ATOM   92  P P     . DA  A 1 6  ? 0.679  8.066  19.071 1.00 66.25 ? ? ? ? ? ? 6  DA  A P     1
ATOM   93  O OP1   . DA  A 1 6  ? -0.038 9.214  19.677 1.00 73.87 ? ? ? ? ? ? 6  DA  A OP1   1
ATOM   94  O OP2   . DA  A 1 6  ? -0.026 7.124  18.154 1.00 62.28 ? ? ? ? ? ? 6  DA  A OP2   1
ATOM   95  O 'O5'' . DA  A 1 6  ? 1.965  8.619  18.327 1.00 55.29 ? ? ? ? ? ? 6  DA  A 'O5'' 1
ATOM   96  C 'C5'' . DA  A 1 6  ? 3.074  9.156  19.054 1.00 56.56 ? ? ? ? ? ? 6  DA  A 'C5'' 1
ATOM   97  C 'C4'' . DA  A 1 6  ? 4.288  9.061  18.166 1.00 53.18 ? ? ? ? ? ? 6  DA  A 'C4'' 1
ATOM   98  O 'O4'' . DA  A 1 6  ? 4.605  7.667  17.970 1.00 48.57 ? ? ? ? ? ? 6  DA  A 'O4'' 1
ATOM   99  C 'C3'' . DA  A 1 6  ? 4.033  9.623  16.761 1.00 59.75 ? ? ? ? ? ? 6  DA  A 'C3'' 1
ATOM   100 O 'O3'' . DA  A 1 6  ? 4.671  10.904 16.635 1.00 59.83 ? ? ? ? ? ? 6  DA  A 'O3'' 1
ATOM   101 C 'C2'' . DA  A 1 6  ? 4.466  8.501  15.815 1.00 57.57 ? ? ? ? ? ? 6  DA  A 'C2'' 1
ATOM   102 C 'C1'' . DA  A 1 6  ? 5.241  7.571  16.732 1.00 43.65 ? ? ? ? ? ? 6  DA  A 'C1'' 1
ATOM   103 N N9    . DA  A 1 6  ? 5.326  6.159  16.375 1.00 38.41 ? ? ? ? ? ? 6  DA  A N9    1
ATOM   104 C C8    . DA  A 1 6  ? 4.358  5.195  16.240 1.00 32.72 ? ? ? ? ? ? 6  DA  A C8    1
ATOM   105 N N7    . DA  A 1 6  ? 4.833  4.013  15.917 1.00 34.57 ? ? ? ? ? ? 6  DA  A N7    1
ATOM   106 C C5    . DA  A 1 6  ? 6.209  4.200  15.909 1.00 39.39 ? ? ? ? ? ? 6  DA  A C5    1
ATOM   107 C C6    . DA  A 1 6  ? 7.281  3.323  15.681 1.00 36.78 ? ? ? ? ? ? 6  DA  A C6    1
ATOM   108 N N6    . DA  A 1 6  ? 7.130  2.029  15.402 1.00 36.24 ? ? ? ? ? ? 6  DA  A N6    1
ATOM   109 N N1    . DA  A 1 6  ? 8.529  3.843  15.685 1.00 40.20 ? ? ? ? ? ? 6  DA  A N1    1
ATOM   110 C C2    . DA  A 1 6  ? 8.683  5.138  15.979 1.00 42.40 ? ? ? ? ? ? 6  DA  A C2    1
ATOM   111 N N3    . DA  A 1 6  ? 7.757  6.057  16.242 1.00 44.66 ? ? ? ? ? ? 6  DA  A N3    1
ATOM   112 C C4    . DA  A 1 6  ? 6.526  5.514  16.198 1.00 39.31 ? ? ? ? ? ? 6  DA  A C4    1
ATOM   113 P P     . DT  A 1 7  ? 4.489  11.764 15.293 1.00 63.69 ? ? ? ? ? ? 7  DT  A P     1
ATOM   114 O OP1   . DT  A 1 7  ? 4.824  13.180 15.604 1.00 69.37 ? ? ? ? ? ? 7  DT  A OP1   1
ATOM   115 O OP2   . DT  A 1 7  ? 3.191  11.401 14.662 1.00 58.46 ? ? ? ? ? ? 7  DT  A OP2   1
ATOM   116 O 'O5'' . DT  A 1 7  ? 5.636  11.175 14.367 1.00 46.43 ? ? ? ? ? ? 7  DT  A 'O5'' 1
ATOM   117 C 'C5'' . DT  A 1 7  ? 7.007  11.417 14.718 1.00 51.47 ? ? ? ? ? ? 7  DT  A 'C5'' 1
ATOM   118 C 'C4'' . DT  A 1 7  ? 7.866  10.655 13.748 1.00 46.90 ? ? ? ? ? ? 7  DT  A 'C4'' 1
ATOM   119 O 'O4'' . DT  A 1 7  ? 7.578  9.255  13.892 1.00 48.09 ? ? ? ? ? ? 7  DT  A 'O4'' 1
ATOM   120 C 'C3'' . DT  A 1 7  ? 7.495  10.983 12.306 1.00 43.45 ? ? ? ? ? ? 7  DT  A 'C3'' 1
```

ATOM	121	O	'O3''	.	DT	A	1	7	?	8.490	11.822	11.752	1.00	52.70	?	?	?	?	?	?	?	7	DT	A	'O3''	1
ATOM	122	C	'C2''	.	DT	A	1	7	?	7.380	9.628	11.617	1.00	50.70	?	?	?	?	?	?	?	7	DT	A	'C2''	1
ATOM	123	C	'C1''	.	DT	A	1	7	?	7.913	8.677	12.663	1.00	47.48	?	?	?	?	?	?	?	7	DT	A	'C1''	1
ATOM	124	N	N1	.	DT	A	1	7	?	7.376	7.317	12.639	1.00	41.31	?	?	?	?	?	?	?	7	DT	A	N1	1
ATOM	125	C	C2	.	DT	A	1	7	?	8.289	6.297	12.506	1.00	41.88	?	?	?	?	?	?	?	7	DT	A	C2	1
ATOM	126	O	O2	.	DT	A	1	7	?	9.493	6.487	12.429	1.00	42.96	?	?	?	?	?	?	?	7	DT	A	O2	1
ATOM	127	N	N3	.	DT	A	1	7	?	7.745	5.043	12.494	1.00	38.18	?	?	?	?	?	?	?	7	DT	A	N3	1
ATOM	128	C	C4	.	DT	A	1	7	?	6.413	4.714	12.593	1.00	39.17	?	?	?	?	?	?	?	7	DT	A	C4	1
ATOM	129	O	O4	.	DT	A	1	7	?	6.074	3.542	12.534	1.00	42.62	?	?	?	?	?	?	?	7	DT	A	O4	1
ATOM	130	C	C5	.	DT	A	1	7	?	5.502	5.840	12.688	1.00	39.25	?	?	?	?	?	?	?	7	DT	A	C5	1
ATOM	131	C	C7	.	DT	A	1	7	?	4.033	5.582	12.805	1.00	37.23	?	?	?	?	?	?	?	7	DT	A	C7	1
ATOM	132	C	C6	.	DT	A	1	7	?	6.024	7.068	12.690	1.00	36.63	?	?	?	?	?	?	?	7	DT	A	C6	1
ATOM	133	P	P	.	DT	A	1	8	?	8.228	12.450	10.338	1.00	52.32	?	?	?	?	?	?	?	8	DT	A	P	1
ATOM	134	O	OP1	.	DT	A	1	8	?	8.908	13.746	10.289	1.00	52.05	?	?	?	?	?	?	?	8	DT	A	OP1	1
ATOM	135	O	OP2	.	DT	A	1	8	?	6.757	12.374	10.063	1.00	55.19	?	?	?	?	?	?	?	8	DT	A	OP2	1
ATOM	136	O	'O5''	.	DT	A	1	8	?	8.910	11.384	9.375	1.00	46.41	?	?	?	?	?	?	?	8	DT	A	'O5''	1
ATOM	137	C	'C5''	.	DT	A	1	8	?	10.246	10.929	9.583	1.00	44.52	?	?	?	?	?	?	?	8	DT	A	'C5''	1
ATOM	138	C	'C4''	.	DT	A	1	8	?	10.482	9.804	8.610	1.00	43.89	?	?	?	?	?	?	?	8	DT	A	'C4''	1
ATOM	139	O	'O4''	.	DT	A	1	8	?	9.790	8.651	9.114	1.00	42.41	?	?	?	?	?	?	?	8	DT	A	'O4''	1
ATOM	140	C	'C3''	.	DT	A	1	8	?	9.850	10.104	7.249	1.00	48.97	?	?	?	?	?	?	?	8	DT	A	'C3''	1
ATOM	141	O	'O3''	.	DT	A	1	8	?	10.867	10.316	6.277	1.00	53.05	?	?	?	?	?	?	?	8	DT	A	'O3''	1
ATOM	142	C	'C2''	.	DT	A	1	8	?	8.964	8.904	6.950	1.00	40.46	?	?	?	?	?	?	?	8	DT	A	'C2''	1
ATOM	143	C	'C1''	.	DT	A	1	8	?	9.334	7.892	8.018	1.00	41.47	?	?	?	?	?	?	?	8	DT	A	'C1''	1
ATOM	144	N	N1	.	DT	A	1	8	?	8.211	7.065	8.474	1.00	38.36	?	?	?	?	?	?	?	8	DT	A	N1	1
ATOM	145	C	C2	.	DT	A	1	8	?	8.433	5.721	8.676	1.00	39.71	?	?	?	?	?	?	?	8	DT	A	C2	1
ATOM	146	O	O2	.	DT	A	1	8	?	9.530	5.206	8.573	1.00	42.69	?	?	?	?	?	?	?	8	DT	A	O2	1
ATOM	147	N	N3	.	DT	A	1	8	?	7.323	5.008	9.045	1.00	37.19	?	?	?	?	?	?	?	8	DT	A	N3	1
ATOM	148	C	C4	.	DT	A	1	8	?	6.055	5.502	9.280	1.00	42.23	?	?	?	?	?	?	?	8	DT	A	C4	1
ATOM	149	O	O4	.	DT	A	1	8	?	5.156	4.739	9.627	1.00	44.03	?	?	?	?	?	?	?	8	DT	A	O4	1
ATOM	150	C	C5	.	DT	A	1	8	?	5.887	6.917	9.020	1.00	38.33	?	?	?	?	?	?	?	8	DT	A	C5	1
ATOM	151	C	C7	.	DT	A	1	8	?	4.544	7.541	9.235	1.00	43.91	?	?	?	?	?	?	?	8	DT	A	C7	1
ATOM	152	C	C6	.	DT	A	1	8	?	6.956	7.612	8.618	1.00	36.11	?	?	?	?	?	?	?	8	DT	A	C6	1
ATOM	153	O	O1P	.	D3N	A	1	9	?	9.151	11.502	5.034	1.00	57.06	?	?	?	?	?	?	?	9	D3N	A	O1P	1
ATOM	154	P	P	.	D3N	A	1	9	?	10.467	10.803	4.830	1.00	59.57	?	?	?	?	?	?	?	9	D3N	A	P	1
ATOM	155	O	O2P	.	D3N	A	1	9	?	11.529	11.563	4.178	1.00	57.35	?	?	?	?	?	?	?	9	D3N	A	O2P	1
ATOM	156	O	'O5''	.	D3N	A	1	9	?	10.132	9.407	4.099	1.00	45.73	?	?	?	?	?	?	?	9	D3N	A	'O5''	1
ATOM	157	C	'C5''	.	D3N	A	1	9	?	9.339	9.445	2.962	1.00	56.53	?	?	?	?	?	?	?	9	D3N	A	'C5''	1

ATOM	158	C	'C4''	.	D3N	A	1	9	?	9.661	8.312	2.011	1.00	49.95	?	?	?	?	?	?	9	D3N	A	'C4''	1
ATOM	159	O	'O4''	.	D3N	A	1	9	?	8.863	7.224	2.387	1.00	46.67	?	?	?	?	?	?	9	D3N	A	'O4''	1
ATOM	160	C	'C3''	.	D3N	A	1	9	?	9.320	8.677	0.601	1.00	47.42	?	?	?	?	?	?	9	D3N	A	'C3''	1
ATOM	161	O	'O3''	.	D3N	A	1	9	?	10.640	8.976	-0.003	1.00	54.09	?	?	?	?	?	?	9	D3N	A	'O3''	1
ATOM	162	C	'C2''	.	D3N	A	1	9	?	8.658	7.422	0.042	1.00	44.59	?	?	?	?	?	?	9	D3N	A	'C2''	1
ATOM	163	C	'C1''	.	D3N	A	1	9	?	8.417	6.534	1.230	1.00	46.91	?	?	?	?	?	?	9	D3N	A	'C1''	1
ATOM	164	N	N1	.	D3N	A	1	9	?	7.078	6.124	1.580	1.00	36.46	?	?	?	?	?	?	9	D3N	A	N1	1
ATOM	165	C	C2	.	D3N	A	1	9	?	6.110	7.057	1.685	1.00	38.72	?	?	?	?	?	?	9	D3N	A	C2	1
ATOM	166	O	O2	.	D3N	A	1	9	?	6.297	8.251	1.417	1.00	48.10	?	?	?	?	?	?	9	D3N	A	O2	1
ATOM	167	N	N3	.	D3N	A	1	9	?	4.887	6.635	2.099	1.00	41.54	?	?	?	?	?	?	9	D3N	A	N3	1
ATOM	168	C	C4	.	D3N	A	1	9	?	4.594	5.301	2.451	1.00	36.92	?	?	?	?	?	?	9	D3N	A	C4	1
ATOM	169	C	C5	.	D3N	A	1	9	?	5.607	4.371	2.331	1.00	36.17	?	?	?	?	?	?	9	D3N	A	C5	1
ATOM	170	C	C6	.	D3N	A	1	9	?	6.891	4.763	1.888	1.00	36.76	?	?	?	?	?	?	9	D3N	A	C6	1
ATOM	171	C	C7	.	D3N	A	1	9	?	7.850	3.823	1.781	1.00	37.28	?	?	?	?	?	?	9	D3N	A	C7	1
ATOM	172	C	C8	.	D3N	A	1	9	?	7.548	2.482	2.091	1.00	38.67	?	?	?	?	?	?	9	D3N	A	C8	1
ATOM	173	C	C9	.	D3N	A	1	9	?	6.313	2.075	2.503	1.00	34.82	?	?	?	?	?	?	9	D3N	A	C9	1
ATOM	174	C	C10	.	D3N	A	1	9	?	5.329	3.022	2.649	1.00	36.15	?	?	?	?	?	?	9	D3N	A	C10	1
ATOM	175	C	C11	.	D3N	A	1	9	?	4.040	2.682	3.115	1.00	39.57	?	?	?	?	?	?	9	D3N	A	C11	1
ATOM	176	C	C12	.	D3N	A	1	9	?	3.039	3.617	3.226	1.00	43.64	?	?	?	?	?	?	9	D3N	A	C12	1
ATOM	177	C	C13	.	D3N	A	1	9	?	3.306	4.953	2.885	1.00	37.78	?	?	?	?	?	?	9	D3N	A	C13	1
ATOM	178	P	P	.	DG	A	1	10	?	10.766	9.455	-1.541	1.00	52.40	?	?	?	?	?	?	10	DG	A	P	1
ATOM	179	O	OP1	.	DG	A	1	10	?	11.926	10.387	-1.619	1.00	52.96	?	?	?	?	?	?	10	DG	A	OP1	1
ATOM	180	O	OP2	.	DG	A	1	10	?	9.429	9.942	-2.020	1.00	49.83	?	?	?	?	?	?	10	DG	A	OP2	1
ATOM	181	O	'O5''	.	DG	A	1	10	?	11.122	8.085	-2.299	1.00	46.54	?	?	?	?	?	?	10	DG	A	'O5''	1
ATOM	182	C	'C5''	.	DG	A	1	10	?	12.098	7.130	-1.833	1.00	40.41	?	?	?	?	?	?	10	DG	A	'C5''	1
ATOM	183	C	'C4''	.	DG	A	1	10	?	11.817	5.781	-2.467	1.00	46.19	?	?	?	?	?	?	10	DG	A	'C4''	1
ATOM	184	O	'O4''	.	DG	A	1	10	?	10.495	5.337	-2.050	1.00	41.89	?	?	?	?	?	?	10	DG	A	'O4''	1
ATOM	185	C	'C3''	.	DG	A	1	10	?	11.772	5.751	-3.999	1.00	42.11	?	?	?	?	?	?	10	DG	A	'C3''	1
ATOM	186	O	'O3''	.	DG	A	1	10	?	11.986	4.386	-4.405	1.00	43.10	?	?	?	?	?	?	10	DG	A	'O3''	1
ATOM	187	C	'C2''	.	DG	A	1	10	?	10.312	6.041	-4.278	1.00	37.56	?	?	?	?	?	?	10	DG	A	'C2''	1
ATOM	188	C	'C1''	.	DG	A	1	10	?	9.649	5.223	-3.182	1.00	39.74	?	?	?	?	?	?	10	DG	A	'C1''	1
ATOM	189	N	N9	.	DG	A	1	10	?	8.318	5.670	-2.791	1.00	36.64	?	?	?	?	?	?	10	DG	A	N9	1
ATOM	190	C	C8	.	DG	A	1	10	?	7.813	6.946	-2.904	1.00	38.12	?	?	?	?	?	?	10	DG	A	C8	1
ATOM	191	N	N7	.	DG	A	1	10	?	6.596	7.058	-2.443	1.00	33.59	?	?	?	?	?	?	10	DG	A	N7	1
ATOM	192	C	C5	.	DG	A	1	10	?	6.277	5.778	-1.999	1.00	32.82	?	?	?	?	?	?	10	DG	A	C5	1
ATOM	193	C	C6	.	DG	A	1	10	?	5.098	5.289	-1.387	1.00	33.36	?	?	?	?	?	?	10	DG	A	C6	1
ATOM	194	O	O6	.	DG	A	1	10	?	4.056	5.900	-1.124	1.00	33.85	?	?	?	?	?	?	10	DG	A	O6	1

```
ATOM   195 N  N1     . DG  A 1 10 ? 5.178  3.921   -1.139  1.00 32.10  ? ? ? ? ? ? 10  DG  A N1     1
ATOM   196 C  C2     . DG  A 1 10 ? 6.298  3.154   -1.316  1.00 32.80  ? ? ? ? ? ? 10  DG  A C2     1
ATOM   197 N  N2     . DG  A 1 10 ? 6.170  1.847   -1.011  1.00 30.07  ? ? ? ? ? ? 10  DG  A N2     1
ATOM   198 N  N3     . DG  A 1 10 ? 7.410  3.596   -1.892  1.00 30.93  ? ? ? ? ? ? 10  DG  A N3     1
ATOM   199 C  C4     . DG  A 1 10 ? 7.337  4.916   -2.184  1.00 36.17  ? ? ? ? ? ? 10  DG  A C4     1
ATOM   200 P  P      . DC  A 1 11 ? 12.576 3.993   -5.867  1.00 45.72  ? ? ? ? ? ? 11  DC  A P      1
ATOM   201 O  OP1    . DC  A 1 11 ? 14.013 3.708   -5.707  1.00 55.46  ? ? ? ? ? ? 11  DC  A OP1    1
ATOM   202 O  OP2    . DC  A 1 11 ? 12.140 4.979   -6.893  1.00 40.18  ? ? ? ? ? ? 11  DC  A OP2    1
ATOM   203 O  'O5''  . DC  A 1 11 ? 11.897 2.569   -6.086  1.00 42.94  ? ? ? ? ? ? 11  DC  A 'O5'' 1
ATOM   204 C  'C5''  . DC  A 1 11 ? 12.323 1.462   -5.304  1.00 35.76  ? ? ? ? ? ? 11  DC  A 'C5'' 1
ATOM   205 C  'C4''  . DC  A 1 11 ? 11.244 0.407   -5.260  1.00 38.62  ? ? ? ? ? ? 11  DC  A 'C4'' 1
ATOM   206 O  'O4''  . DC  A 1 11 ? 10.071 0.969   -4.638  1.00 38.55  ? ? ? ? ? ? 11  DC  A 'O4'' 1
ATOM   207 C  'C3''  . DC  A 1 11 ? 10.785 -0.117  -6.624  1.00 41.61  ? ? ? ? ? ? 11  DC  A 'C3'' 1
ATOM   208 O  'O3''  . DC  A 1 11 ? 10.597 -1.537  -6.580  1.00 36.55  ? ? ? ? ? ? 11  DC  A 'O3'' 1
ATOM   209 C  'C2''  . DC  A 1 11 ? 9.399  0.483   -6.801  1.00 35.59  ? ? ? ? ? ? 11  DC  A 'C2'' 1
ATOM   210 C  'C1''  . DC  A 1 11 ? 8.928  0.621   -5.361  1.00 32.15  ? ? ? ? ? ? 11  DC  A 'C1'' 1
ATOM   211 N  N1     . DC  A 1 11 ? 7.930  1.697   -5.184  1.00 32.78  ? ? ? ? ? ? 11  DC  A N1     1
ATOM   212 C  C2     . DC  A 1 11 ? 6.724  1.419   -4.517  1.00 33.71  ? ? ? ? ? ? 11  DC  A C2     1
ATOM   213 O  O2     . DC  A 1 11 ? 6.588  0.324   -3.936  1.00 35.86  ? ? ? ? ? ? 11  DC  A O2     1
ATOM   214 N  N3     . DC  A 1 11 ? 5.767  2.369   -4.474  1.00 30.73  ? ? ? ? ? ? 11  DC  A N3     1
ATOM   215 C  C4     . DC  A 1 11 ? 5.992  3.574   -5.013  1.00 31.53  ? ? ? ? ? ? 11  DC  A C4     1
ATOM   216 N  N4     . DC  A 1 11 ? 5.017  4.482   -4.943  1.00 29.21  ? ? ? ? ? ? 11  DC  A N4     1
ATOM   217 C  C5     . DC  A 1 11 ? 7.212  3.886   -5.677  1.00 29.13  ? ? ? ? ? ? 11  DC  A C5     1
ATOM   218 C  C6     . DC  A 1 11 ? 8.141  2.926   -5.747  1.00 34.19  ? ? ? ? ? ? 11  DC  A C6     1
ATOM   219 P  P      . DG  A 1 12 ? 11.039 -2.438  -7.862  1.00 43.78  ? ? ? ? ? ? 12  DG  A P      1
ATOM   220 O  OP1    . DG  A 1 12 ? 10.876 -3.861  -7.477  1.00 44.28  ? ? ? ? ? ? 12  DG  A OP1    1
ATOM   221 O  OP2    . DG  A 1 12 ? 12.357 -1.933  -8.370  1.00 40.00  ? ? ? ? ? ? 12  DG  A OP2    1
ATOM   222 O  'O5''  . DG  A 1 12 ? 9.983  -2.054  -8.975  1.00 40.27  ? ? ? ? ? ? 12  DG  A 'O5'' 1
ATOM   223 C  'C5''  . DG  A 1 12 ? 8.619  -2.501  -8.874  1.00 43.08  ? ? ? ? ? ? 12  DG  A 'C5'' 1
ATOM   224 C  'C4''  . DG  A 1 12 ? 7.882  -2.053  -10.106 1.00 39.93  ? ? ? ? ? ? 12  DG  A 'C4'' 1
ATOM   225 O  'O4''  . DG  A 1 12 ? 7.921  -0.607  -10.134 1.00 36.76  ? ? ? ? ? ? 12  DG  A 'O4'' 1
ATOM   226 C  'C3''  . DG  A 1 12 ? 8.562  -2.505  -11.397 1.00 40.02  ? ? ? ? ? ? 12  DG  A 'C3'' 1
ATOM   227 O  'O3''  . DG  A 1 12 ? 7.910  -3.662  -11.897 1.00 39.85  ? ? ? ? ? ? 12  DG  A 'O3'' 1
ATOM   228 C  'C2''  . DG  A 1 12 ? 8.360  -1.335  -12.320 1.00 38.74  ? ? ? ? ? ? 12  DG  A 'C2'' 1
ATOM   229 C  'C1''  . DG  A 1 12 ? 8.439  -0.157  -11.368 1.00 39.35  ? ? ? ? ? ? 12  DG  A 'C1'' 1
ATOM   230 N  N9     . DG  A 1 12 ? 9.781  0.359   -11.119 1.00 37.62  ? ? ? ? ? ? 12  DG  A N9     1
ATOM   231 C  C8     . DG  A 1 12 ? 10.976 -0.269  -11.381 1.00 44.00  ? ? ? ? ? ? 12  DG  A C8     1
```

```
ATOM   232 N   N7     . DG  A 1 12 ? 12.012 0.429   -11.001 1.00 42.04  ? ? ? ? ? ? 12  DG  A N7    1
ATOM   233 C   C5     . DG  A 1 12 ? 11.465 1.537   -10.366 1.00 41.36  ? ? ? ? ? ? 12  DG  A C5    1
ATOM   234 C   C6     . DG  A 1 12 ? 12.096 2.635   -9.733  1.00 46.68  ? ? ? ? ? ? 12  DG  A C6    1
ATOM   235 O   O6     . DG  A 1 12 ? 13.304 2.848   -9.585  1.00 44.90  ? ? ? ? ? ? 12  DG  A O6    1
ATOM   236 N   N1     . DG  A 1 12 ? 11.166 3.555   -9.257  1.00 37.19  ? ? ? ? ? ? 12  DG  A N1    1
ATOM   237 C   C2     . DG  A 1 12 ? 9.805  3.432   -9.369  1.00 37.43  ? ? ? ? ? ? 12  DG  A C2    1
ATOM   238 N   N2     . DG  A 1 12 ? 9.081  4.437   -8.858  1.00 34.27  ? ? ? ? ? ? 12  DG  A N2    1
ATOM   239 N   N3     . DG  A 1 12 ? 9.202  2.383   -9.905  1.00 34.44  ? ? ? ? ? ? 12  DG  A N3    1
ATOM   240 C   C4     . DG  A 1 12 ? 10.088 1.495   -10.408 1.00 38.94  ? ? ? ? ? ? 12  DG  A C4    1
ATOM   241 O   'O3'' . DC  B 1 1  ? -6.095 1.968   -3.008  1.00 104.56 ? ? ? ? ? ? 13  DC  B 'O3'' 1
ATOM   242 P   P      . DG  B 1 2  ? -4.822 1.409   -3.812  1.00 105.65 ? ? ? ? ? ? 14  DG  B P     1
ATOM   243 O   OP1    . DG  B 1 2  ? -3.885 2.550   -4.035  1.00 96.18  ? ? ? ? ? ? 14  DG  B OP1   1
ATOM   244 O   OP2    . DG  B 1 2  ? -5.315 0.603   -4.976  1.00 78.96  ? ? ? ? ? ? 14  DG  B OP2   1
ATOM   245 O   'O5'' . DG  B 1 2  ? -4.185 0.364   -2.794  1.00 74.48  ? ? ? ? ? ? 14  DG  B 'O5'' 1
ATOM   246 C   'C5'' . DG  B 1 2  ? -3.928 -0.984  -3.220  1.00 57.72  ? ? ? ? ? ? 14  DG  B 'C5'' 1
ATOM   247 C   'C4'' . DG  B 1 2  ? -2.876 -1.621  -2.345  1.00 54.83  ? ? ? ? ? ? 14  DG  B 'C4'' 1
ATOM   248 O   'O4'' . DG  B 1 2  ? -1.555 -1.129  -2.675  1.00 52.37  ? ? ? ? ? ? 14  DG  B 'O4'' 1
ATOM   249 C   'C3'' . DG  B 1 2  ? -3.040 -1.398  -0.847  1.00 47.69  ? ? ? ? ? ? 14  DG  B 'C3'' 1
ATOM   250 O   'O3'' . DG  B 1 2  ? -2.610 -2.634  -0.294  1.00 47.75  ? ? ? ? ? ? 14  DG  B 'O3'' 1
ATOM   251 C   'C2'' . DG  B 1 2  ? -2.064 -0.272  -0.550  1.00 44.50  ? ? ? ? ? ? 14  DG  B 'C2'' 1
ATOM   252 C   'C1'' . DG  B 1 2  ? -0.942 -0.523  -1.550  1.00 46.21  ? ? ? ? ? ? 14  DG  B 'C1'' 1
ATOM   253 N   N9     . DG  B 1 2  ? -0.301 0.694   -2.022  1.00 32.96  ? ? ? ? ? ? 14  DG  B N9    1
ATOM   254 C   C8     . DG  B 1 2  ? -0.949 1.860   -2.348  1.00 38.11  ? ? ? ? ? ? 14  DG  B C8    1
ATOM   255 N   N7     . DG  B 1 2  ? -0.162 2.749   -2.890  1.00 34.89  ? ? ? ? ? ? 14  DG  B N7    1
ATOM   256 C   C5     . DG  B 1 2  ? 1.083  2.136   -2.918  1.00 35.32  ? ? ? ? ? ? 14  DG  B C5    1
ATOM   257 C   C6     . DG  B 1 2  ? 2.324  2.613   -3.403  1.00 32.74  ? ? ? ? ? ? 14  DG  B C6    1
ATOM   258 O   O6     . DG  B 1 2  ? 2.594  3.731   -3.859  1.00 38.67  ? ? ? ? ? ? 14  DG  B O6    1
ATOM   259 N   N1     . DG  B 1 2  ? 3.340  1.683   -3.204  1.00 33.06  ? ? ? ? ? ? 14  DG  B N1    1
ATOM   260 C   C2     . DG  B 1 2  ? 3.155  0.400   -2.738  1.00 30.22  ? ? ? ? ? ? 14  DG  B C2    1
ATOM   261 N   N2     . DG  B 1 2  ? 4.259  -0.379  -2.677  1.00 32.78  ? ? ? ? ? ? 14  DG  B N2    1
ATOM   262 N   N3     . DG  B 1 2  ? 1.989  -0.073  -2.324  1.00 33.84  ? ? ? ? ? ? 14  DG  B N3    1
ATOM   263 C   C4     . DG  B 1 2  ? 1.002  0.844   -2.441  1.00 34.05  ? ? ? ? ? ? 14  DG  B C4    1
ATOM   264 P   P      . DC  B 1 3  ? -2.707 -2.910  1.247   1.00 52.14  ? ? ? ? ? ? 15  DC  B P     1
ATOM   265 O   OP1    . DC  B 1 3  ? -2.794 -4.367  1.421   1.00 52.06  ? ? ? ? ? ? 15  DC  B OP1   1
ATOM   266 O   OP2    . DC  B 1 3  ? -3.707 -1.995  1.828   1.00 47.13  ? ? ? ? ? ? 15  DC  B OP2   1
ATOM   267 O   'O5'' . DC  B 1 3  ? -1.258 -2.522  1.759   1.00 45.23  ? ? ? ? ? ? 15  DC  B 'O5'' 1
ATOM   268 C   'C5'' . DC  B 1 3  ? -0.153 -3.310  1.307   1.00 46.29  ? ? ? ? ? ? 15  DC  B 'C5'' 1
```

```
ATOM   269 C   'C4'' . DC  B 1 3  ? 1.130  -2.589  1.622   1.00 42.32  ? ? ? ? ? ? 15  DC  B 'C4'' 1
ATOM   270 O   'O4'' . DC  B 1 3  ? 1.192  -1.371  0.856   1.00 45.55  ? ? ? ? ? ? 15  DC  B 'O4'' 1
ATOM   271 C   'C3'' . DC  B 1 3  ? 1.296  -2.166  3.088   1.00 47.96  ? ? ? ? ? ? 15  DC  B 'C3'' 1
ATOM   272 O   'O3'' . DC  B 1 3  ? 2.440  -2.809  3.642   1.00 56.55  ? ? ? ? ? ? 15  DC  B 'O3'' 1
ATOM   273 C   'C2'' . DC  B 1 3  ? 1.637  -0.684  3.018   1.00 42.57  ? ? ? ? ? ? 15  DC  B 'C2'' 1
ATOM   274 C   'C1'' . DC  B 1 3  ? 2.050  -0.520  1.577   1.00 36.84  ? ? ? ? ? ? 15  DC  B 'C1'' 1
ATOM   275 N   N1    . DC  B 1 3  ? 1.874  0.836   1.061   1.00 37.49  ? ? ? ? ? ? 15  DC  B N1    1
ATOM   276 C   C2    . DC  B 1 3  ? 2.964  1.476   0.473   1.00 35.12  ? ? ? ? ? ? 15  DC  B C2    1
ATOM   277 O   O2    . DC  B 1 3  ? 4.049  0.859   0.377   1.00 30.92  ? ? ? ? ? ? 15  DC  B O2    1
ATOM   278 N   N3    . DC  B 1 3  ? 2.818  2.739   0.015   1.00 30.96  ? ? ? ? ? ? 15  DC  B N3    1
ATOM   279 C   C4    . DC  B 1 3  ? 1.651  3.370   0.160   1.00 35.63  ? ? ? ? ? ? 15  DC  B C4    1
ATOM   280 N   N4    . DC  B 1 3  ? 1.553  4.608   -0.306  1.00 31.76  ? ? ? ? ? ? 15  DC  B N4    1
ATOM   281 C   C5    . DC  B 1 3  ? 0.510  2.724   0.715   1.00 31.16  ? ? ? ? ? ? 15  DC  B C5    1
ATOM   282 C   C6    . DC  B 1 3  ? 0.664  1.466   1.147   1.00 30.61  ? ? ? ? ? ? 15  DC  B C6    1
ATOM   283 P   P     . BZG B 1 4  ? 2.251  -3.983  4.705   1.00 59.25  ? ? ? ? ? ? 16  BZG B P     1
ATOM   284 O   O1P   . BZG B 1 4  ? 1.758  -3.334  5.965   1.00 59.84  ? ? ? ? ? ? 16  BZG B O1P   1
ATOM   285 O   O2P   . BZG B 1 4  ? 1.431  -4.994  3.984   1.00 58.44  ? ? ? ? ? ? 16  BZG B O2P   1
ATOM   286 O   'O5'' . BZG B 1 4  ? 3.716  -4.525  4.868   1.00 53.19  ? ? ? ? ? ? 16  BZG B 'O5'' 1
ATOM   287 C   CZ1   . BZG B 1 4  ? 6.738  6.622   5.264   1.00 37.15  ? ? ? ? ? ? 16  BZG B CZ1   1
ATOM   288 C   CT1   . BZG B 1 4  ? 5.819  7.656   5.321   1.00 40.05  ? ? ? ? ? ? 16  BZG B CT1   1
ATOM   289 C   CI    . BZG B 1 4  ? 4.485  7.425   5.686   1.00 46.13  ? ? ? ? ? ? 16  BZG B CI    1
ATOM   290 C   CT2   . BZG B 1 4  ? 4.042  6.151   6.055   1.00 42.60  ? ? ? ? ? ? 16  BZG B CT2   1
ATOM   291 C   CZ2   . BZG B 1 4  ? 4.968  5.094   6.004   1.00 41.87  ? ? ? ? ? ? 16  BZG B CZ2   1
ATOM   292 C   CE    . BZG B 1 4  ? 6.295  5.331   5.627   1.00 36.41  ? ? ? ? ? ? 16  BZG B CE    1
ATOM   293 C   CW    . BZG B 1 4  ? 7.263  4.189   5.618   1.00 39.93  ? ? ? ? ? ? 16  BZG B CW    1
ATOM   294 O   OL    . BZG B 1 4  ? 6.564  2.941   5.856   1.00 43.12  ? ? ? ? ? ? 16  BZG B OL    1
ATOM   295 C   CK    . BZG B 1 4  ? 7.211  1.733   5.634   1.00 40.04  ? ? ? ? ? ? 16  BZG B CK    1
ATOM   296 N   NJ    . BZG B 1 4  ? 8.525  1.744   5.314   1.00 43.22  ? ? ? ? ? ? 16  BZG B NJ    1
ATOM   297 C   CH    . BZG B 1 4  ? 9.182  0.577   5.068   1.00 43.83  ? ? ? ? ? ? 16  BZG B CH    1
ATOM   298 N   NI    . BZG B 1 4  ? 10.491 0.578   4.779   1.00 45.60  ? ? ? ? ? ? 16  BZG B NI    1
ATOM   299 N   NG    . BZG B 1 4  ? 8.549  -0.623  5.114   1.00 42.53  ? ? ? ? ? ? 16  BZG B NG    1
ATOM   300 C   CF    . BZG B 1 4  ? 7.252  -0.663  5.426   1.00 39.41  ? ? ? ? ? ? 16  BZG B CF    1
ATOM   301 C   CM    . BZG B 1 4  ? 6.539  0.509   5.663   1.00 37.34  ? ? ? ? ? ? 16  BZG B CM    1
ATOM   302 N   NN    . BZG B 1 4  ? 5.264  0.119   5.947   1.00 45.16  ? ? ? ? ? ? 16  BZG B NN    1
ATOM   303 C   CO    . BZG B 1 4  ? 5.213  -1.240  5.844   1.00 37.18  ? ? ? ? ? ? 16  BZG B CO    1
ATOM   304 N   NE    . BZG B 1 4  ? 6.447  -1.687  5.551   1.00 41.81  ? ? ? ? ? ? 16  BZG B NE    1
ATOM   305 C   'CT'' . BZG B 1 4  ? 6.866  -3.092  5.296   1.00 48.18  ? ? ? ? ? ? 16  BZG B 'CT'' 1
```

```
ATOM   306 O  'OS'' . BZG B 1 4  ? 6.296  -3.544  4.063   1.00 50.49  ? ? ? ? ? ? 16  BZG B 'OS'' 1
ATOM   307 C  'CP'' . BZG B 1 4  ? 6.358  -4.070  6.336   1.00 51.14  ? ? ? ? ? ? 16  BZG B 'CP'' 1
ATOM   308 C  'C5'' . BZG B 1 4  ? 4.448  -5.049  3.759   1.00 55.03  ? ? ? ? ? ? 16  BZG B 'C5'' 1
ATOM   309 C  'C4'' . BZG B 1 4  ? 5.918  -4.932  4.128   1.00 54.38  ? ? ? ? ? ? 16  BZG B 'C4'' 1
ATOM   310 C  'C3'' . BZG B 1 4  ? 6.225  -5.365  5.557   1.00 49.35  ? ? ? ? ? ? 16  BZG B 'C3'' 1
ATOM   311 O  'O3'' . BZG B 1 4  ? 7.520  -5.968  5.495   1.00 53.57  ? ? ? ? ? ? 16  BZG B 'O3'' 1
ATOM   312 P  P     . DA  B 1 5  ? 8.189  -6.628  6.814   1.00 57.46  ? ? ? ? ? ? 17  DA  B P     1
ATOM   313 O  OP1   . DA  B 1 5  ? 9.109  -7.704  6.368   1.00 58.86  ? ? ? ? ? ? 17  DA  B OP1   1
ATOM   314 O  OP2   . DA  B 1 5  ? 7.101  -6.947  7.780   1.00 55.69  ? ? ? ? ? ? 17  DA  B OP2   1
ATOM   315 O  'O5'' . DA  B 1 5  ? 9.148  -5.476  7.354   1.00 51.31  ? ? ? ? ? ? 17  DA  B 'O5'' 1
ATOM   316 C  'C5'' . DA  B 1 5  ? 10.239 -4.994  6.552   1.00 51.08  ? ? ? ? ? ? 17  DA  B 'C5'' 1
ATOM   317 C  'C4'' . DA  B 1 5  ? 10.920 -3.871  7.294   1.00 53.59  ? ? ? ? ? ? 17  DA  B 'C4'' 1
ATOM   318 O  'O4'' . DA  B 1 5  ? 10.038 -2.714  7.363   1.00 48.85  ? ? ? ? ? ? 17  DA  B 'O4'' 1
ATOM   319 C  'C3'' . DA  B 1 5  ? 11.227 -4.235  8.747   1.00 51.37  ? ? ? ? ? ? 17  DA  B 'C3'' 1
ATOM   320 O  'O3'' . DA  B 1 5  ? 12.439 -3.577  9.077   1.00 65.84  ? ? ? ? ? ? 17  DA  B 'O3'' 1
ATOM   321 C  'C2'' . DA  B 1 5  ? 10.084 -3.589  9.514   1.00 43.01  ? ? ? ? ? ? 17  DA  B 'C2'' 1
ATOM   322 C  'C1'' . DA  B 1 5  ? 9.935  -2.310  8.712   1.00 45.18  ? ? ? ? ? ? 17  DA  B 'C1'' 1
ATOM   323 N  N9    . DA  B 1 5  ? 8.669  -1.606  8.891   1.00 38.03  ? ? ? ? ? ? 17  DA  B N9    1
ATOM   324 C  C8    . DA  B 1 5  ? 7.432  -2.107  9.213   1.00 42.14  ? ? ? ? ? ? 17  DA  B C8    1
ATOM   325 N  N7    . DA  B 1 5  ? 6.518  -1.183  9.394   1.00 44.19  ? ? ? ? ? ? 17  DA  B N7    1
ATOM   326 C  C5    . DA  B 1 5  ? 7.198  0.006   9.158   1.00 37.91  ? ? ? ? ? ? 17  DA  B C5    1
ATOM   327 C  C6    . DA  B 1 5  ? 6.787  1.346   9.175   1.00 34.62  ? ? ? ? ? ? 17  DA  B C6    1
ATOM   328 N  N6    . DA  B 1 5  ? 5.538  1.734   9.450   1.00 40.59  ? ? ? ? ? ? 17  DA  B N6    1
ATOM   329 N  N1    . DA  B 1 5  ? 7.719  2.290   8.930   1.00 38.79  ? ? ? ? ? ? 17  DA  B N1    1
ATOM   330 C  C2    . DA  B 1 5  ? 8.969  1.901   8.648   1.00 40.00  ? ? ? ? ? ? 17  DA  B C2    1
ATOM   331 N  N3    . DA  B 1 5  ? 9.474  0.671   8.592   1.00 45.23  ? ? ? ? ? ? 17  DA  B N3    1
ATOM   332 C  C4    . DA  B 1 5  ? 8.526  -0.240  8.865   1.00 35.76  ? ? ? ? ? ? 17  DA  B C4    1
ATOM   333 P  P     . DA  B 1 6  ? 13.626 -4.397  9.688   1.00 60.35  ? ? ? ? ? ? 18  DA  B P     1
ATOM   334 O  OP1   . DA  B 1 6  ? 14.221 -5.214  8.602   1.00 63.82  ? ? ? ? ? ? 18  DA  B OP1   1
ATOM   335 O  OP2   . DA  B 1 6  ? 13.144 -5.035  10.931  1.00 49.85  ? ? ? ? ? ? 18  DA  B OP2   1
ATOM   336 O  'O5'' . DA  B 1 6  ? 14.622 -3.248  10.144  1.00 59.82  ? ? ? ? ? ? 18  DA  B 'O5'' 1
ATOM   337 C  'C5'' . DA  B 1 6  ? 15.222 -2.375  9.182   1.00 53.72  ? ? ? ? ? ? 18  DA  B 'C5'' 1
ATOM   338 C  'C4'' . DA  B 1 6  ? 15.373 -1.007  9.798   1.00 52.59  ? ? ? ? ? ? 18  DA  B 'C4'' 1
ATOM   339 O  'O4'' . DA  B 1 6  ? 14.075 -0.384  9.913   1.00 55.38  ? ? ? ? ? ? 18  DA  B 'O4'' 1
ATOM   340 C  'C3'' . DA  B 1 6  ? 15.965 -1.026  11.205  1.00 44.97  ? ? ? ? ? ? 18  DA  B 'C3'' 1
ATOM   341 O  'O3'' . DA  B 1 6  ? 17.114 -0.188  11.173  1.00 59.93  ? ? ? ? ? ? 18  DA  B 'O3'' 1
ATOM   342 C  'C2'' . DA  B 1 6  ? 14.828 -0.567  12.111  1.00 50.88  ? ? ? ? ? ? 18  DA  B 'C2'' 1
```

ATOM	343	C	'C1''	.	DA	B	1	6	?	13.928	0.218	11.180	1.00	45.59	?	?	?	?	?	?	18	DA	B	'C1''	1
ATOM	344	N	N9	.	DA	B	1	6	?	12.502	0.193	11.505	1.00	41.89	?	?	?	?	?	?	18	DA	B	N9	1
ATOM	345	C	C8	.	DA	B	1	6	?	11.681	-0.902	11.603	1.00	35.00	?	?	?	?	?	?	18	DA	B	C8	1
ATOM	346	N	N7	.	DA	B	1	6	?	10.436	-0.601	11.890	1.00	40.93	?	?	?	?	?	?	18	DA	B	N7	1
ATOM	347	C	C5	.	DA	B	1	6	?	10.425	0.789	11.911	1.00	39.79	?	?	?	?	?	?	18	DA	B	C5	1
ATOM	348	C	C6	.	DA	B	1	6	?	9.393	1.726	12.103	1.00	41.31	?	?	?	?	?	?	18	DA	B	C6	1
ATOM	349	N	N6	.	DA	B	1	6	?	8.115	1.390	12.303	1.00	35.05	?	?	?	?	?	?	18	DA	B	N6	1
ATOM	350	N	N1	.	DA	B	1	6	?	9.715	3.037	12.035	1.00	37.78	?	?	?	?	?	?	18	DA	B	N1	1
ATOM	351	C	C2	.	DA	B	1	6	?	10.985	3.371	11.805	1.00	39.76	?	?	?	?	?	?	18	DA	B	C2	1
ATOM	352	N	N3	.	DA	B	1	6	?	12.041	2.585	11.605	1.00	44.05	?	?	?	?	?	?	18	DA	B	N3	1
ATOM	353	C	C4	.	DA	B	1	6	?	11.682	1.290	11.641	1.00	39.60	?	?	?	?	?	?	18	DA	B	C4	1
ATOM	354	P	P	.	DT	B	1	7	?	18.150	-0.225	12.366	1.00	66.24	?	?	?	?	?	?	19	DT	B	P	1
ATOM	355	O	OP1	.	DT	B	1	7	?	19.404	0.414	11.889	1.00	73.70	?	?	?	?	?	?	19	DT	B	OP1	1
ATOM	356	O	OP2	.	DT	B	1	7	?	18.168	-1.604	12.915	1.00	73.00	?	?	?	?	?	?	19	DT	B	OP2	1
ATOM	357	O	'O5''	.	DT	B	1	7	?	17.480	0.772	13.403	1.00	63.34	?	?	?	?	?	?	19	DT	B	'O5''	1
ATOM	358	C	'C5''	.	DT	B	1	7	?	17.286	2.131	12.994	1.00	59.35	?	?	?	?	?	?	19	DT	B	'C5''	1
ATOM	359	C	'C4''	.	DT	B	1	7	?	16.414	2.851	13.989	1.00	52.77	?	?	?	?	?	?	19	DT	B	'C4''	1
ATOM	360	O	'O4''	.	DT	B	1	7	?	15.043	2.464	13.823	1.00	43.01	?	?	?	?	?	?	19	DT	B	'O4''	1
ATOM	361	C	'C3''	.	DT	B	1	7	?	16.741	2.544	15.448	1.00	50.15	?	?	?	?	?	?	19	DT	B	'C3''	1
ATOM	362	O	'O3''	.	DT	B	1	7	?	17.589	3.588	15.904	1.00	57.13	?	?	?	?	?	?	19	DT	B	'O3''	1
ATOM	363	C	'C2''	.	DT	B	1	7	?	15.385	2.585	16.142	1.00	47.40	?	?	?	?	?	?	19	DT	B	'C2''	1
ATOM	364	C	'C1''	.	DT	B	1	7	?	14.405	2.874	15.005	1.00	44.86	?	?	?	?	?	?	19	DT	B	'C1''	1
ATOM	365	N	N1	.	DT	B	1	7	?	13.128	2.183	15.087	1.00	41.99	?	?	?	?	?	?	19	DT	B	N1	1
ATOM	366	C	C2	.	DT	B	1	7	?	11.999	2.961	15.192	1.00	40.74	?	?	?	?	?	?	19	DT	B	C2	1
ATOM	367	O	O2	.	DT	B	1	7	?	12.029	4.180	15.200	1.00	45.09	?	?	?	?	?	?	19	DT	B	O2	1
ATOM	368	N	N3	.	DT	B	1	7	?	10.821	2.257	15.242	1.00	38.44	?	?	?	?	?	?	19	DT	B	N3	1
ATOM	369	C	C4	.	DT	B	1	7	?	10.670	0.889	15.267	1.00	39.65	?	?	?	?	?	?	19	DT	B	C4	1
ATOM	370	O	O4	.	DT	B	1	7	?	9.546	0.403	15.355	1.00	40.87	?	?	?	?	?	?	19	DT	B	O4	1
ATOM	371	C	C5	.	DT	B	1	7	?	11.901	0.129	15.196	1.00	37.60	?	?	?	?	?	?	19	DT	B	C5	1
ATOM	372	C	C7	.	DT	B	1	7	?	11.834	-1.365	15.237	1.00	38.18	?	?	?	?	?	?	19	DT	B	C7	1
ATOM	373	C	C6	.	DT	B	1	7	?	13.055	0.806	15.109	1.00	40.86	?	?	?	?	?	?	19	DT	B	C6	1
ATOM	374	P	P	.	DT	B	1	8	?	18.294	3.480	17.302	1.00	61.96	?	?	?	?	?	?	20	DT	B	P	1
ATOM	375	O	OP1	.	DT	B	1	8	?	19.496	4.353	17.267	1.00	72.95	?	?	?	?	?	?	20	DT	B	OP1	1
ATOM	376	O	OP2	.	DT	B	1	8	?	18.438	2.030	17.625	1.00	58.29	?	?	?	?	?	?	20	DT	B	OP2	1
ATOM	377	O	'O5''	.	DT	B	1	8	?	17.227	4.175	18.257	1.00	52.32	?	?	?	?	?	?	20	DT	B	'O5''	1
ATOM	378	C	'C5''	.	DT	B	1	8	?	16.699	5.464	17.926	1.00	47.16	?	?	?	?	?	?	20	DT	B	'C5''	1
ATOM	379	C	'C4''	.	DT	B	1	8	?	15.636	5.844	18.927	1.00	54.14	?	?	?	?	?	?	20	DT	B	'C4''	1

```
ATOM   380 O   'O4'' . DT  B 1 8  ? 14.406 5.175   18.571  1.00 51.72  ? ? ? ? ? ? 20  DT  B 'O4'' 1
ATOM   381 C   'C3'' . DT  B 1 8  ? 15.945 5.420   20.366  1.00 55.66  ? ? ? ? ? ? 20  DT  B 'C3'' 1
ATOM   382 O   'O3'' . DT  B 1 8  ? 15.647 6.479   21.276  1.00 62.72  ? ? ? ? ? ? 20  DT  B 'O3'' 1
ATOM   383 C   'C2'' . DT  B 1 8  ? 15.020 4.243   20.607  1.00 48.82  ? ? ? ? ? ? 20  DT  B 'C2'' 1
ATOM   384 C   'C1'' . DT  B 1 8  ? 13.848 4.604   19.724  1.00 48.53  ? ? ? ? ? ? 20  DT  B 'C1'' 1
ATOM   385 N   N1    . DT  B 1 8  ? 13.035 3.471   19.303  1.00 41.47  ? ? ? ? ? ? 20  DT  B N1    1
ATOM   386 C   C2    . DT  B 1 8  ? 11.693 3.702   19.147  1.00 42.38  ? ? ? ? ? ? 20  DT  B C2    1
ATOM   387 O   O2    . DT  B 1 8  ? 11.182 4.786   19.358  1.00 40.94  ? ? ? ? ? ? 20  DT  B O2    1
ATOM   388 N   N3    . DT  B 1 8  ? 10.961 2.597   18.790  1.00 39.48  ? ? ? ? ? ? 20  DT  B N3    1
ATOM   389 C   C4    . DT  B 1 8  ? 11.440 1.329   18.529  1.00 43.13  ? ? ? ? ? ? 20  DT  B C4    1
ATOM   390 O   O4    . DT  B 1 8  ? 10.665 0.442   18.187  1.00 43.85  ? ? ? ? ? ? 20  DT  B O4    1
ATOM   391 C   C5    . DT  B 1 8  ? 12.874 1.170   18.676  1.00 36.61  ? ? ? ? ? ? 20  DT  B C5    1
ATOM   392 C   C7    . DT  B 1 8  ? 13.491 -0.170  18.421  1.00 41.24  ? ? ? ? ? ? 20  DT  B C7    1
ATOM   393 C   C6    . DT  B 1 8  ? 13.588 2.240   19.037  1.00 31.95  ? ? ? ? ? ? 20  DT  B C6    1
ATOM   394 O   O1P   . D3N B 1 9  ? 17.527 5.577   22.677  1.00 57.63  ? ? ? ? ? ? 21  D3N B O1P   1
ATOM   395 P   P     . D3N B 1 9  ? 16.324 6.477   22.716  1.00 73.10  ? ? ? ? ? ? 21  D3N B P     1
ATOM   396 O   O2P   . D3N B 1 9  ? 16.449 7.907   23.055  1.00 71.06  ? ? ? ? ? ? 21  D3N B O2P   1
ATOM   397 O   'O5'' . D3N B 1 9  ? 15.191 5.843   23.617  1.00 65.54  ? ? ? ? ? ? 21  D3N B 'O5'' 1
ATOM   398 C   'C5'' . D3N B 1 9  ? 15.589 5.167   24.785  1.00 68.44  ? ? ? ? ? ? 21  D3N B 'C5'' 1
ATOM   399 C   'C4'' . D3N B 1 9  ? 14.459 5.106   25.773  1.00 63.43  ? ? ? ? ? ? 21  D3N B 'C4'' 1
ATOM   400 O   'O4'' . D3N B 1 9  ? 13.681 4.031   25.359  1.00 56.75  ? ? ? ? ? ? 21  D3N B 'O4'' 1
ATOM   401 C   'C3'' . D3N B 1 9  ? 14.935 4.721   27.123  1.00 63.88  ? ? ? ? ? ? 21  D3N B 'C3'' 1
ATOM   402 O   'O3'' . D3N B 1 9  ? 15.133 5.944   27.827  1.00 67.07  ? ? ? ? ? ? 21  D3N B 'O3'' 1
ATOM   403 C   'C2'' . D3N B 1 9  ? 13.814 3.859   27.696  1.00 56.92  ? ? ? ? ? ? 21  D3N B 'C2'' 1
ATOM   404 C   'C1'' . D3N B 1 9  ? 12.920 3.605   26.492  1.00 52.83  ? ? ? ? ? ? 21  D3N B 'C1'' 1
ATOM   405 N   N1    . D3N B 1 9  ? 12.484 2.276   26.132  1.00 47.27  ? ? ? ? ? ? 21  D3N B N1    1
ATOM   406 C   C2    . D3N B 1 9  ? 13.435 1.323   25.912  1.00 47.67  ? ? ? ? ? ? 21  D3N B C2    1
ATOM   407 O   O2    . D3N B 1 9  ? 14.627 1.553   26.095  1.00 53.65  ? ? ? ? ? ? 21  D3N B O2    1
ATOM   408 N   N3    . D3N B 1 9  ? 13.009 0.096   25.555  1.00 43.83  ? ? ? ? ? ? 21  D3N B N3    1
ATOM   409 C   C4    . D3N B 1 9  ? 11.663 -0.223  25.295  1.00 39.23  ? ? ? ? ? ? 21  D3N B C4    1
ATOM   410 C   C5    . D3N B 1 9  ? 10.696 0.762   25.488  1.00 44.29  ? ? ? ? ? ? 21  D3N B C5    1
ATOM   411 C   C6    . D3N B 1 9  ? 11.081 2.040   25.924  1.00 42.62  ? ? ? ? ? ? 21  D3N B C6    1
ATOM   412 C   C7    . D3N B 1 9  ? 10.111 2.990   26.145  1.00 52.13  ? ? ? ? ? ? 21  D3N B C7    1
ATOM   413 C   C8    . D3N B 1 9  ? 8.771  2.646   25.913  1.00 39.74  ? ? ? ? ? ? 21  D3N B C8    1
ATOM   414 C   C9    . D3N B 1 9  ? 8.378  1.426   25.500  1.00 47.35  ? ? ? ? ? ? 21  D3N B C9    1
ATOM   415 C   C10   . D3N B 1 9  ? 9.330  0.451   25.258  1.00 41.01  ? ? ? ? ? ? 21  D3N B C10   1
ATOM   416 C   C11   . D3N B 1 9  ? 8.992  -0.837  24.848  1.00 43.98  ? ? ? ? ? ? 21  D3N B C11   1
```

```
ATOM   417 C   C12    . D3N B 1 9  ? 9.952  -1.768  24.635  1.00 45.05  ? ? ? ? ? ? 21  D3N B C12    1
ATOM   418 C   C13    . D3N B 1 9  ? 11.312 -1.482  24.872  1.00 47.17  ? ? ? ? ? ? 21  D3N B C13    1
ATOM   419 P   P      . DG  B 1 10 ? 15.736 5.916   29.307  1.00 60.70  ? ? ? ? ? ? 22  DG  B P      1
ATOM   420 O   OP1    . DG  B 1 10 ? 16.433 7.208   29.532  1.00 63.59  ? ? ? ? ? ? 22  DG  B OP1    1
ATOM   421 O   OP2    . DG  B 1 10 ? 16.495 4.640   29.465  1.00 51.49  ? ? ? ? ? ? 22  DG  B OP2    1
ATOM   422 O   'O5''  . DG  B 1 10 ? 14.420 5.963   30.208  1.00 56.71  ? ? ? ? ? ? 22  DG  B 'O5'' 1
ATOM   423 C   'C5''  . DG  B 1 10 ? 13.283 6.789   29.854  1.00 44.99  ? ? ? ? ? ? 22  DG  B 'C5'' 1
ATOM   424 C   'C4''  . DG  B 1 10 ? 12.025 6.262   30.509  1.00 50.00  ? ? ? ? ? ? 22  DG  B 'C4'' 1
ATOM   425 O   'O4''  . DG  B 1 10 ? 11.708 4.952   29.984  1.00 42.89  ? ? ? ? ? ? 22  DG  B 'O4'' 1
ATOM   426 C   'C3''  . DG  B 1 10 ? 12.105 6.068   32.024  1.00 48.07  ? ? ? ? ? ? 22  DG  B 'C3'' 1
ATOM   427 O   'O3''  . DG  B 1 10 ? 10.760 6.110   32.488  1.00 50.84  ? ? ? ? ? ? 22  DG  B 'O3'' 1
ATOM   428 C   'C2''  . DG  B 1 10 ? 12.570 4.634   32.149  1.00 41.22  ? ? ? ? ? ? 22  DG  B 'C2'' 1
ATOM   429 C   'C1''  . DG  B 1 10 ? 11.752 3.990   31.034  1.00 41.10  ? ? ? ? ? ? 22  DG  B 'C1'' 1
ATOM   430 N   N9     . DG  B 1 10 ? 12.282 2.747   30.485  1.00 35.88  ? ? ? ? ? ? 22  DG  B N9     1
ATOM   431 C   C8     . DG  B 1 10 ? 13.594 2.339   30.459  1.00 41.27  ? ? ? ? ? ? 22  DG  B C8     1
ATOM   432 N   N7     . DG  B 1 10 ? 13.753 1.160   29.926  1.00 35.66  ? ? ? ? ? ? 22  DG  B N7     1
ATOM   433 C   C5     . DG  B 1 10 ? 12.468 0.765   29.576  1.00 38.68  ? ? ? ? ? ? 22  DG  B C5     1
ATOM   434 C   C6     . DG  B 1 10 ? 12.009 -0.426  28.970  1.00 36.57  ? ? ? ? ? ? 22  DG  B C6     1
ATOM   435 O   O6     . DG  B 1 10 ? 12.660 -1.408  28.615  1.00 38.62  ? ? ? ? ? ? 22  DG  B O6     1
ATOM   436 N   N1     . DG  B 1 10 ? 10.636 -0.396  28.759  1.00 32.06  ? ? ? ? ? ? 22  DG  B N1     1
ATOM   437 C   C2     . DG  B 1 10 ? 9.801  0.614   29.146  1.00 37.35  ? ? ? ? ? ? 22  DG  B C2     1
ATOM   438 N   N2     . DG  B 1 10 ? 8.497  0.408   28.941  1.00 33.23  ? ? ? ? ? ? 22  DG  B N2     1
ATOM   439 N   N3     . DG  B 1 10 ? 10.210 1.716   29.751  1.00 37.31  ? ? ? ? ? ? 22  DG  B N3     1
ATOM   440 C   C4     . DG  B 1 10 ? 11.553 1.741   29.897  1.00 35.81  ? ? ? ? ? ? 22  DG  B C4     1
ATOM   441 P   P      . DC  B 1 11 ? 10.442 6.622   33.947  1.00 54.63  ? ? ? ? ? ? 23  DC  B P      1
ATOM   442 O   OP1    . DC  B 1 11 ? 10.081 8.062   33.835  1.00 72.54  ? ? ? ? ? ? 23  DC  B OP1    1
ATOM   443 O   OP2    . DC  B 1 11 ? 11.572 6.234   34.816  1.00 46.04  ? ? ? ? ? ? 23  DC  B OP2    1
ATOM   444 O   'O5''  . DC  B 1 11 ? 9.071  5.874   34.286  1.00 50.89  ? ? ? ? ? ? 23  DC  B 'O5'' 1
ATOM   445 C   'C5''  . DC  B 1 11 ? 7.872  6.214   33.569  1.00 44.12  ? ? ? ? ? ? 23  DC  B 'C5'' 1
ATOM   446 C   'C4''  . DC  B 1 11 ? 6.937  5.029   33.482  1.00 43.81  ? ? ? ? ? ? 23  DC  B 'C4'' 1
ATOM   447 O   'O4''  . DC  B 1 11 ? 7.576  3.958   32.740  1.00 42.06  ? ? ? ? ? ? 23  DC  B 'O4'' 1
ATOM   448 C   'C3''  . DC  B 1 11 ? 6.528  4.432   34.832  1.00 38.81  ? ? ? ? ? ? 23  DC  B 'C3'' 1
ATOM   449 O   'O3''  . DC  B 1 11 ? 5.132  4.149   34.847  1.00 45.37  ? ? ? ? ? ? 23  DC  B 'O3'' 1
ATOM   450 C   'C2''  . DC  B 1 11 ? 7.300  3.124   34.901  1.00 40.79  ? ? ? ? ? ? 23  DC  B 'C2'' 1
ATOM   451 C   'C1''  . DC  B 1 11 ? 7.379  2.736   33.434  1.00 33.88  ? ? ? ? ? ? 23  DC  B 'C1'' 1
ATOM   452 N   N1     . DC  B 1 11 ? 8.504  1.846   33.120  1.00 36.40  ? ? ? ? ? ? 23  DC  B N1     1
ATOM   453 C   C2     . DC  B 1 11 ? 8.272  0.685   32.369  1.00 32.79  ? ? ? ? ? ? 23  DC  B C2     1
```

```
ATOM   454 O  O2    . DC  B 1 11 ? 7.131  0.479  31.908 1.00 36.07 ? ? ? ? ? ? 23  DC  B O2    1
ATOM   455 N  N3    . DC  B 1 11 ? 9.293  -0.184 32.167 1.00 30.32 ? ? ? ? ? ? 23  DC  B N3    1
ATOM   456 C  C4    . DC  B 1 11 ? 10.503 0.081  32.671 1.00 32.96 ? ? ? ? ? ? 23  DC  B C4    1
ATOM   457 N  N4    . DC  B 1 11 ? 11.472 -0.815 32.470 1.00 32.29 ? ? ? ? ? ? 23  DC  B N4    1
ATOM   458 C  C5    . DC  B 1 11 ? 10.777 1.286  33.385 1.00 32.52 ? ? ? ? ? ? 23  DC  B C5    1
ATOM   459 C  C6    . DC  B 1 11 ? 9.753  2.117  33.606 1.00 37.44 ? ? ? ? ? ? 23  DC  B C6    1
ATOM   460 P  P     . DG  B 1 12 ? 4.277  4.492  36.173 1.00 52.39 ? ? ? ? ? ? 24  DG  B P     1
ATOM   461 O  OP1   . DG  B 1 12 ? 2.829  4.210  35.898 1.00 51.58 ? ? ? ? ? ? 24  DG  B OP1   1
ATOM   462 O  OP2   . DG  B 1 12 ? 4.739  5.811  36.678 1.00 48.36 ? ? ? ? ? ? 24  DG  B OP2   1
ATOM   463 O  'O5'' . DG  B 1 12 ? 4.756  3.384  37.209 1.00 46.27 ? ? ? ? ? ? 24  DG  B 'O5'' 1
ATOM   464 C  'C5'' . DG  B 1 12 ? 4.451  2.002  36.974 1.00 44.58 ? ? ? ? ? ? 24  DG  B 'C5'' 1
ATOM   465 C  'C4'' . DG  B 1 12 ? 5.053  1.193  38.090 1.00 39.93 ? ? ? ? ? ? 24  DG  B 'C4'' 1
ATOM   466 O  'O4'' . DG  B 1 12 ? 6.494  1.319  38.004 1.00 36.23 ? ? ? ? ? ? 24  DG  B 'O4'' 1
ATOM   467 C  'C3'' . DG  B 1 12 ? 4.673  1.705  39.476 1.00 41.18 ? ? ? ? ? ? 24  DG  B 'C3'' 1
ATOM   468 O  'O3'' . DG  B 1 12 ? 3.540  0.965  39.948 1.00 45.43 ? ? ? ? ? ? 24  DG  B 'O3'' 1
ATOM   469 C  'C2'' . DG  B 1 12 ? 5.937  1.493  40.281 1.00 44.61 ? ? ? ? ? ? 24  DG  B 'C2'' 1
ATOM   470 C  'C1'' . DG  B 1 12 ? 7.030  1.711  39.250 1.00 39.64 ? ? ? ? ? ? 24  DG  B 'C1'' 1
ATOM   471 N  N9    . DG  B 1 12 ? 7.442  3.094  39.113 1.00 44.84 ? ? ? ? ? ? 24  DG  B N9    1
ATOM   472 C  C8    . DG  B 1 12 ? 6.712  4.200  39.471 1.00 50.34 ? ? ? ? ? ? 24  DG  B C8    1
ATOM   473 N  N7    . DG  B 1 12 ? 7.292  5.323  39.143 1.00 46.30 ? ? ? ? ? ? 24  DG  B N7    1
ATOM   474 C  C5    . DG  B 1 12 ? 8.422  4.933  38.443 1.00 46.08 ? ? ? ? ? ? 24  DG  B C5    1
ATOM   475 C  C6    . DG  B 1 12 ? 9.446  5.712  37.846 1.00 53.78 ? ? ? ? ? ? 24  DG  B C6    1
ATOM   476 O  O6    . DG  B 1 12 ? 9.530  6.948  37.769 1.00 50.57 ? ? ? ? ? ? 24  DG  B O6    1
ATOM   477 N  N1    . DG  B 1 12 ? 10.419 4.908  37.254 1.00 42.62 ? ? ? ? ? ? 24  DG  B N1    1
ATOM   478 C  C2    . DG  B 1 12 ? 10.434 3.531  37.278 1.00 42.89 ? ? ? ? ? ? 24  DG  B C2    1
ATOM   479 N  N2    . DG  B 1 12 ? 11.496 2.933  36.700 1.00 37.64 ? ? ? ? ? ? 24  DG  B N2    1
ATOM   480 N  N3    . DG  B 1 12 ? 9.495  2.796  37.851 1.00 42.44 ? ? ? ? ? ? 24  DG  B N3    1
ATOM   481 C  C4    . DG  B 1 12 ? 8.524  3.556  38.405 1.00 45.36 ? ? ? ? ? ? 24  DG  B C4    1
HETATM 482 N  N1    . SPM C 2 .  ? 15.307 -2.224 30.707 0.50 45.16 ? ? ? ? ? ? 101 SPM A N1    1
HETATM 483 C  C2    . SPM C 2 .  ? 14.648 -3.505 30.906 1.00 50.52 ? ? ? ? ? ? 101 SPM A C2    1
HETATM 484 C  C3    . SPM C 2 .  ? 14.177 -3.681 32.355 1.00 49.96 ? ? ? ? ? ? 101 SPM A C3    1
HETATM 485 C  C4    . SPM C 2 .  ? 13.198 -4.830 32.413 1.00 46.18 ? ? ? ? ? ? 101 SPM A C4    1
HETATM 486 N  N5    . SPM C 2 .  ? 12.876 -5.334 33.745 1.00 49.66 ? ? ? ? ? ? 101 SPM A N5    1
HETATM 487 C  C6    . SPM C 2 .  ? 11.836 -6.363 33.800 1.00 40.82 ? ? ? ? ? ? 101 SPM A C6    1
HETATM 488 C  C7    . SPM C 2 .  ? 11.700 -6.958 35.173 1.00 44.69 ? ? ? ? ? ? 101 SPM A C7    1
HETATM 489 C  C8    . SPM C 2 .  ? 10.292 -7.489 35.304 1.00 45.41 ? ? ? ? ? ? 101 SPM A C8    1
HETATM 490 C  C9    . SPM C 2 .  ? 10.370 -8.593 36.328 1.00 39.65 ? ? ? ? ? ? 101 SPM A C9    1
```

```
HETATM 491 N  N10  . SPM C 2 . ? 9.328  -8.480  37.308  1.00 48.73 ? ? ? ? ? ? 101 SPM A N10 1
HETATM 492 C  C11  . SPM C 2 . ? 8.275  -9.514  37.339  1.00 42.36 ? ? ? ? ? ? 101 SPM A C11 1
HETATM 493 C  C12  . SPM C 2 . ? 8.417  -10.350 38.617  1.00 40.48 ? ? ? ? ? ? 101 SPM A C12 1
HETATM 494 C  C13  . SPM C 2 . ? 7.395  -11.529 38.711  1.00 36.10 ? ? ? ? ? ? 101 SPM A C13 1
HETATM 495 N  N14  . SPM C 2 . ? 7.319  -12.113 37.413  1.00 26.41 ? ? ? ? ? ? 101 SPM A N14 1
HETATM 496 SR SR   . SR  D 3 . ? 15.511 4.253   -10.211 1.00 80.43 ? ? ? ? ? ? 102 SR  A SR  1
HETATM 497 O  O    . HOH E 4 . ? 5.745  10.707  7.973   1.00 50.57 ? ? ? ? ? ? 201 HOH A O   1
HETATM 498 O  O    . HOH E 4 . ? 3.981  2.092   12.526  1.00 55.74 ? ? ? ? ? ? 202 HOH A O   1
HETATM 499 O  O    . HOH E 4 . ? 5.535  7.189   -5.997  1.00 37.30 ? ? ? ? ? ? 203 HOH A O   1
HETATM 500 O  O    . HOH E 4 . ? 5.857  10.607  2.957   1.00 39.96 ? ? ? ? ? ? 204 HOH A O   1
HETATM 501 O  O    . HOH E 4 . ? 16.122 -0.073  -7.306  1.00 76.18 ? ? ? ? ? ? 205 HOH A O   1
HETATM 502 O  O    . HOH E 4 . ? 12.456 -9.842  29.587  0.50 39.91 ? ? ? ? ? ? 206 HOH A O   1
HETATM 503 O  O    . HOH E 4 . ? 15.126 0.991   -11.310 1.00 64.27 ? ? ? ? ? ? 207 HOH A O   1
HETATM 504 O  O    . HOH E 4 . ? 11.936 -2.931  -12.948 1.00 43.14 ? ? ? ? ? ? 208 HOH A O   1
HETATM 505 O  O    . HOH E 4 . ? 16.727 -3.172  -8.920  1.00 68.21 ? ? ? ? ? ? 209 HOH A O   1
HETATM 506 O  O    . HOH E 4 . ? 13.771 10.608  -3.551  0.50 32.95 ? ? ? ? ? ? 210 HOH A O   1
HETATM 507 O  O    . HOH E 4 . ? 9.441  1.770   -2.007  1.00 36.92 ? ? ? ? ? ? 211 HOH A O   1
HETATM 508 O  O    . HOH E 4 . ? 9.916  -4.279  -14.180 1.00 39.00 ? ? ? ? ? ? 212 HOH A O   1
HETATM 509 O  O    . HOH E 4 . ? 5.425  -5.959  -10.092 1.00 56.75 ? ? ? ? ? ? 213 HOH A O   1
HETATM 510 O  O    . HOH E 4 . ? 6.736  11.394  5.248   0.50 34.77 ? ? ? ? ? ? 214 HOH A O   1
HETATM 511 O  O    . HOH E 4 . ? 11.867 -15.768 30.801  1.00 47.55 ? ? ? ? ? ? 215 HOH A O   1
HETATM 512 O  O    . HOH E 4 . ? 14.392 -5.303  36.717  0.50 36.19 ? ? ? ? ? ? 216 HOH A O   1
HETATM 513 O  O    . HOH E 4 . ? 3.012  -10.254 22.862  1.00 67.16 ? ? ? ? ? ? 217 HOH A O   1
HETATM 514 O  O    . HOH E 4 . ? 13.213 9.047   -5.011  0.50 43.01 ? ? ? ? ? ? 218 HOH A O   1
HETATM 515 O  O    . HOH E 4 . ? -2.568 -2.294  17.282  0.50 44.49 ? ? ? ? ? ? 219 HOH A O   1
HETATM 516 O  O    . HOH E 4 . ? 10.725 -14.748 28.779  0.50 39.46 ? ? ? ? ? ? 220 HOH A O   1
HETATM 517 O  O    . HOH E 4 . ? 15.703 9.836   -2.496  0.50 31.11 ? ? ? ? ? ? 221 HOH A O   1
HETATM 518 O  O    . HOH E 4 . ? 9.346  8.423   16.634  1.00 52.47 ? ? ? ? ? ? 222 HOH A O   1
HETATM 519 O  O    . HOH E 4 . ? 11.510 1.193   -0.164  0.50 40.92 ? ? ? ? ? ? 223 HOH A O   1
HETATM 520 O  O    . HOH E 4 . ? 12.332 12.804  -0.232  1.00 59.02 ? ? ? ? ? ? 224 HOH A O   1
HETATM 521 O  O    . HOH E 4 . ? 14.811 -18.153 31.387  1.00 54.50 ? ? ? ? ? ? 225 HOH A O   1
HETATM 522 O  O    . HOH E 4 . ? 12.781 -17.856 31.806  1.00 47.82 ? ? ? ? ? ? 226 HOH A O   1
HETATM 523 O  O    . HOH E 4 . ? 1.224  6.114   15.685  1.00 60.25 ? ? ? ? ? ? 227 HOH A O   1
HETATM 524 O  O    . HOH E 4 . ? 10.603 6.919   -7.970  1.00 37.70 ? ? ? ? ? ? 228 HOH A O   1
HETATM 525 O  O    . HOH F 4 . ? -0.922 0.065   -5.369  1.00 42.62 ? ? ? ? ? ? 101 HOH B O   1
HETATM 526 O  O    . HOH F 4 . ? 8.026  9.832   35.707  1.00 68.74 ? ? ? ? ? ? 102 HOH B O   1
HETATM 527 O  O    . HOH F 4 . ? 15.975 -1.008  15.507  1.00 52.27 ? ? ? ? ? ? 103 HOH B O   1
```

```
HETATM 528 O   O     . HOH F 4 .  ? 14.793 -2.822  15.169  1.00 58.11  ? ? ? ? ? ? 104 HOH B O     1
HETATM 529 O   O     . HOH F 4 .  ? -0.287 5.138   4.841   1.00 62.89  ? ? ? ? ? ? 105 HOH B O     1
HETATM 530 O   O     . HOH F 4 .  ? 2.869  1.485   6.566   1.00 45.01  ? ? ? ? ? ? 106 HOH B O     1
HETATM 531 O   O     . HOH F 4 .  ? 2.751  0.192   9.510   0.50 39.34  ? ? ? ? ? ? 107 HOH B O     1
HETATM 532 O   O     . HOH F 4 .  ? 17.774 2.975   22.558  1.00 57.77  ? ? ? ? ? ? 108 HOH B O     1
HETATM 533 O   O     . HOH F 4 .  ? 16.850 0.536   28.081  1.00 66.55  ? ? ? ? ? ? 109 HOH B O     1
HETATM 534 O   O     . HOH F 4 .  ? 8.401  -1.858  12.571  0.50 37.14  ? ? ? ? ? ? 110 HOH B O     1
HETATM 535 O   O     . HOH F 4 .  ? 5.873  -5.073  9.869   1.00 47.64  ? ? ? ? ? ? 111 HOH B O     1
HETATM 536 O   O     . HOH F 4 .  ? 6.830  8.002   40.323  1.00 55.54  ? ? ? ? ? ? 112 HOH B O     1
HETATM 537 O   O     . HOH F 4 .  ? 12.523 -2.219  4.994   1.00 56.73  ? ? ? ? ? ? 113 HOH B O     1
HETATM 538 O   O     . HOH F 4 .  ? 8.144  3.642   30.083  1.00 38.91  ? ? ? ? ? ? 114 HOH B O     1
HETATM 539 O   O     . HOH F 4 .  ? 10.239 -1.894  19.211  1.00 41.32  ? ? ? ? ? ? 115 HOH B O     1
HETATM 540 O   O     . HOH F 4 .  ? 11.394 6.208   23.097  1.00 61.62  ? ? ? ? ? ? 116 HOH B O     1
HETATM 541 O   O     . HOH F 4 .  ? -8.777 1.487   -2.112  1.00 70.13  ? ? ? ? ? ? 117 HOH B O     1
HETATM 542 O   O     . HOH F 4 .  ? -1.820 0.663   3.075   1.00 52.05  ? ? ? ? ? ? 118 HOH B O     1
HETATM 543 O   O     . HOH F 4 .  ? 21.214 2.225   19.730  1.00 65.35  ? ? ? ? ? ? 119 HOH B O     1
HETATM 544 O   O     . HOH F 4 .  ? 16.869 1.303   20.043  1.00 54.01  ? ? ? ? ? ? 120 HOH B O     1
HETATM 545 O   O     . HOH F 4 .  ? -2.810 -4.632  7.767   1.00 63.68  ? ? ? ? ? ? 121 HOH B O     1
#
```

Table S2. Crystal and Data Collection Statistics for the First Crystal. It was used to obtain phases for the DDD-XY duplex.

Space group	Orthorhombic $P2_12_12_1$
Cell parameters (Å)	a=26.4, b=37.3, c=77.6
Temperature of data collection (° C)	-170
Wavelength (Å)	1.605
Min resolution (Å)	26.9
Max resolution (Å)	1.95
Unique reflections (observed)	5861
Completeness all(%)/1.98-1.95 Å	99.0/98.0
I/σ (I) all/1.98-1.95 Å	20.3/4.34
R_{merge} all/1.98-1.95 Å	0.08/0.63
R_{work}	0.36
R_{free}	0.39
Number of DNA atoms	427
Number of water molecules	14
Number of ions	4 Ba^{2+}

Table S3. Crystal Data, Data Collection, and Refinement Statistics for the Second Crystal. It was used to obtain the final structure of the DDD-XY duplex.

Space group	Orthorhombic $P2_12_12_1$
Cell parameters (Å)	a=26.38, b=36.77, c=77.65
Temperature of data collection (° C)	-170
Wavelength (Å)	0.97857
Min resolution (Å)	30.0
Max resolution (Å)	1.7
Unique reflections (all)	8811
Unique reflections (observed)	8236
Completeness all/1.76-1.70 Å (%)	93.4/61.1
Redundancy all/1.76-1.70 Å	6.4/4.0
I/σ (I) all/1.76-1.70 Å	52.3/5.65
R_{merge} all/1.76-1.70 Å	0.04/0.23
R_{work}	0.26
R_{free}	0.30
Number of DNA atoms	481
Number of water molecules	49
Number of ions	1 Sr^{2+}
r.m.s. distances (Å)	0.01
r.m.s. angles (°)	1.6

Table S4. NMR Restraints Used for the DDD-GY Structure Calculations and Refinement Statistics.

NMR restraints	
NOE restraints	
Internucleotide	75
Intranucleotide	78
Total	153
Backbone torsion angle restraints	100
H-bonding restraints	50
Deoxyribose restraints	18
Total number of restraints	321
Refinement Statistics	
Number of distance restraint violations	25
Number of torsion restraint violations	14
Total distance penalty/Maximum penalty [kcal mol^{-1}]	2.60/0.46
Total torsion penalty/Maximum penalty [kcal mol^{-1}]	1.24/0.36
r.m.s. distances (Å)	0.012
r.m.s. angles (°)	2.4
Distance restraint force field [kcal mol^{-1} $Å^{-2}$]	32
Torsion restraint force field [kcal mol^{-1} deg^{-2}]	32

Table S5. Structural Statistics for the DDD-GY Duplex.

Average structure (obtained from 9 structures)			
RMS pairwise difference between structures		0.71	
RMS difference from average structure		0.47	
CORMA analysis for average structure[a]			
	Intranucleotide	Internucleotide	Total
Rx[b]	0.10	0.10	0.10
Average error[c]			0.02

[a] The mixing time was 250 ms. [b] Rx is 6th root R factor: $\Sigma[((Io)_i^{1/6})-((Ic)_i^{1/6})/\Sigma((Io)_i^{1/6})]$. [c] Average error: $\Sigma(Ic-Io)/n$, where Ic are NOE intensities calculated from refined structure, Io are experimental NOE intensities.

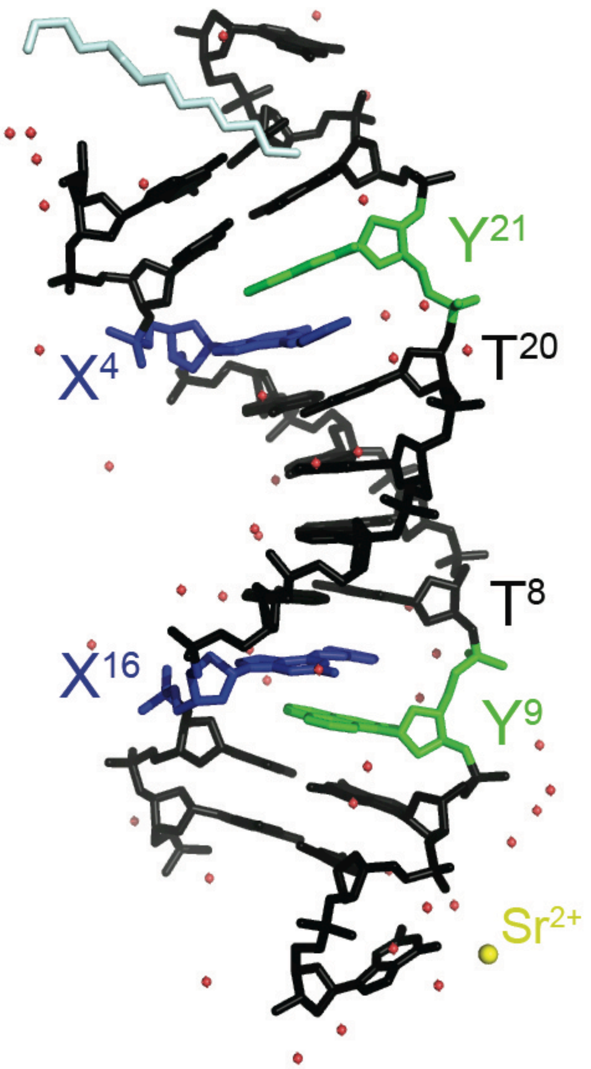

Figure S1. Structure of the DDD-XY duplex with water molecules (red spheres) and Sr^{2+} ion (yellow sphere) and spermine molecule (light blue). The benzyl groups of *O*^{6}-Bn-dG (shown in blue) intercalate between the thymine and dPer bases (shown in green) from the opposite strand. The dPer bases are in the *syn* conformation about the glycosyl bond. Electron density for bases C^1 and C^{13} was not visible. Bases G^{12} and G^{24} flipped out from the duplex. The intercalated structures unwind the duplex as compared to the unmodified DDD.

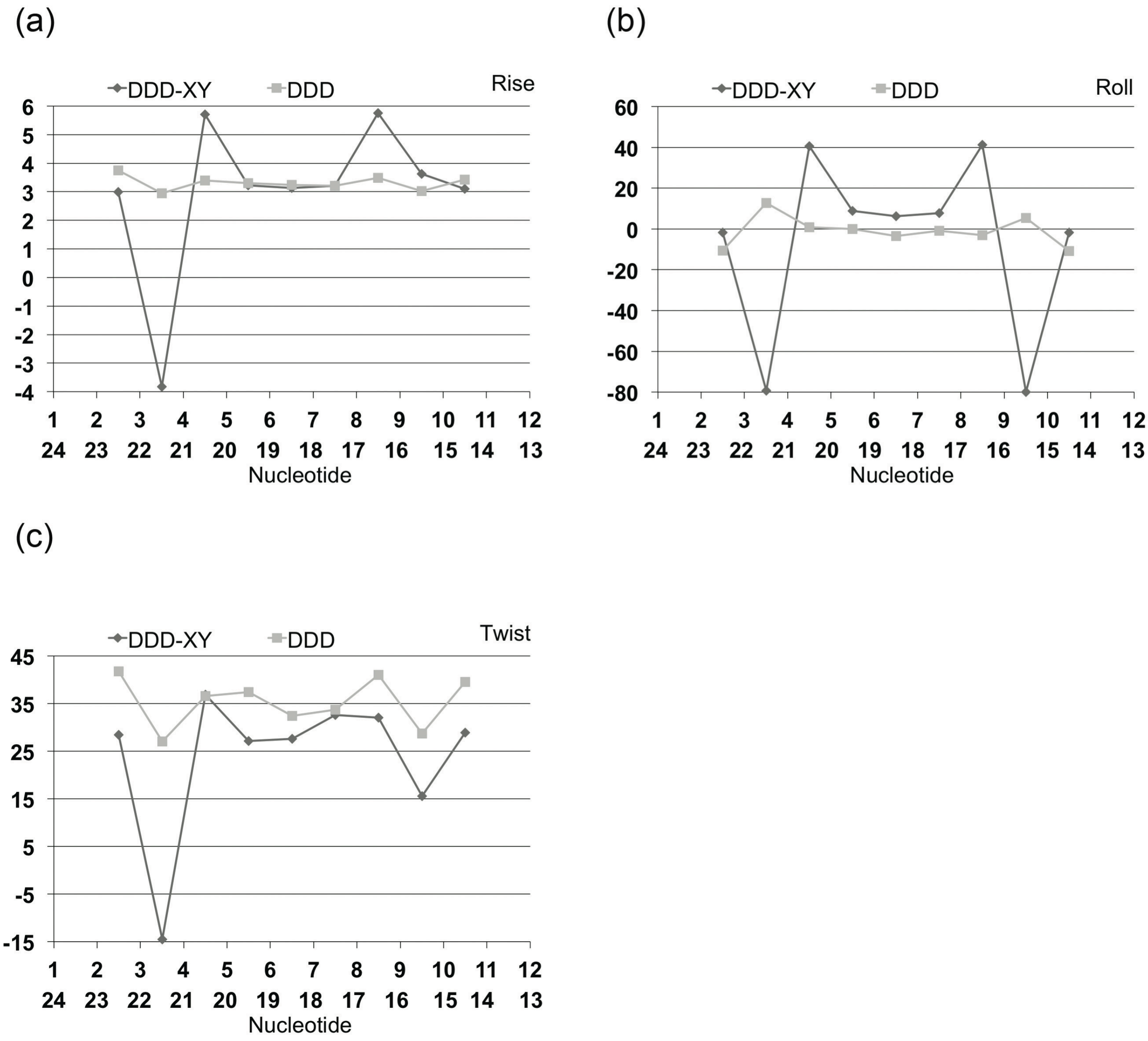

Figure S2. Interbase pair parameters: (a) helical rise, (b) roll and (c) twist for the DDD-XY, DDD (PDB entry 355D) duplexes.

(a)

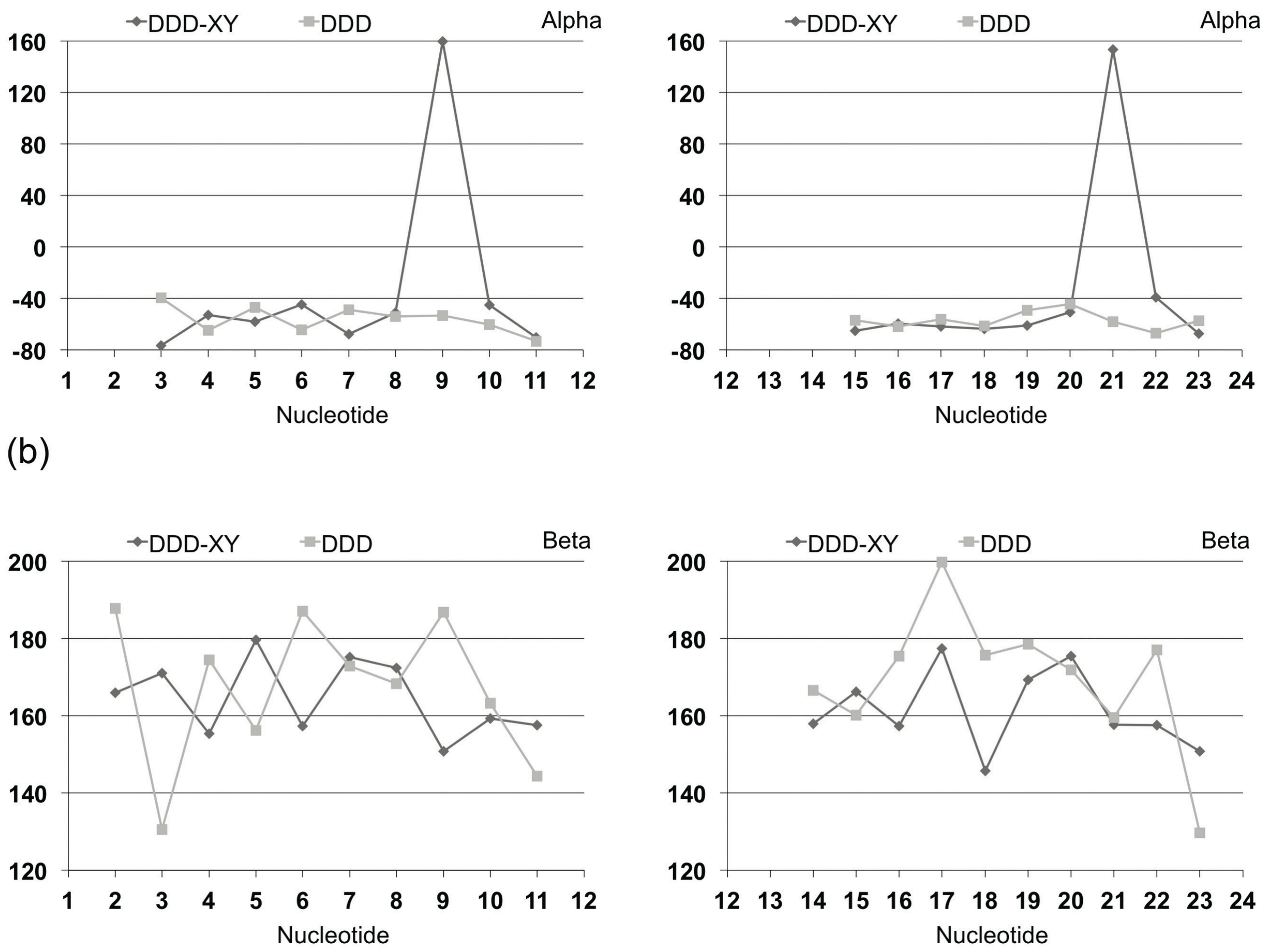

Figure S3. Comparison of backbone torsion angles (a) α and (b) β in the crystal structures of the DDD-XY, DDD (PDB entry 355D) duplexes.

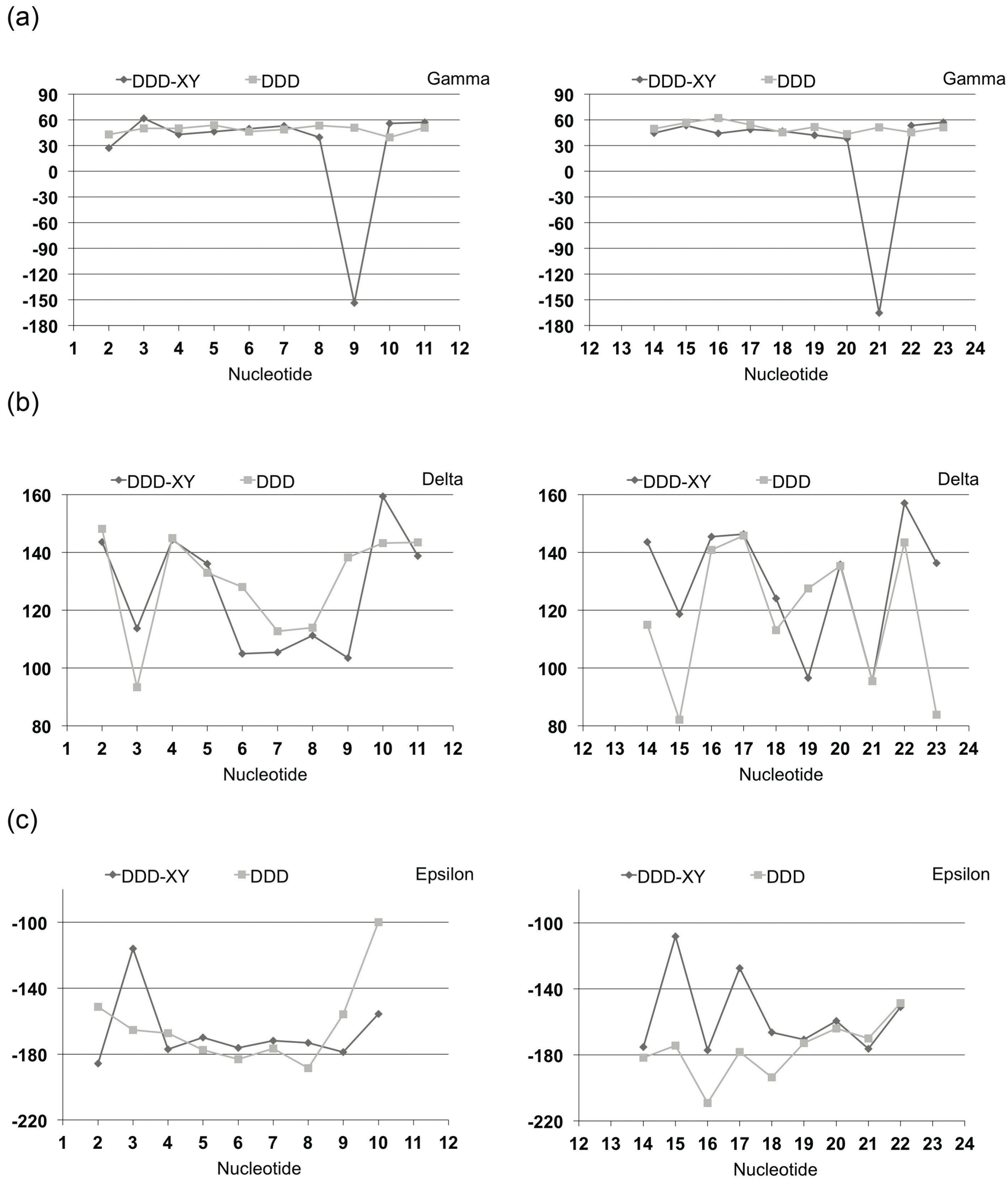

Figure S4. Comparison of (a) γ, (b) δ, (c) ε angles in the crystal structures of the DDD-XY, DDD (PDB entry 355D) duplexes.

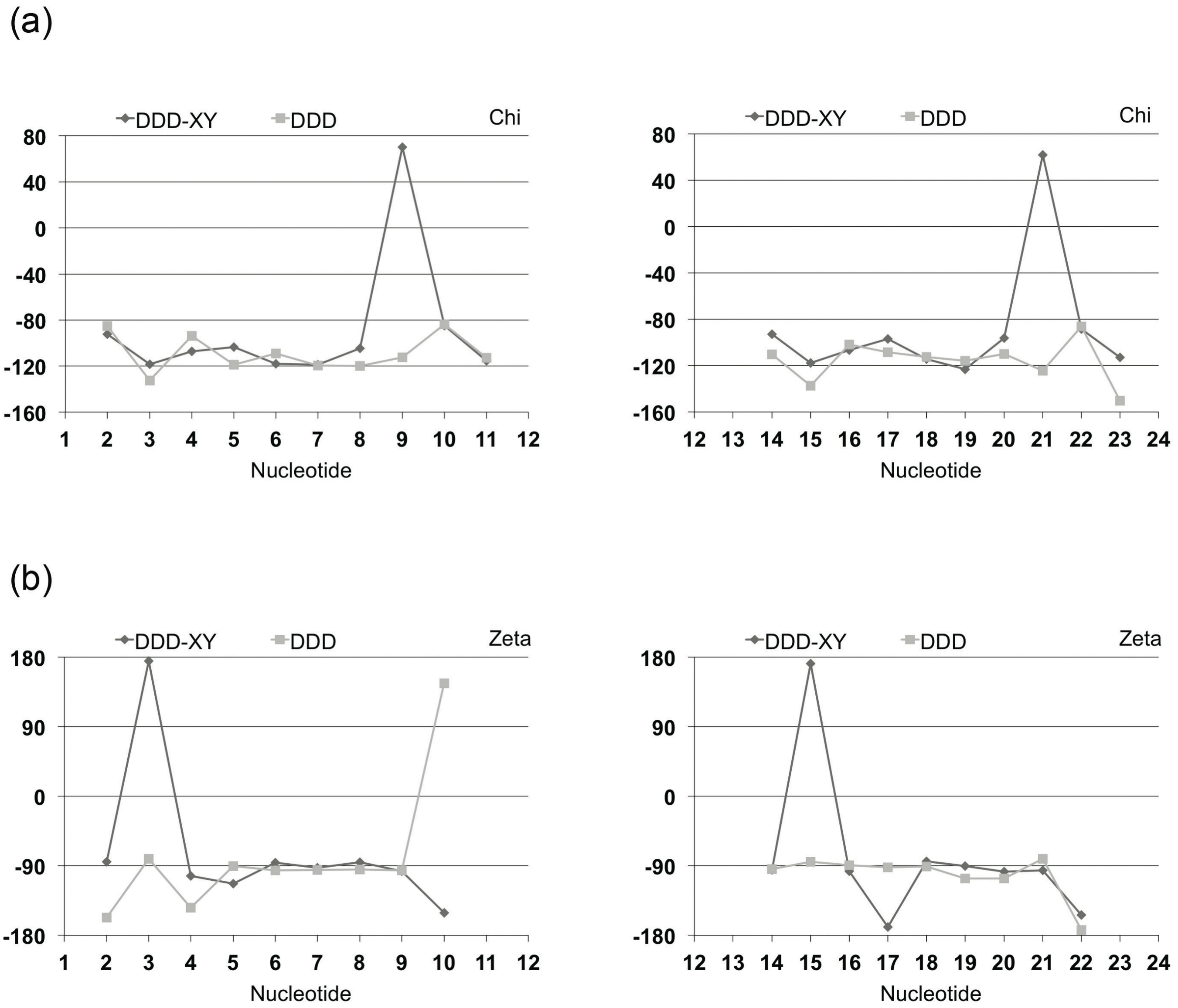

Figure S5. Comparison of (a) χ and (b) ζ angles in the crystal structures of the DDD-XY, DDD (PDB entry 355D) duplexes.

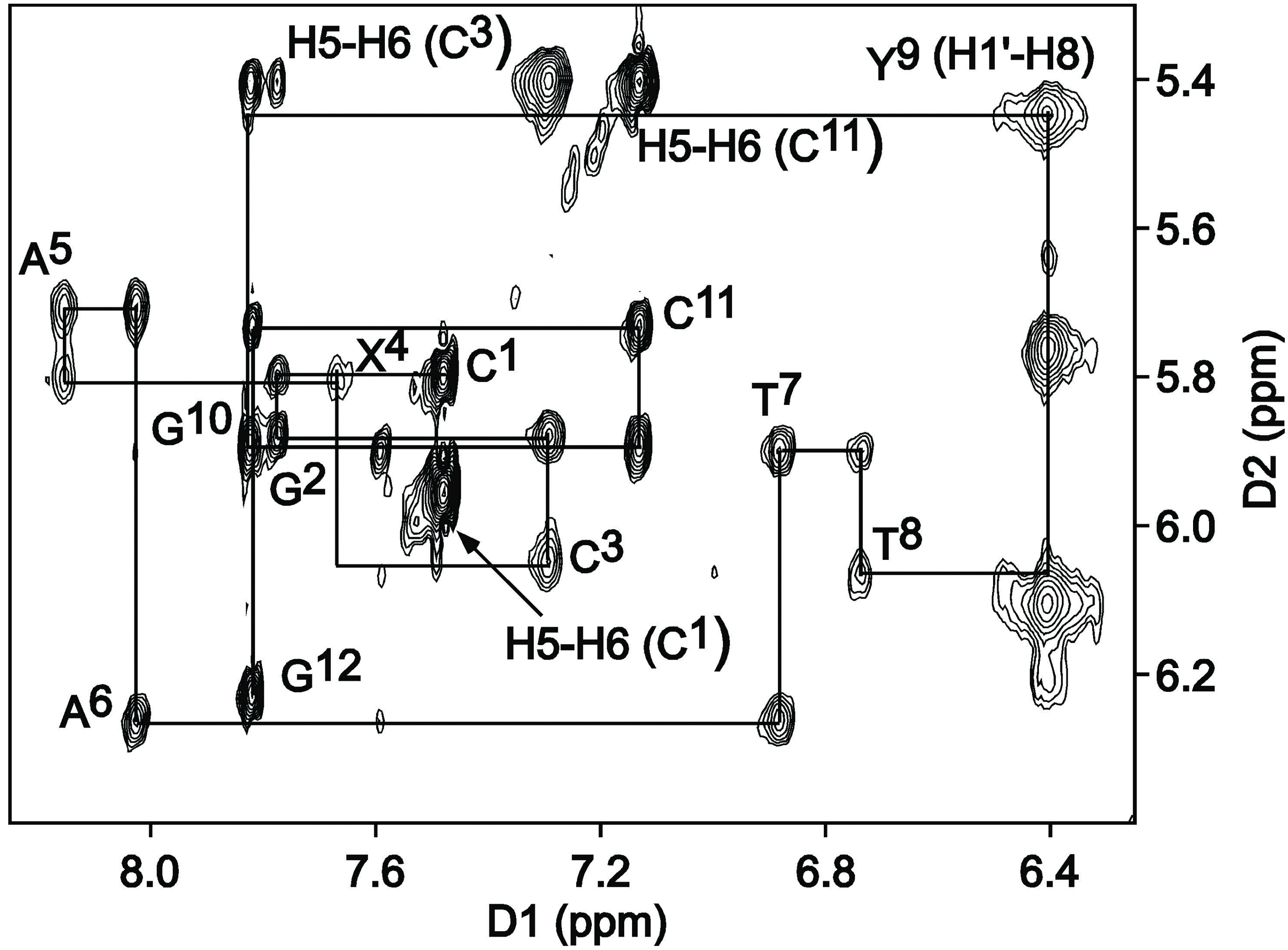

Figure S6. NOESY spectrum of the DDD-XY duplex showing sequential NOEs between the aromatic and anomeric protons from C^1 to G^{12}. The spectrum was collected at 10 °C at 900 MHz with 250 ms mixing time.

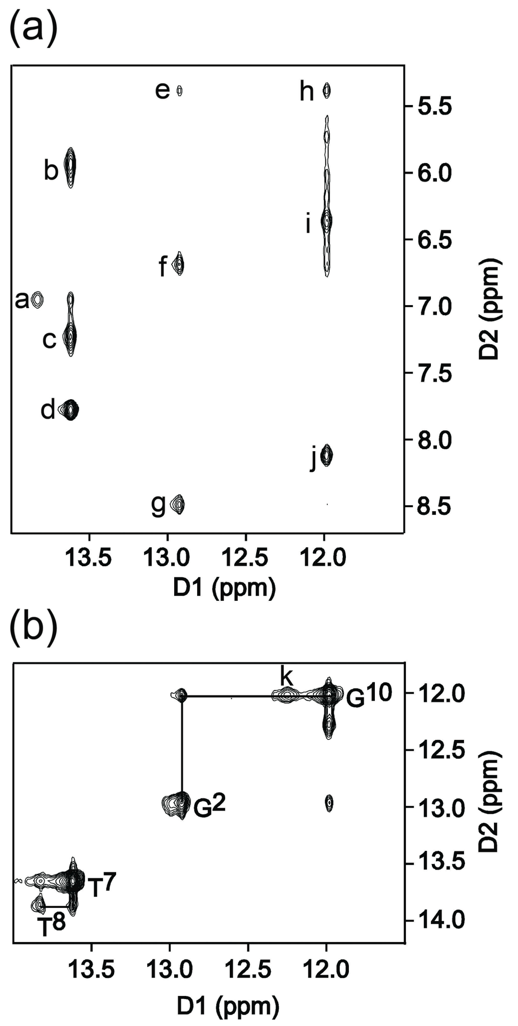

Figure S7. (a) Interstrand NOE cross peaks between complementary bases for the DDD-XY duplex: a, A^5 H2→T^8 N3H; b, A^5 N^6H2→T7 N3H; c, A^6 N^2H2→T^7 N3H; d, A^6 H2→T^7 N3H; e, C^{11} H5→G^2 N1H; f, C^{11} N^2H1→G^2 N1H; g, C^{11} N^2H2→G^2 N1H; h, C^3 H5→G^{10} N1H; i, C^3 N^2H1→G^{10} N1H; j, C^3 N^2H2→G^{10} N1H. (b) NOE connectivity of DDD-XY duplex, for the imino protons for the base pairs G^2:C^{11}, C^3:G^{10}, X^4:Y^9, A^5:T^8, A^5:T^7. Cross peaks between T^8 N3H→T^7 N3H and G^2 N1H→G^{10} N1H were present. There is no cross-peak between T^8→Y^9 and Y^9→G^{10}. G^{10} N1H has a cross peak with peak which is not visible on the diagonal and could not be assigned (k). The experiment was carried out at 500 MHz using a mixing time of 250 ms and at 7 °C.

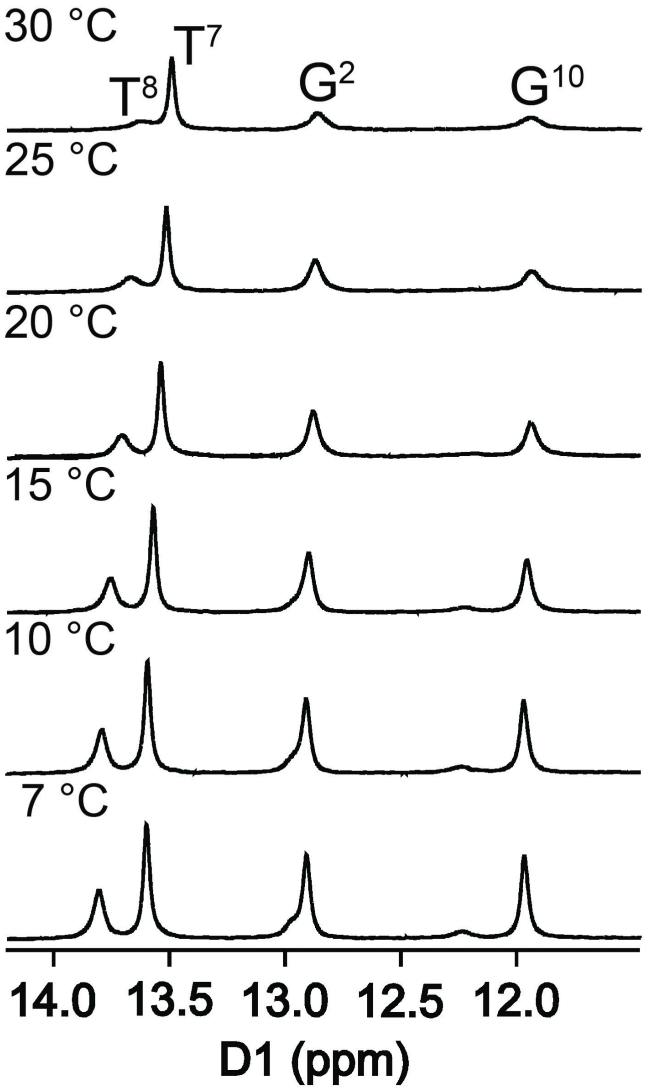

Figure S8. One-dimentional NMR spectra, showing the imino proton resonances for the DDD-XY duplex as a function of temperature. Note that the imine nitrogen of the O^6-Bn-dG nucleotide X^4 is not protonated at neutral pH. The individual nucleotides are identified as superscripts. The experiments were carried out at 500 MHz.

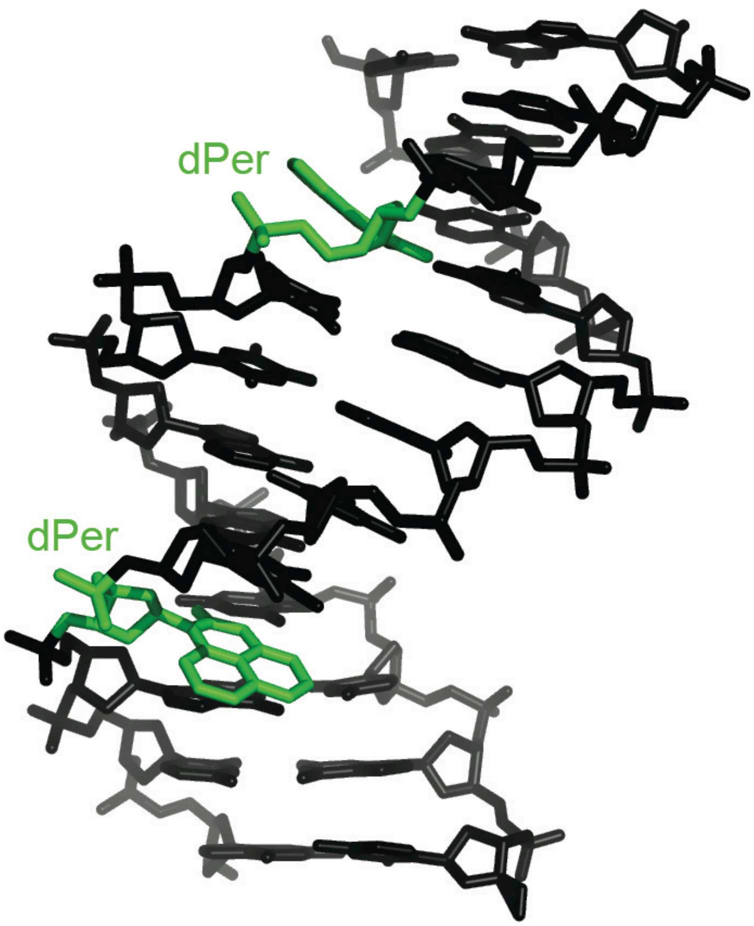

Figure S9. The average structure obtained from a series of rMD calculations for the DDD-GY duplex. dPer bases are shown in green. The dPer base is in the *anti* conformation about the glycosyl bond and it forms wobble pair with the complementary dG. The dPer ring is oriented in the major groove. Hydrogens are omitted on the picture for clarity.

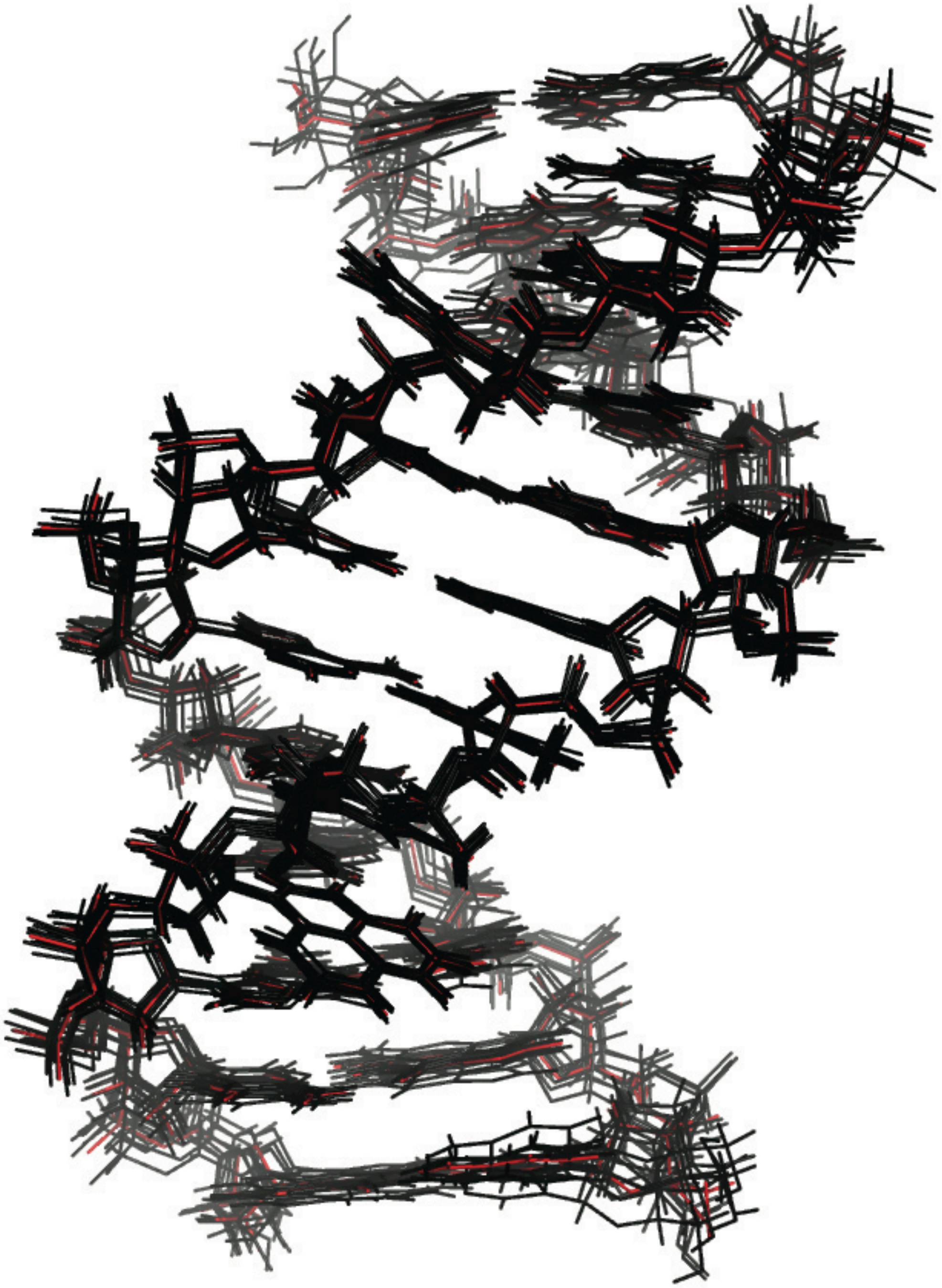

Figure S10. The superimposed nine structures obtained from a series of rMD calculations for the DDD-GY duplex. The average structure obtained from nine structures is shown in red.

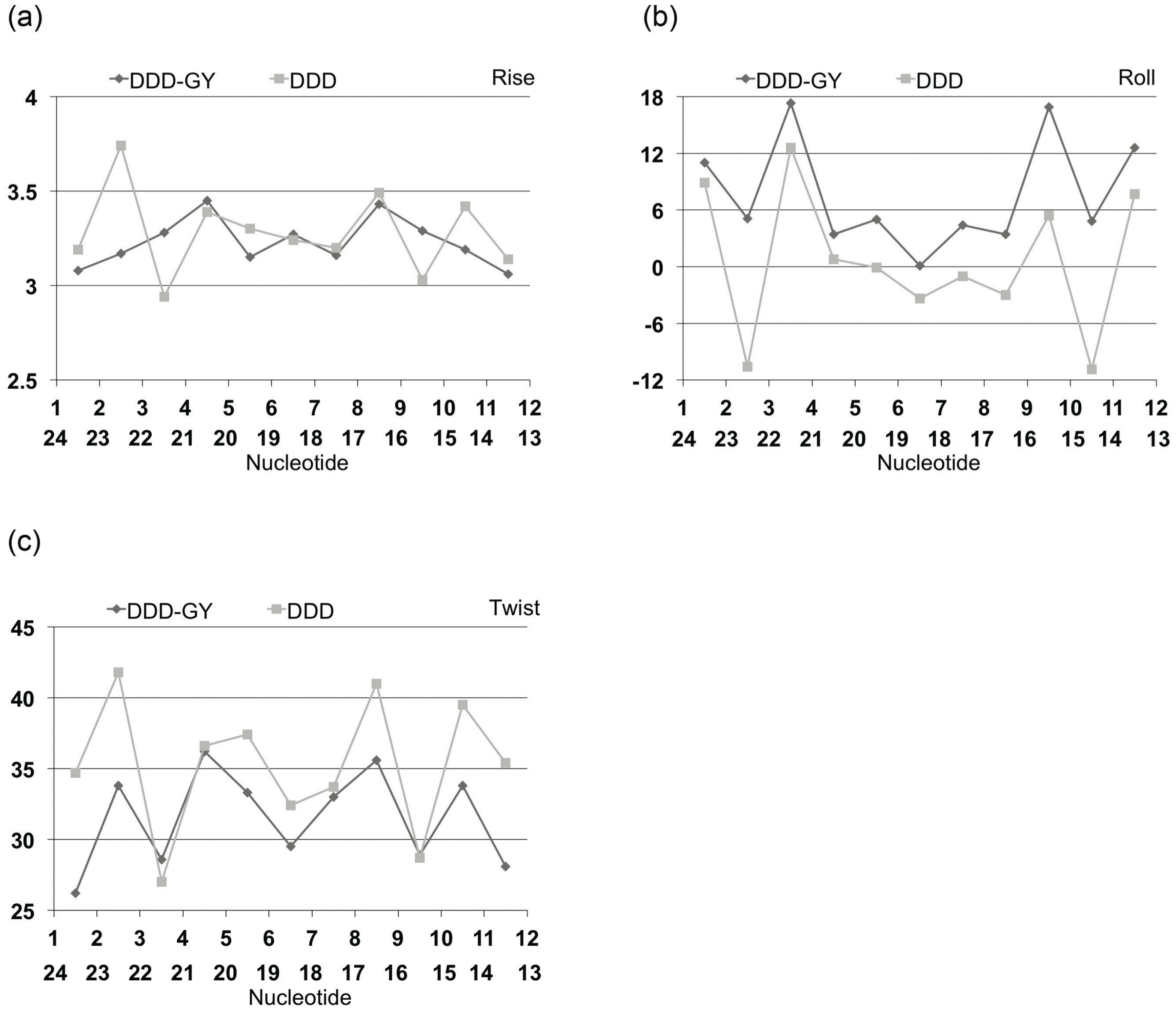

Figure S11. Interbase pair parameters: (a) helical rise, (b) roll and (c) twist for the DDD-GY, DDD (PDB entry 355D) duplexes.

(a)

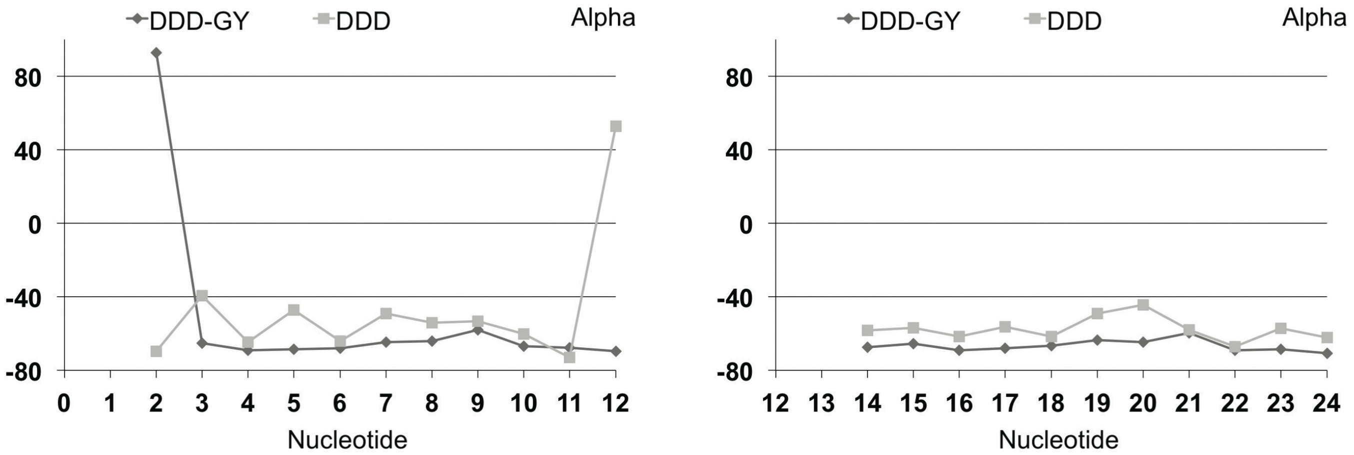

(b)

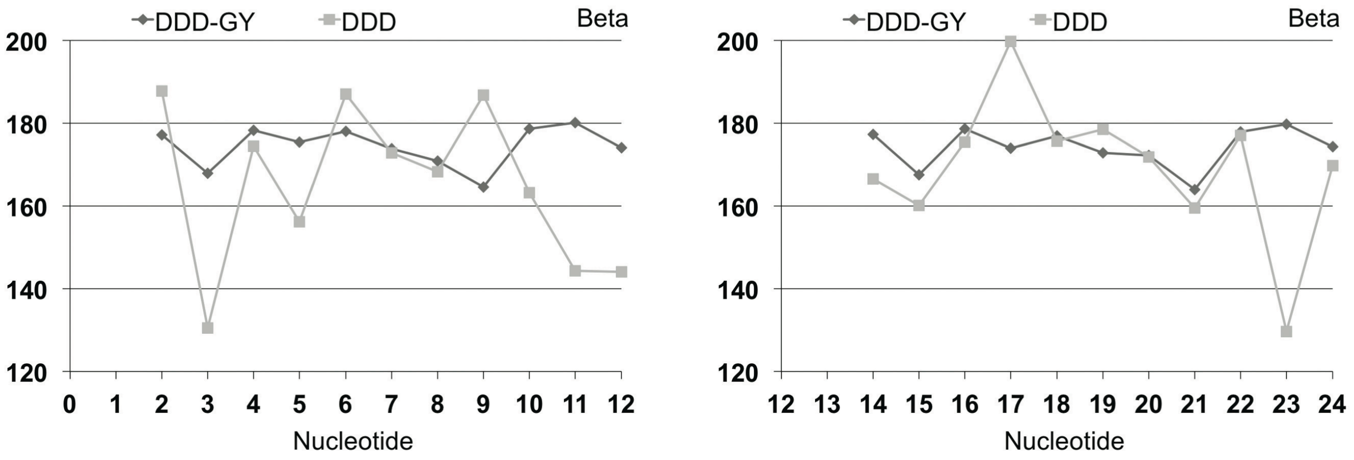

Figure S12. Comparison of backbone torsion angles (a) α and (b) β in the structures of the DDD-GY, DDD (PDB entry 355D) duplexes.

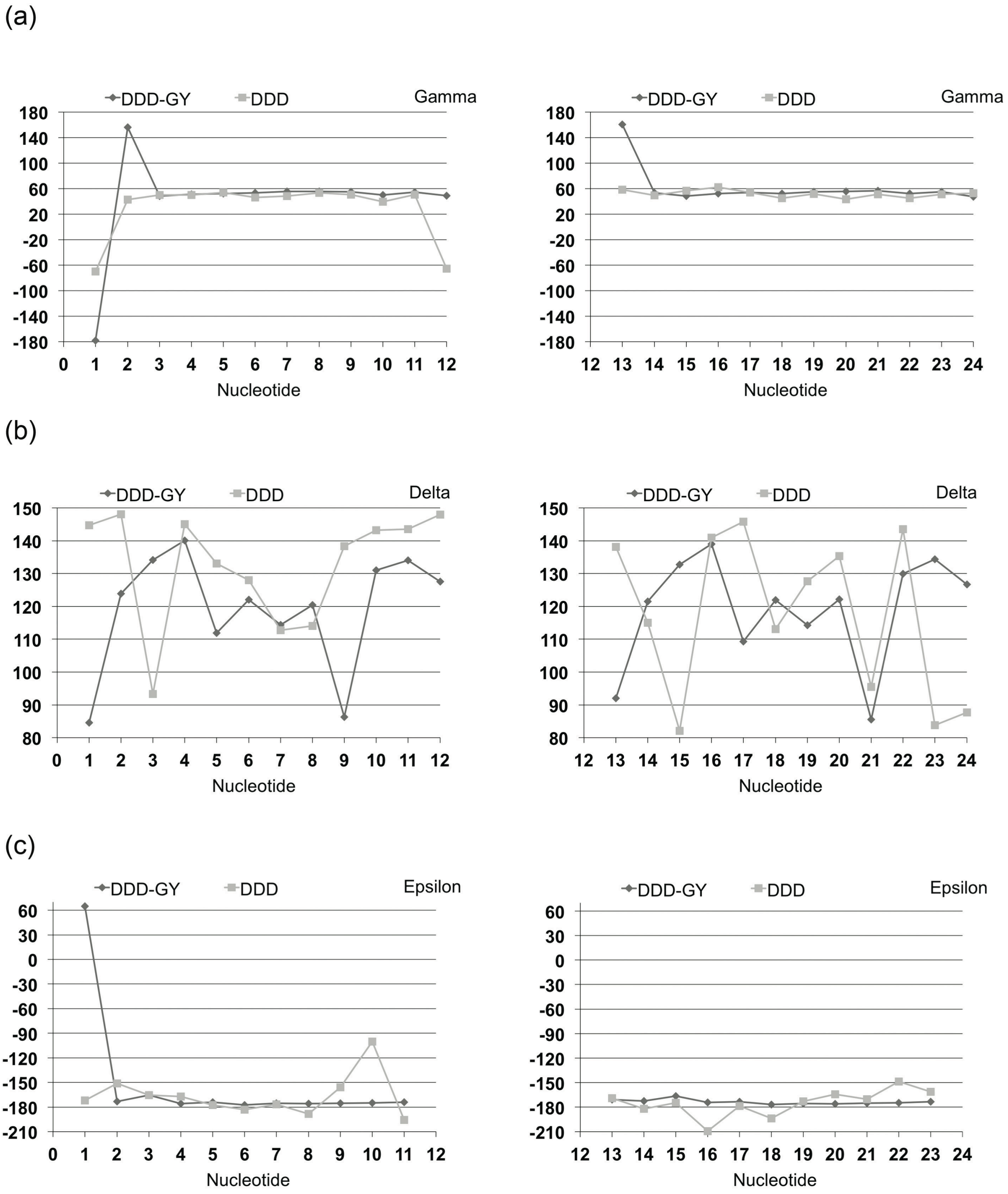

Figure S13. Comparison of (a) γ, (b) δ, (c) ε angles in the structures of the DDD-GY, DDD (PDB entry 355D) duplexes.

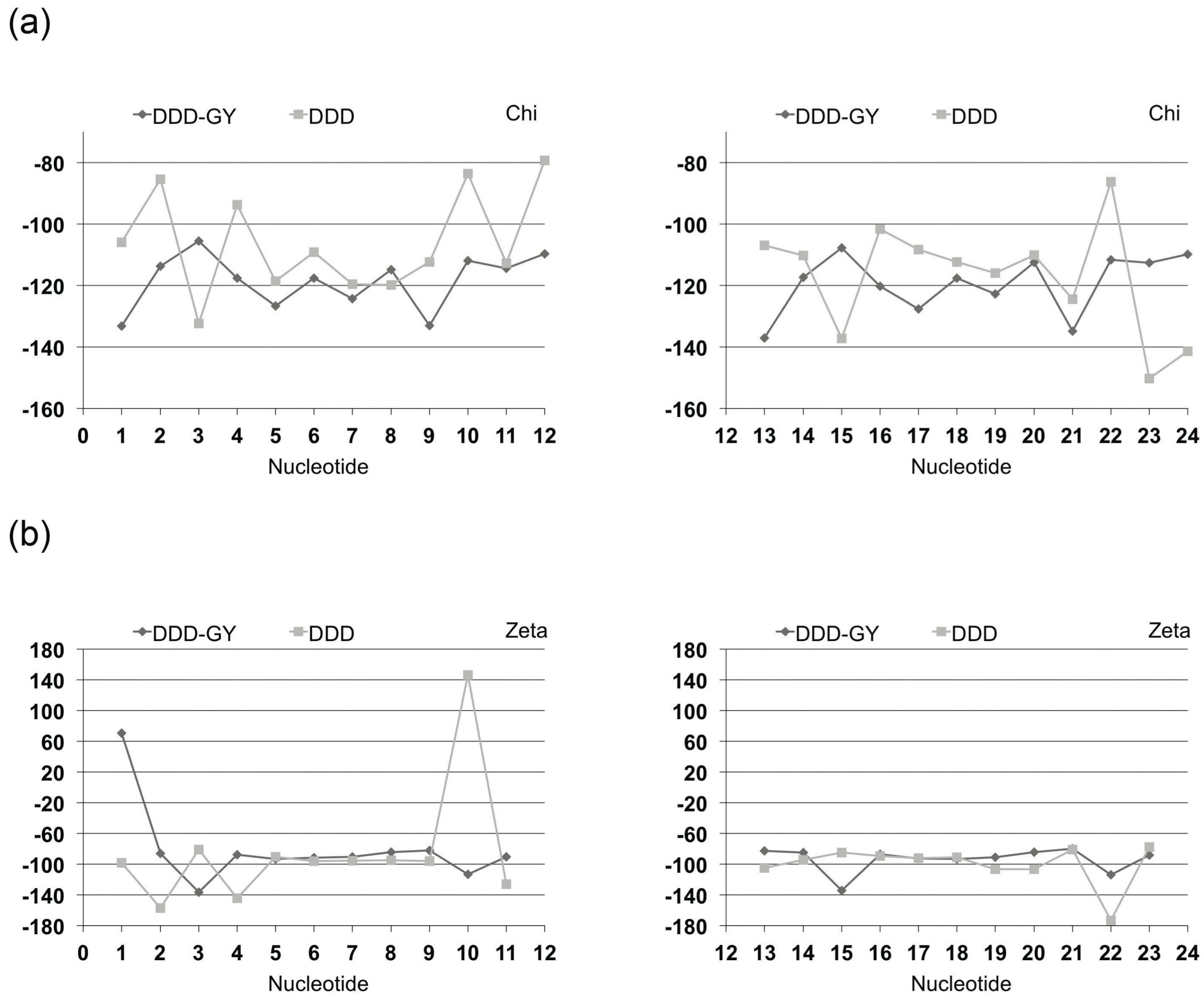

Figure S14. Comparison of (a) χ and (b) ζ angles in the crystal structures of the DDD-GY, DDD (PDB entry 355D) duplexes.
